# A systematic review on estimating population attributable fraction for risk factors for small-for-gestational-age births in 81 low- and middle-income countries

**DOI:** 10.7189/jogh.12.04024

**Published:** 2022-03-26

**Authors:** Sabi Gurung, Hannah Hanzi Tong, Emily Bryce, Joanne Katz, Anne CC Lee, Robert E Black, Neff Walker

**Affiliations:** 1 Department of International Health, Bloomberg School of Public Health, Johns Hopkins University, Baltimore, Maryland, USA; 2 Department of Pediatric Newborn Medicine, Global Advancement of Infants and Mothers (AIM), Brigham and Women’s Hospital, Boston, Massachusetts, USA

## Abstract

**Background:**

Small for gestational age (SGA) is a public health concern since it is associated with mortality in neonatal and post-neonatal period. Despite the large magnitude of the problem, little is known about the population-attributable risk (PAR) of various risk factors for SGA. This study estimated the relative contribution of risk factors for SGA, as a basis for identifying priority areas for developing and/or implementing interventions to reduce the incidence of SGA births and related mortality and morbidity.

**Methods:**

We conducted a literature review on 63 potential risk factors for SGA to quantify the risk relationship and estimate the prevalence of risk factors (RFs). We calculated the population-attributable fraction for each of the identified RF for 81 Countdown countries and calculated regional estimates. Twenty-five RFs were included in the final model while extended model included all the 25 RFs from the final model and two additional RFs.

**Results:**

In the final and extended models, the RFs included in each model have a total PAF equal to 63.97% and 69.66%, respectively of SGA across the 81 LMICs. In the extended model, maternal nutritional status has the greatest PAF (28.15%), followed by environmental and other exposures during pregnancy (15.82%), pregnancy history (11.01%), and general health issues or morbidity (10.34%). The RFs included in the final and extended model for Sub-Saharan African (SSA) region have a total PAF of 63.28% and 65.72% of SGA, respectively. In SSA, the top three RF categories in the extended model are nutrition (25.05%), environment and other exposure (13.01%), and general health issues or morbidity (10.72%), while in South-Asia’s it was nutrition (30.56%), environment and other exposure (15.27%) and pregnancy history (11.68%).

**Conclusions:**

The various types of RFs that play a role in SGA births highlight the importance of a multifaceted approach to tackle SGA. Depending on the types of RFs, intervention should be strategically targeted at either individual or household and/or community or policy level. There is also a need to research the mechanisms by which some of the RFs might hinder fetal growth.

Small for gestational age (SGA), a common proxy for intrauterine growth restriction, is defined as newborns whose size is smaller than normal for their gestational age, commonly defined as birth weight below the 10th percentile for gestational age based on a sex-specific reference population [1]. Fetal ultrasonography is a common method to diagnosis intrauterine growth restriction (IUGR) in high income settings since ultrasonography is widely available; however, SGA at birth is the most commonly used indicator in resource constrained low- and middle-income countries (LMICs) [2]. IUGR is defined as a fetus whose estimated weight is below the 10th percentile for its gestational age and the abdominal circumference is below the 2.5th percentile [3].

Understanding fetal growth is complex since the etiology and manifestations are heterogeneous, and so are methodologies and outcomes [4]. About 21% of neonatal deaths in LMICs are attributable to children being small for their gestational age [5]. SGA is a public health concern since it is associated with mortality in neonatal [5], post-neonatal (29–365 days after birth) [6], and even in later years (up to 18 years of age) [4]. SGA infants have an increased risk of post-natal infections due to poor immunity [7], post-neonatal mortality due to infections or neurologic disease [4] and altered metabolic functions through decreasing insulin sensitivity [8]. SGA is also associated with poor growth and development. Compared to children born adequate size for gestational age, children born SGA have 2.32 and 2.36 higher odds of stunting and wasting respectively in LMICs [9]. SGA infants with poor growth and development can also have long term implications since they are at risk of having lower mental and cognitive performance in adulthood [10,11]. These factors not only affect their individual economic productivity but also hinder them from contributing to their country’s economy. As defined by the Intergrowth Standard that used birth weight below 10th centile cut-off, about 19% of live births (23.3 million neonates) in LMICs are estimated to be SGA, indicating the high global burden of SGA [5]. Regionally, South Asia (SA) has the highest prevalence of SGA (34.2%), followed by Sub-Saharan Africa (16.5%) [5]. Despite the large magnitude of the problem, little is known about the population-attributable risk (PAR) of risk factors (RFs) for SGA. The dearth and poor quality of data on birthweight and gestational age, especially in LMICs, are barriers to assessing PARs for SGA. Additionally, the various methods used to ascertain gestational age and the use of various reference populations to define SGA will affect any estimation of the prevalence of SGA. To reduce the prevalence of SGA and SGA-related mortality and morbidity in LMICs, it is crucial to get a better understanding of the risk factors and causes of SGA. Understanding the relative importance and degree of such factors is a key piece of information that will not only help understand the relative public health implications of various risk factors but can also help develop and prioritize strategies or interventions to reduce SGA related poor health outcomes.

In this paper, we use available evidence to: 1) identify the possible RFs for SGA; 2) quantify the risk relationships; 3) assess the quality of the evidence supporting these relationships; and 4) estimate the prevalence of RFs. We then use population-attributable fractions (PAFs) to estimate the contribution of individual RFs to SGA in LMICs. The purpose of this study is to estimate the relative contribution of RFs for SGA, as a basis for identifying priority areas for developing and/or implementing interventions to prevent SGA births and related mortality and morbidity.

## METHODS

We conducted a literature review on potential RFs for SGA, defined risk relationships and estimated prevalence for established RFs for SGA, followed by which we calculated the PAF. We ran the analyses for the 81 Countdown countries [12] and used the World Health Organization’s (WHO) list to classify the countries into six regions: Africa (43 countries), Region of the Americas (12 countries), South East Asia (8 countries), Eastern Mediterranean (8 countries), Europe (5 countries), Western Pacific (5 countries). In addition to the overall estimate of 81 countries, we also presented results for South Asia with 5 countries (Bangladesh, Bhutan, India, Nepal, Pakistan) and Sub-Saharan Africa (43 countries). Although SGA definition varied across studies, the most common definition was birth weight below the 10th percentile for gestational age based on a sex-specific reference population [1]. Other SGA definitions were birth weight 2 standard deviations below the standard for gestational age or a birth weight for gestational age below the 5th or 3rd centile [13,14], using standard or unknown reference.

## Risk factors

**Figure 1:**
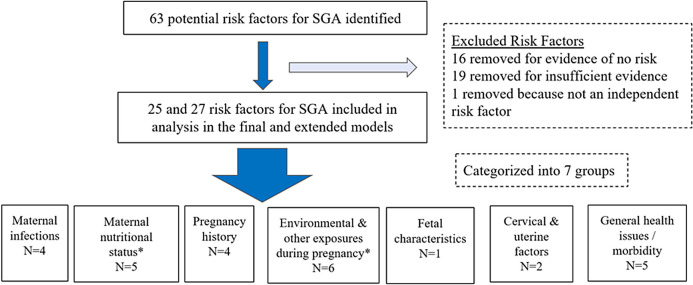
Methods for identifying risk factors (RF) for small-for-gestational age babies. Note: The categories with an asterisk sign have an additional RF included only in the extended model (anemia in the maternal nutritional status category and indoor air pollution in the environmental and other exposures during pregnancy category).

**Identifying potential RFs for small for gestational age infants**: We identified and confirmed RFs for SGA by generating potential RFs developed by experts in the field and published literature [3,15-20]. Additionally, we conducted a literature review to identify other possible risks not included in these prior reports. These potential RFs were then further examined to determine the risk relationship with SGA birth. The evidence supporting decisions on inclusion or exclusion of a RF in the modeling process are described in detail in Section S3 in the **Online Supplementary Document**. As shown in **Figure 1**, a total of 63 potential RFs were explored for their association with SGA and details of all these RFs are presented at Section S3 in the **Online Supplementary Document**. RFs were excluded from the models for three reasons: (1) adequate evidence of the absence of a risk relationship with SGA; (2) insufficient evidence or inconsistent conclusions about the association between the RF and SGA; or (3) the RF is not independent of other SGA RFs, ie, the RF is either an intermediate outcome between another RF and SGA, or a composite of other RFs already considered in the models. Additionally, the published literature on potential SGA RFs were assessed for their quality to determine if the RFs should be included in our models.

**Quality of risk estimates**: We conducted a literature review on all the 63 potential RFs for SGA to quantify the risk relationship. Observational studies with the potential RFs for SGA and intervention trials that explored the association between potential RFs and the birth outcomes (SGA) were identified and included in the **Online Supplementary Document**. We used the PRISMA and GRADE checklist as references to create two revised checklists: one for observational studies, while the other for intervention studies. The checklists were used to give scores based on factors such as the design of studies; study site; definition of predictor and outcome variables; adjustment of confounders; heterogeneity; and magnitude of the association (Tables S2A and S2B in **Online Supplementary Document**). This allowed the quality of the sources for observational studies and intervention studies to be graded separately, producing two quality scores. Thus, RFs that did not have published articles with evidence of an intervention delivered during the antenatal period only had the quality score for the observational risk estimate. The maximum possible score was 14 and 17 for observational risk estimates and intervention impact estimates, respectively. For observational risk sources studies, a score greater than 10 was deemed “high” quality, studies with scores of 8 to 10 were “medium” quality and those with a score less than 8 were considered to be of “low” quality. The cut-offs for high, medium and low quality for intervention studies were greater than 13, 10 to 13 and below 10, respectively. Since there is no standard cut offs score to grade the quality of the papers, we created our cut-offs after reviewing published systematic and meta-analysis papers that used PRISMA or GRADE checklist to grade the quality of the papers. Using these quality scores, we graded the overall quality of evidence for each RF using a six-level categorization (high, medium-high, medium, medium-low, low, and very low) to decide on the inclusion or exclusion of the RF in our model. The rule for assigning the overall quality is presented at Table S2C in the **Online Supplementary Document**.

**Risk estimates in the analysis model**: Based on the evidence and quality of published articles on RFs, a total of 25 RFs are included in the final model for each country (Section 3 in the **Online Supplementary Document**). We have also developed an extended model that includes additional RFs, when medium/ high quality meta-analysis and systematic review papers show some degree of evidence of association between a RF and SGA (borderline but statistically insignificant evidence of association). This extended model contains all the 25 RFs included in the final model and two additional RFs that fits the criteria (indoor pollution and anemia). Although individual studies conducted in LMICs showed inconsistent association between indoor pollution and SGA, a meta-analysis and systematic review showed significant association between indoor pollution and low birth weight (Section S3 in **Online Supplementary Document**). Additionally, there was no evidence of intervention effect between indoor pollution and SGA. For anemia, two meta-analysis papers published in 2013 and 2016, which analyzed only LMICs did not find a statistically significant association between anemia and SGA, but another meta-analysis published in 2019 that included both LMICs and high-income countries found a statistically significant association between anemia and SGA (Section S3 in **Online Supplementary Document**). These two RFs had wide heterogeneity in study results as well as variability in conclusions of meta-analyses that used different inclusion and exclusions criteria. Also, since both these possible RFs are fairly prevalent in LMICs, we felt they should be included in the extended model since future work may clarify the risk relationship for these two factors. Thus, the final model includes 25 RFs while the extended model has 27 RFs. Overall and regional level PAFs of the final and extended models are also presented based on the nature of these RFs and depending on the nature of the RFs, they are further categorized into seven categories outlined below:

- Nutritional status: Vitamin D deficiency, low pre-pregnancy BMI, low gestational weight gain, short maternal stature (<145 cm), and anemia*
- Maternal infection: Malaria, Human Immunodeficiency Virus (HIV), chlamydia trachomatis, and trichomonas vaginalis
- Pregnancy history: Maternal age <18 and primiparity, and maternal age 18-35 and primiparity, short birth intervals (< 18 months, birth interval 18- <24 months), birth interval 60 months or greater
- Uterine and cervical factors: Endometriosis and adenomyosis
- Environmental and other exposures during pregnancy: Alcohol consumption, smoking during pregnancy, second-hand smoking, ambient air pollution, heavy physical workload, and indoor air pollution*
- General health issues or morbidity: Hypertension, pre-eclampsia, subclinical hypothyroidism, inflammatory bowel disease, and anxiety
- Fetal Characteristics: Twin pregnancy

Note: The RFs with an Asterisk (*) sign are included only in the extended model.

### Risk factor prevalence estimates

**Estimates of prevalence of risk factors:** We gave priority to prevalence estimates of each RF from recent nationally representative household surveys like the Demographic Health Survey (DHS) where available. If RFs were not measured in household surveys, we searched for recent country-level prevalence estimates, and often defaulted to the estimates produced by the Institute for Health Metrics and Evaluation (IHME) / Global Burden of Disease [21] or Lives Saved Tool (LiST) database (https://www.livessavedtool.org/) [22]. The LiST database has compiled data from multiple surveys like DHS, national surveys, Multiple Indicator Cluster Survey, Fertility and Family Survey. When country-level data were not available, we used meta-analyses to calculate country or regional prevalence estimates of the RFs in such cases by compiling country-specific prevalence as the regional average of those with national data. When these data were not available, meta-analyses that produced regional prevalence estimates for the RFs were used. We used LiST to get prevalence estimates for eight RFs, IHME/GBD for six RFs, the UN database for six RFs, and systematic and meta-analysis for seven RFs. Country-level estimates were used for the majority of the RFs where available (N = 18), while the rest of the RFs used global/regional non-nationally representative country data (N = 9). Details on the source of prevalence estimate and quality assessment for each of the RF’s prevalence estimates are described in Section S4 of the **Online Supplementary Document**.

**Quality of prevalence estimates:** We defined three categories for the quality of prevalence estimates, based on the source of data. A “high” quality source was defined as country-specific prevalence data with upper and lower bound estimates collected via nationally representative surveys during pregnancy. A “medium” quality source was country-specific prevalence data projected from a model developed by sources like IHME, LiST. A “low” quality source was regional data available by meta-analysis studies and used for the country level estimates or if the prevalence estimate was based on women of reproductive age and not solely on pregnant women.

**Treatment adjustment of prevalence estimates:** Based on the availability of evidence from published intervention trials, two RFs, ie, malaria and pre-eclampsia were found to have interventions during pregnancy that could reduce SGA (Section S3 in **Online Supplementary Document**). The estimates adjust for interventions that prevent the effect of the RF (malaria, pre-eclampsia). For these factors, the prevalence was adjusted to account for this treatment effect using the following equation:

Adjusted prevalence = (unadjusted prevalence × coverage of treatment × efficacy of treatment)

Data on treatment coverage for pre-eclampsia (Magnesium sulphate) is from the LiST database and were adjusted for ante-natal care coverage. Similarly, data on treatment coverage for malaria (IPTp- Intermittent Preventive Therapy during Pregnancy) is from LiST [22] and were drawn from nationally representative household surveys. Efficacy of the treatment for these two RFs are from intervention studies [23,24]. We did not adjust for treatment effect for some of the RFs such as vitamin D deficiency and anemia. This is because, in the case of vitamin D deficiency we used the adjusted risk estimates from the intervention trials directly in our analysis model, while for anemia there was a lack of association between iron-folic acid supplementation and SGA.

**Calculation of country level SGA estimates**: To calculate country level SGA estimates, we used the World Population Prospects 2018 estimates of live births for the year 2020 [25] and SGA rates that defined SGA as infants weighing less than the 10th centile birth weight for gestational age and sex with the multiethnic, INTERGROWTH-21st birth weight standard [5]. Of the 81 countries that were included in the analyses, only one country did not have the SGA rate (South Sudan) and therefore the regional estimate was used for that country.

**Calculation of population attributable fraction**: The PAF describes the proportional reduction in SGA in the overall population if the population with a certain RF does not have the RF (non-risk category) [26]. If risk relationships were reported as odds ratios with 95% confidence-intervals, we converted it to risk ratios using the following calculations (Equation 1) [27]. Risk estimates of the association of individual RFs to SGA was calculated in the following steps and this process was completed for both the final model (25 RFs) and the extended model (27 RFs).

1. Equation 2 (Levin’s formula) [PAF_u_] estimated the independent PAF for each of the RF for each country and did not adjust for multiple RFs [28,29].
2. Equation 3 [PAF_t_] calculated a combined estimate of the population attributable fraction for all RFs in that particular country (25 RFs in the final model and 27 indicates the RFs in the extended model) [30].
3. Equation 4 [PAF_a_] calculated final corrected population attributable fractions of each of the RF for each country. Using these PAFs, we produced regional and global totals by estimating the number of SGA births in each country associated with a RF and then summing these values for all 81 LMICs and for subsets of countries within each of the region for final (Equations 5a, 5b) and extended models (Equations 5c, 5d).
4. Equation 6 obtained the final percent of SGA attributable to each RF in the 81 countries and in the two regions.

**Equation 1:** Converting odds ratio to relative risk (RR)

*RR* = *Odds ratio* / [(1 – *Prevalence_reference_ group*) + (*Prevalence_reference_ group* × *Odds ratio*)]

**Equation 2:** Individual Population Attributable Fraction (PAF):

For all RFs:

*PAF_μ_ = P(F)(RR – 1) */* *[*1 +P(F)(RR – 1)*]

where P(F) is the RF prevalence and RR is the relative risk associated with the factor, comparing SGA births in the exposed and unexposed groups.

**Equation 3:** Combined PAF for each country

a. Final model:



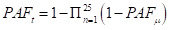



b. Extended model:



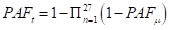



where 25 indicates 25 RFs in the final model, while 27 indicates the RFs in the extended model.

**Equation 4:** Correction for overestimating PAF when only considering single RFs

PAF_a_ = (Individual PAF_μ_ / ∑PAF_μ1-27 or 25_) × PAF_t_

25 RFS for final model and 27 RFs for extended model.


**Equation 5:**

Equation for final model:

5a. Global estimate: ∑ 1-25RFs # SGA associated with the RF across all 81 countries

5b. Then, for each region: ∑ # of SGA associated with the RF for all countries in region

Equation for extended model:

5c. Global estimate: ∑ 1-27RFs # SGA associated with the RF across all 81 countries

5d. Then, for each region: ∑ # of SGA associated with the RF for all countries in region

**Equation 6:**

% of SGA attributable to a RF in 81 countries = # of SGA per RF (equation 5a) / total number of SGA in 81 countries

% of SGA attributable to a RF in a region = # of SGA per RF (equation 5b) / total number of SGA in the region

**Equation 7:**

Minimum and maximum estimates of PAF = Attributable fraction of each RF × (1 – total attributable fraction)

## RESULTS

### Risk associations

**Table 1** summarizes the identification of factors proposed by previous research papers as potential RFs for SGA births. Thirty-six of these RFs were excluded from the analysis because of reasons described in the Methods Section. The supplementary materials provide summaries of available evidence for all 63 potential SGA RFs. The summary of the excluded RFs is presented at Table S1B in the **Online Supplementary Document**, and it shows the RRs for SGA for the 27 RFs currently supported by available evidence, quality of evidence supporting the risk estimates, estimates of RF prevalence, and quality of the prevalence data sources. The estimated RRs of these 27 RFs for SGA range from 1.03 (exposure to second-hand smoking during pregnancy) to 3.98 (twin pregnancy). The risks due to maternal age and parity were presented in three categories based on the available studies that combined age and parity [40]. The highest risks associated with SGA (>1.50 RR) are maternal infections (HIV, *Trichomonas vaginalis*), young primipara women (<18 years), short maternal stature (<145 cm), pre-eclampsia, smoking during pregnancy, twin pregnancies, and maternal fibroid (adenomyosis).

**Table 1:** Summary of the risk factors included in the model for calculating population attributable fraction of small-for-gestational age

Risk factor	Relative risk (95% CI)*	Observational risk quality	Intervention quality	Overall quality	Agreement on effects between observational and intervention studies	Prevalence	Prevalence quality
**Maternal infection**							
Malaria	1.10 (1.02-1.18) [23]	High	Medium	Medium-High	Yes	Country specific†	Medium
HIV	1.64 (1.29-2.09) [31]	High	NA	Medium	NA	Country specific	High
*Chlamydia*	1.13 (1.05-1.24) [32]	Medium	Low	Medium-Low	No	Country specific	High
*Trichomonas vaginalis*	1.51 (1.32-1.73) [33]	Medium	NA	Low	NA	Country specific	High
**Environment and other exposures during pregnancy**							
Heavy physical workload	1.07 (1.00-1.13) [34]	High	NA	Medium	NA	Regional & country level data	Medium-low
Secondhand smoking	1.03 (1.00-1.07) [35]	Medium	Low	Medium-Low	No	Country specific	Medium
Ambient air pollution	1.12 (1.08-1.16) [36]	High	Low	Medium	Yes	Country specific	Medium
Indoor air pollution‡	1.23 (1.01-1.49) [37]	High	Low	Medium	NA	Country specific	Medium
Smoking	1.86 (1.81-1.91) [35]	Medium	NA	Low	NA	Country specific	Medium
Alcohol consumption	1.17 (1.03-1.32) [38]	Medium	NA	Low	NA	Country specific	Medium-high
**Pregnancy history**							
Young maternal age <18 & Primiparity	1.70 (1.32-1.53) [39]	High	NA	Medium	NA	Country specific	High
Young maternal age 18-35 & Primiparity	1.32 (1.25-1.39) [40]	High	NA	Medium	NA	Country specific	High
Birth interval of <18 months	1.44 (1.27-1.63) [40]	High	NA	Medium	NA	Country specific	High
Birth interval of 18-<24 months	1.20 (1.03-1.40) [40]	High	NA	Medium	NA	Country specific	High
Birth interval of over 60 months	1.15 (1.05-1.25) [40]	High	NA	Medium	NA	Country specific	High
**Maternal nutritional status**							
Low maternal stature	2.03 (1.76-2.35) [41]	Medium	NA	Low	NA	Country specific	Medium
Low pre-pregnancy body mass index	1.21(1.12-1.31) [42]	Medium	High	High	NA	Country specific	High
Low gestation weight gain	1.36 (1.31-1.43) [39]	High	NA	Medium	NA	Regional & country level data	Low
Vitamin D deficiency	1.39 (1.01-1.92) [43]	High	Medium	Medium	Yes	Global & country level data	Low
Anemia‡	1.07 (1.00-1.05) [44]	Medium	High	High	Yes	Country specific	High
**Placenta-related health issues / maternal morbidity**							
Pre-eclampsia	1.63 (1.55-1.71) [45]	Medium	High	High	NA	Regional†	low
Hypertension‖	1.38 [45]	Medium	High	High	No	Country specific	High
Subclinical hypothyroidism	1.23 (1.04-1.46) [46]	High	NA	Medium	NA	Global	Low
Anxiety disorder	1.35 (1.17-1.54) [47]	Medium	Low	Medium-Low	No	Global & country level data	Low
Inflammatory bowel disease	1.36 (1.16-1.60) [38]	Medium	NA	Low	NA	Regional	Low
**Uterine and cervical factors**							
Endometriosis	1.25 (1.01-1.54) [48]	Medium	NA	Low	NA	Country specific	High
Adenomyosis	2.55 (1.58-3.78) [48]	Medium	NA	Low	NA	Global	Low
**Fetal characteristics**							
Twin pregnancy	3.98 [49] (not reported)	Medium	NA	Low	NA	Country-specific	Medium

CI – confidence interval, NA – not applicable

*The risks included in the models have all been converted to relative risks if initially reported as odds ratios. See supplementary materials for more details.

†Prevalence was adjusted for treatment effects.

‡RFs that were included only in the extended model.

‖The study did not have 95% confidence interval for the result estimates.

### Risk factor prevalence

**Table 1** summarizes the prevalence of the RFs and the quality of the estimates. Country-specific prevalence data were available for more than half of the RFs (19 of 27), and the quality of prevalence data varied widely across factors. The point estimate for the risk was based on observational data for all RFs except those where the RF was only due to a nutrient deficiency. For example, in the case of vitamin D deficiency, the inverse of the intervention trial was used to represent the risk estimate and details on this approach are described at Sections S1 and S3 in the **Online Supplementary Document**.

### Population attributable fraction of small for gestational age

**Table 2** shows the two models (final and extended) with the estimated PAF of SGA for each RF in the overall 81 LMICs. Both the models include the same RFs, except for the extended model, which has anemia and indoor air pollution as additional RFs in its model. In the final and extended models, the RFs have a total PAF equal to 63.97% and 69.66%, respectively of SGA across the 81 LMICs. The remaining 36.03% and 30.34%, respectively account for unknown/ unattributed PAF. The leading RFs for SGA in the final model are vitamin D deficiency (13.19%), low gestational weight gain (7.90%), hypertension (6.02%), primiparous women of age 18-35 years (5.16%), short height (4.46%), and ambient air pollution (4.32%). Similarly, the extended model has the same top leading RFs reported in the final model, except for ambient air pollution which gets replaced by indoor air pollution (7.96%).

**Table 2:** Population attributable fraction of small-for-gestational age for overall 81 LMICs, in the final and extended models*

Risk factor	Overall estimates in final model (min-max)	Overall estimates in extended model (min-max)
**Maternal infection:**		
Malaria	0.35% (0.00%-2.55%)	0.32% (0.00%-2.36%)
HIV	0.46% (0.00%-11.19%)	0.43% (0.00%-10.78%)
Chlamydia	0.15% (0.07%-0.40%)	0.14% (0.07%-0.37%)
*Trichomonas vaginalis*	1.24% (0.67%-3.89%)	1.15% (0.64%-3.61%)
**Environment and other exposures during pregnancy**		
Alcohol consumption	0.13% (0.04%-1.24%)	0.12% (0.04%-1.25%)
Heavy physical workload	1.88% (0.30%-3.86%)	1.75% (0.28%-3.56%)
Smoking during pregnancy	1.74% (0.23%-7.39%)	1.63% (0.22%-6.82%)
Ambient air pollution	4.32% (0.85%-5.44%)	4.03% (0.78%-5.11%)
Secondhand smoking	0.37% (0.03%-0.99%)	0.35% (0.03%-0.94%)
Indoor air pollution		7.96% (0.13%-11.42%)
**Pregnancy history:**		
Maternal age <18 & Primiparity	1.89% (0.36%-5.75%)	1.76% (0.34%-5.33%)
Maternal age 18-35 & Primiparity	5.16% (1.63%-7.96%)	4.82% (1.49%-7.83%)
Birth interval <18 months	1.94% (0.59%-3.74%)	1.81% (0.54%-3.50%)
Birth interval 18-<24 months	1.32% (0.50%-2.16%)	1.23% (0.48%-2.02%)
Birth interval 60 months or greater	1.49% (0.02%-5.13%)	1.39% (0.02%-4.87%)
**Maternal nutritional status:**		
Short height	4.46% (0.14%-13.67%)	4.18% (0.13%-12.94%)
Low pre-pregnancy Body Mass Index	2.46% (0.34%-3.27%)	2.30% (0.33%-3.06%)
Low gestational weight gain	7.90% (4.31%-13.91%)	7.35% (4.21%-12.80%)
Vitamin D Deficiency	13.19% (0.54%-15.12%)	12.31% (0.50%-14.16%)
Anemia		2.01% (0.98%-2.81%)
**General health issues/ morbidity:**		
Hypertension	6.02% (3.81%-7.89%)	5.62% (3.73%-7.20%)
Pre-eclampsia	0.97% (0.40%-1.56%)	0.90% (0.39%-1.43%)
Subclinical hypothyroidism	0.45% (0.42%-0.51%)	0.42% (0.40%-0.51%)
Inflammatory Bowel Disease	0.01% (0.01%-0.14%)	0.01% (0.01%-0.13%)
Anxiety	3.63% (2.68%-6.48%)	3.39% (2.47%-6.01%)
**Uterine and cervical factors:**		
Endometriosis	0.14% (0.09%-0.34%)	0.13% (0.08%-0.31%)
Adenomyosis	0.26% (0.24%-0.29%)	0.24% (0.23%-0.30%)
**Fetal characteristics:**		
Twin pregnancy	2.07% (1.28%-13.78%)	1.93% (1.18%-13.24%)
**Total population attributable fraction**	**63.97%**	**69.66%**

*Min/Max is the minimum/maximum of the PAF_α_ of the RF (Equation 4) among the 81 countries.

**Table 3** shows the two models (final and extended) with estimated percent of SGA attributed to each RF for Sub-Saharan Africa (SSA) and South Asia (SA). The RFs included in the final and extended model for SSA region have a total PAF of 63.28% and 65.72% of SGA, respectively, where the region’s leading RFs for SGA are similar to those reported at the overall level. The five leading RFs in the SSA region’s final model include vitamin D deficiency (11.07%), low gestational weight gain (10.78%), hypertension (6.49%), ambient air pollution (3.57%), and anxiety (3.32%). The region’s extended model has the same top leading RFs for SGA as reported in the region’s final model, except for anxiety, which gets replaced by indoor air pollution (5.38%). In the South Asian region, the RFs included in the final and extended models have a total PAF of 65.24% and 70.50% of SGA, respectively. In the region’s final model, the top five leading RFs for SGA are mostly nutrition related: vitamin D deficiency (14.67%), low gestational weight gain (6.62%), primipara women of 18-35 years (6.07%), short maternal stature (6.06%), and ambient air pollution (4.98%). Similarly, in the extended model, the top five leading RFs for SGA are the same as the region’s final model, except for ambient air pollution, which gets replaced by indoor air pollution (7.39%).

**Table 3:** Estimate percent of small-for-gestational age infants attributed to risk factors for Sub-Saharan region and South Asian Region, in the final and extended models*

Risk factor	Sub Saharan Africa		South Asia region	
	**Final model. Population attributable fraction (min-max)**	**Extended model. Population attributable fraction (min-max)**	**Final model. Population attributable fraction (min-max)**	**Extended model. Population attributable fraction (min-max)**
**Maternal infection:**				
Malaria	1.13% (0.00%-2.55%)	1.04% (0.00%-2.36%)	0.03% (0.00%-0.03%)	0.03% (0.00%-0.03%)
HIV	1.48% (0.00%-11.19%)	1.38% (0.00%-10.78%)	0.05% (0.00%-0.06%)	0.04% (0.00%-0.06%)
*Chlamydia*	0.20% (0.07%-0.36%)	0.19% (0.07%-0.35%)	0.10% (0.08%-0.20%)	0.10% (0.07%-0.18%)
*Trichomonas vaginalis*	2.15% (0.70%-3.89%)	1.98% (0.69%-3.61%)	0.86% (0.70%-0.91%)	0.80% (0.65%-0.85%)
**Environment and other exposures during pregnancy:**				
Alcohol consumption	0.23% (0.05%-0.47%)	0.21% (0.05%-0.43%)	0.04% (0.00%-0.05%)	0.04% (0.00%-0.04%)
Heavy physical workload	2.99% (0.77%-3.86%)	2.75% (0.76%-3.56%)	1.31% (1.20%-3.58%)	1.22% (1.12%-3.35%)
Smoking during pregnancy	1.25% (0.34%-4.56%)	1.16% (0.32%-4.20%)	1.72% (0.96%-6.21%)	1.61% (0.82%-5.80%)
Ambient air pollution	3.57% (1.22%-5.41%)	3.29% (1.12%-5.11%)	4.98% (4.55%-5.44%)	4.66% (4.35%-5.04%)
Secondhand smoking	0.22% (0.12%-0.73%)	0.21% (0.08%-0.69%)	0.38% (0.18%-0.73%)	0.36% (0.17%-0.68%)
Indoor air pollution		5.38% (1.05%-11.42%)		7.39% (5.20%-8.87%)
**Pregnancy history:**				
Maternal age <18 & Primiparity	2.59% (0.84%-4.39%)	2.39% (0.78%-4.03%)	1.58% (1.01%-5.75%)	1.48% (0.95%-5.33%)
Maternal age 18-35 & Primiparity	3.25% (1.63%-5.81%)	3.00% (1.49%-5.46%)	6.07% (4.19%-6.69%)	5.67% (3.91%-6.27%)
Birth interval <18 months	1.63% (0.59%-2.82%)	1.50% (0.54%-2.60%)	2.12% (0.73%-3.74%)	1.98% (0.68%-3.50%)
Birth interval 18-<24 months	1.39% (0.51%-2.82%)	1.28% (0.49%-1.87%)	1.31% (0.51%-1.88%)	1.22% (0.48%-1.76%)
Birth interval 60 months or Greater	0.95% (0.02%-3.89%)	0.88% (0.02%-3.80%)	1.42% (0.90%-3.89%)	1.32% (0.84%-3.60%)
**Maternal nutritional status:**				
Short height	1.54% (0.14%-4.59%)	1.42% (0.13%-4.22%)	6.06% (3.29%-7.20%)	5.67% (3.07%-6.67%)
Low pre-pregnancy Body Mass Index	1.66% (0.00%-2.65%)	1.53% (0.00%-2.51%)	3.08% (1.74%-3.27%)	2.88% (1.66%-3.06%)
Low gestational weight gain	10.78% (4.31%-13.91%)	9.92% (4.21%-12.80%)	6.62% (6.35%-6.80%)	6.19% (5.89%-6.42%)
Vitamin D deficiency	11.07% (0.54%-12.20%)	10.21% (0.50%-11.96%)	14.67% (10.90%-15.12%)	13.72% (10.11%-14.16%)
Anemia		1.95% (1.03%-2.64%)		2.10% (1.45%-2.22%)
**General health issues/morbidity:**				
Hypertension	6.49% (5.33%-7.89%)	5.98% (5.12%-7.20%)	5.81% (5.42%-6.77%)	5.43% (5.03%-6.32%)
Pre-eclampsia	1.35% (0.47%-1.56%)	1.25% (0.44%-1.43%)	0.82% (0.42%-0.99%)	0.77% (0.39%-0.92%)
Subclinical hypothyroidism	0.46% (0.42%-0.48%)	0.42% (0.40%-0.47%)	0.45% (0.43%-0.46%)	0.42% (0.40%-0.44%)
Inflammatory bowel disease	0.01% (0.01%-0.01%)	0.01% (0.01%-0.01%)	0.00% (0.00%-0.00%)	0.00% (0.00%-0.00%)
Anxiety	3.32% (2.68%-4.06%)	3.07% (2.47%-3.83%)	3.81% (3.31%-6.48%)	3.56% (3.10%-6.01%)
**Uterine and cervical factor**				
Endometriosis	0.12% (0.11%-0.15%)	0.11% (0.10%-0.15%)	0.15% (0.14%-0.15%)	0.14% (0.13%-0.14%)
Adenomyosis	0.26% (0.24%-0.28%)	0.24% (0.23%-0.27%)	0.26% (0.25%-0.27%)	0.24% (0.23%-0.25%)
**Fetal characteristics:**				
Twin pregnancy	3.19% (2.05%-5.17%)	2.94% (1.89%-4.73%)	1.55% (1.32%-1.85%)	1.45% (1.23%-1.72%)
**Total population attributable fraction**	**63.28%**	**65.72%**	**65.24%**	**70.50%**

*Min/Max is the minimum/maximum of the PAF_α_ of the RF (Equation 4) among the countries in Sub-Saharan Africa or South Asia.

**Figure 2:**
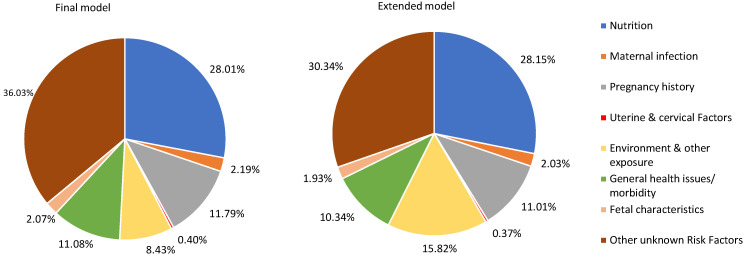
Population attributable fractions of risk factors for small-for-gestational age infants in 81 countries in the final and extended models.

**Figure 2** presents the percentage of SGA represented by these seven identified RF categories and unattributed RFs in the final and extended models of overall 81 LMICS. The final model’s Figure shows that maternal nutritional status RFs have the largest PAF globally (28.01%). The second highest contributing group is pregnancy history, with a PAF equal to 11.79%. This was followed by general health issues or morbidity (11.08%), environmental and other exposures during pregnancy (8.43%), maternal infection (2.19%), fetal characteristics (2.07%), and uterine or cervical factors (0.40%). Similarly, the Figure of the extended model shows that maternal nutritional status RFs have the greatest PAF globally (28.15%) and the second highest contributing group is environmental and other exposures during pregnancy (15.82%). This was followed by pregnancy history (11.01%), general health issues or morbidity (10.34%), maternal infection (2.03%), fetal characteristics (1.93%), and uterine or cervical factors (0.37%).

**Figure 3:**
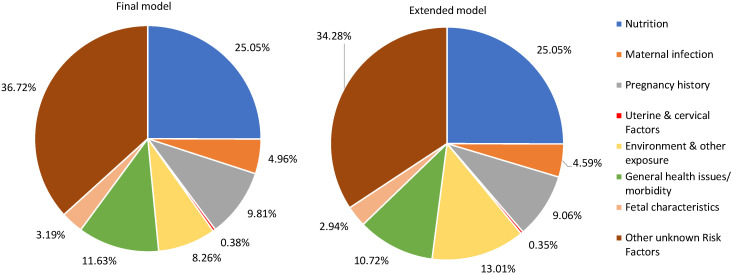
Sub-Saharan African region’s population attributable fractions for small-for-gestational age infants in the final and extended models.

**Figure 4:**
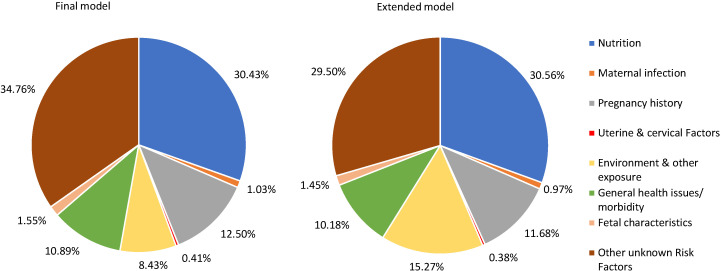
South-Asian Region’s population attributable fractions for small-for-gestational age infants in the final and extended models.

Since the burden of SGA is known to be high in SSA and SA, **Figure 3** and **Figure 4**, show the percentage of SGA represented by the seven identified categories and other unknown / unattributed RFs in the final and extended models of the two regions. In the SSA region (**Figure 3**), the top three RF groups in the final model are nutrition (25.05%), followed by general health issues or morbidity (11.63%), and pregnancy history (9.81%), while in the extended model for the region, the top three RF categories are nutrition (25.05%), followed by environment and other exposure (13.01%), and general health issues or morbidity (10.72%). As explained in the Methods section, environment and other exposure appeared as one of the top three RFs in the extended model since this category included one more RF, ie, indoor air pollution. Similarly, South-Asia’s final model (**Figure 4**) shows that nutrition (30.43%), pregnancy history (12.50%), and general health issues or morbidity (10.89%) are the top three RF categories, while the region’s extended model shows that nutrition (30.56%), environment and other exposure (15.27%) and pregnancy history (11.68%) are the top three RF categories.

RFs for SGA also tend to vary by country. **Table 4** presents country-specific SGA RF group profiles for South Sudan and Bangladesh to illustrate variation by country. For example, in the final model, maternal infection has a much higher PAF in South Sudan (5.41%) than in Bangladesh (0.89%). Although the PAFs of RFs for each country included in the analysis can be found in the **Online Supplementary Document** (Section S6), we chose these two countries since they represent different regions and have high burden of poor infant health outcomes.

**Table 4:** Comparison of population attributable fractions for small-for-gestational age infants in the final and extended models between Bangladesh and South Sudan

	Bangladesh		South Sudan	
	**Final model; Population attributable fraction**	**Extended model; Population attributable fraction**	**Final model; Population attributable fraction**	**Extended model; Population attributable fraction**
Nutrition	27.59%	27.41%	21.23%	21.35%
Maternal infection	0.89%	0.82%	5.41%	5.01%
Pregnancy history	15.42%	14.29%	12.51%	11.60%
Uterine & cervical Factors	0.39%	0.36%	0.38%	0.36%
Environment & other exposure	8.66%	16.89%	8.09%	17.20%
General health issues or morbidity	13.33%	12.36%	10.85%	10.05%
Fetal characteristics	1.78%	1.65%	3.37%	3.12%
Other unknown Risk Factors	31.95%	26.22%	38.15%	31.31%

## DISCUSSION

In both the final and extended models, the analysis identified 25 and 27 SGA RFs, which totaled to a PAF of 63.97% and 69.66%, respectively across the 81 LMICs. The extended model had two additional RFs (anemia and indoor air pollution) added to the existing 25 SGA RFs of the final model. About 36% of the PAF for SGA is unknown in the models, suggesting that there are other RFs that play a role in SGA. No single RF had a PAF greater than 10% of SGA, suggesting that SGA could be caused due to multiple factors and scaling up multiple interventions to reduce the associated RFs might have better outcome. Some RFs such as twin pregnancy do not have direct interventions to address the RF and the SGA birth. However, prevention of such RFs could be feasible option by indirect interventions such as improved fertility care, reducing the risk of twin pregnancies.

The relative importance of RFs for SGA birth varies by region and by country. Differences between regions and countries are important as a basis for prioritizing interventions. For example, prevention or treatment interventions on maternal infections like HIV will have a higher impact in HIV endemic countries than in other non-endemic countries. In countries where a high proportion of SGA is due to non-infectious RFs (vitamin D deficiency, early age at first pregnancy, short maternal height etc.), family planning, management of co-morbidities, and nutritional interventions throughout the life course may be more effective.

Infections are cited as one of the RFs for SGA and other analyses have shown that it accounts for less than 5%-15% of IUGR fetuses, a proxy for SGA [50,51]. The PAFs for maternal infections included in this analysis jointly contributed to less than 3% of SGA births. The association between infections like HIV-AIDS and SGA is complex since HIV is linked to other infections, social disruptions, and psychological distress. The evidence related to TORCH (Toxoplasmosis, Rubella, Cytomegalovirus, Herpes Simplex Virus) and other infections are described in detail at Section S3 in the **Online Supplementary Document**.

Some of the RFs included in our final model might not be easily amenable to interventions, such as short maternal stature that would require an intergenerational approach, or ambient air pollution that would require societal changes. Although prevalence and treatment of adenomyosis can be low in LMICs, we still included it in the model since systematic meta-analysis showed a statistically significant association and the paper was deemed to be of reasonable quality based on our quality checklist.

Our estimated PAF for short maternal height (<145 cm), is ie, 4.46% was less compared to the range estimated by Kozuki et al of 3.40%-24.3% [41]. Tobacco smoking during pregnancy was previously estimated as contributing to 10.3%-25.00% of SGA [52-54], but in our analysis the PAF was only 1.74%. Hypertension was cited as being responsible for 2.73% of SGA [54], but in our analysis the PAF was 6.02%. Our PAF estimate of vitamin D deficiency for SGA was 13.19%, which was much lower than a previous estimate of 22.60% [55]. The PAF of SGA birth related jointly to either short (<12 months) or long inter-pregnancy interval (≥36 months) was previously estimated to be 3.2% [56] and in our study short (<18 months) and long (≥60 months) birth interval contributed to 1.94% and 1.49% of SGA births, respectively. There are two reasons why the PAFs calculated in this paper tend to be lower. First, previous estimates were all from single-country studies conducted in non-LMICs, where the prevalence of the RFs is likely to be different from LMICs. Second our calculated individual PAFs adjust for all the other RFs included in the model, whereas the PAFs in previous analyses were calculated when only considering a single RF.

We did not include fetal genetic defects or placental abnormalities in order to focus on RFs that may be feasibly amenable to interventions in LMICs. Moreover, there was a lack of studies that explored SGA and genetic factors. Under uterine, placental, and cervical category, we explored RFs like endometriosis, adenomyosis, uterine malformations, early vaginal bleeding, placental previa etc. Only the former two RFs were included in our model and the remaining RFs were not included due to weak evidence to identify an association between such RF and SGA babies. Although there were interventions to address some of the RFs like medications for treating anxiety, and residential green and blue spaces to improve ambient air pollution, we could not adjust treatment effect of those interventions due to the lack of data on coverage of such interventions during pregnancy.

**Limitations:** Assessing gestational age in LMIC settings is challenging; ultrasound is generally not available and last menstrual period is used to calculate gestational age most pregnancies, which can be affected by poor maternal recall, lactational amenorrhea, variation in length of menstrual cycle, or injectable contraception [2]. SGA definitions, cut-offs, and reference standards varied across the studies which might affect classification of SGA births, affecting both SGA prevalence estimates and risk associations. Majority of the relationships between RFs and SGA were examined using observational studies. Although most of the estimated risk ratios adjusted for potential confounders in their analyses, there is still a possibility for residual confounding. There was a lack of country-specific prevalence data for some of the RFs such as heavy physical workload, vitamin D deficiency etc. We had to use modeled or regional prevalence to calculate the country-specific PAFs for countries with missing data. Therefore, we assigned a quality score to the prevalence of included RFs as well. Our analysis assumes that each woman has only one SGA RF and it is possible for various RFs to overlap. For example, pre-eclampsia could be more common in nulliparous women, environmental exposures could be concentrated more among women and families with lower socioeconomic status. However, as described at Section 3 in the **Online Supplementary Document**, majority of the studies we used for our analysis adjusted for multiple potential cofounding factors. Additionally, it is possible that the systematic and meta-analysis papers we used have heterogeneity, which could eventually affect our estimates. For example, cut-off points to define vitamin D deficiency varied across studies in the meta-analysis papers [43]. The RFs explored were before the COVID-19 pandemic occurred and it is possible that COVID might influence the results of our analyses.

**Strengths:** This is the first study to extensively explore all SGA RFs that are supported by existing evidence and estimate their singular contribution in the context of the others. Our study provides a ranking of RFs by their attributable fractions, which is helpful for prioritizing interventions. Other strengths of our analyses are that we examined the evidence for both observational and intervention studies and assessed the quality and consistency of each to reach a decision on what risk estimate to include.

## CONCLUSIONS

Despite the knowledge of SGA being associated with child survival and development, there is limited evidence supporting interventions that can effectively reduce the incidence of SGA birth. Additionally, there is a lack of high-quality, country-specific data on the prevalence of RFs for SGA infants which poses challenges for estimating its burden and developing effective interventions. The various types of RFs that play a role in SGA births highlight the importance of a multifaceted approach to tackle SGA. There are RFs for SGA births identified here that are amenable to interventions, some of which already exist. Some of the RFs such as nutritional deficiency, infection, morbidity, air pollution, family planning etc. need interventions before a woman enters pregnancy. Then, if primary prevention fails there are interventions that can be implemented during pregnancy; these include optimizing gestational weight gain, treating infection, detection and management of hypertension and pre-eclampsia etc. Depending on the types of RFs, interventions should be strategically targeted at either individual or household and/or community or policy level. For example, ambient air pollution can be tackled at a community or policy level to reduce SGA, while targeted micronutrient or balanced protein energy supplements at an individual level can be introduced in LMICs, where majority of pregnant women are undernourished. Similarly, there are known interventions such as IPTp that can potentially help reduce the prevalence of SGA, and should be implemented to achieve high coverage in pregnant women in countries where the prevalence of malaria is high. These results suggest new directions for the development and implementation of interventions to prevent SGA birth. Preventing poor fetal growth requires, addressing both the immediate and underlying determinants and hence require multiple interventions. There is also a need to research the mechanisms by which some of the RFs might hinder fetal growth.

## Additional material


Online Supplementary Document
