# Supplementary material for: A systematic review on estimating population attributable fraction for risk factors for small-for-gestational-age births in 81 low- and middle-income countries
Source: J Glob Health. 2022 Mar 26;12:04024. doi: 10.7189/jogh.12.04024 (PMC8942297; doi:10.7189/jogh.12.04024)
Supplement: Online Supplementary Document [file jogh-12-04024-s001.pdf]

Supplemental Material
A systematic review on estimating population attributable fraction for risk factors for small-for-gestational-age births in 81 low- and middle-income countries

Contents

Section 1: General Overview 6
Section 2: Summary of risk factors, interventions, and prevalence 9
Section 3: Sources of Risk Estimates and Quality 16
Maternal Infection 16
Malaria – Falciparum and Vivax 16
HIV 18
HCV infection 19
Cervicitis 19
Chlamydia 20
Asymptomatic bacteriuria 21
Periodontal disease 22
Helminth 22
Trichomonas vaginalis 23
Bacterial vaginosis 23
Influenza 24
Zika virus 25
Syphilis 26
Rubella 26
Measles 27
Toxoplasmosis 27
Herpes simplex virus 28
Environmental and Other Exposure during Pregnancy 28
Shift work 28
Heavy physical workload during pregnancy 29
Intimate partner violence 29
Stress during pregnancy 30
Secondhand smoking 31
Indoor air pollution 32

Ambient air pollution 34
Smoking 35
Alcohol consumption 36
Acrylamide exposure 37
Pregnancy History 37
Maternal age and parity 37
Birth interval 39
Previous history of SGA 40
Previously induced abortion 41
Maternal Nutritional Status 42
Maternal stature 42
Zinc deficiency 42
Anemia 43
Low pre-pregnancy BMI 44
High pre-pregnancy BMI 45
Low gestational weight gain 45
Vitamin A deficiency 46
Low calcium intake 46
Vitamin D deficiency 47
Maternal oxidant level 48
Low iodine intake 49
General Health Issues or Morbidity 49
Pre-eclampsia 49
Gestational diabetes mellitus 51
Hypertension 52
Polycystic ovary syndrome 53
Subclinical hypothyroidism 54
Anxiety disorder 55
Depression 55
Inflammatory bowel disease 56
Migraine 56
Uterine, Placental, and Cervical Factors 57
Endometriosis and adenomyosis 57

Early vaginal bleeding 58
Placenta previa 58
HPV vaccine 59
Fetal Characteristics 59
Fetal sex 60
Twin pregnancy 60
Vanishing twin 61
Section 4: Sources of Prevalence Estimates and Quality 62
Malaria 62
HIV 62
Chlamydia 62
Trichomonas vaginalis 63
Heavy physical workload during pregnancy 63
Secondhand smoking 63
Indoor air pollution 63
Ambient air particular matter 63
Maternal age and parity 64
Birth interval 64
Smoking during pregnancy 64
Alcohol consumption 64
Short maternal height 65
Anemia 65
Low pre-pregnancy BMI (BMI<18.5) 65
Low gestational weight gain (below IOM guidelines) 65
Vitamin D deficiency (<50 nmol) 66
Hypertension 66
Pre-eclampsia 66
Sub-clinical hypothyroidism 67
Anxiety 67
Inflammatory bowel disease 67
Endometriosis 68
Adenomyosis 68
Twin pregnancy 68

Section 5: Quality Assessment 69
Section 6: Global, Regional, and Country PAFs 71
Adjusted PAFs for low- and middle-income country 73
Afghanistan 73
Algeria 74
Angola 74
Azerbaijan 75
Bangladesh 76
Benin 76
Bhutan 77
Bolivia 78
Botswana 78
Burkina Faso 79
Burundi 80
Cambodia 80
Cameroon 81
Central African Republic 82
Chad 82
Comoros 83
Congo 84
Cote d'Ivoire 84
Democratic Republic of the Congo 85
Djibouti 86
Dominican Republic 86
Equatorial Guinea 87
Eritrea 88
Eswatini 88
Ethiopia 89
Gabon 90
Gambia 90
Ghana 91
Guatemala 92
Guinea 92

Guinea-Bissau 93
Guyana 94
Haiti 94
Honduras 95
India 96
Indonesia 96
Iraq 97
Jamaica 98
Kenya 98
Kyrgyzstan 99
Laos 100
Lesotho 100
Liberia 101
Madagascar 102
Malawi 102
Mali 103
Mauritania 104
Morocco 104
Mozambique 105
Myanmar 106
Namibia 106
Nepal 107
Nicaragua 108
Niger 108
Nigeria 109
North Korea 110
Pakistan 110
Panama 111
Papua New Guinea 112
Paraguay 112
Philippines 113
Rwanda 114
Senegal 114

Sierra Leone 115
Solomon Islands 116
Somalia 116
South Africa 117
South Sudan 118
Sudan 118
Suriname 119
Tajikistan 120
Tanzania 120
Timor-Leste 121
Togo 122
Turkmenistan 122
Uganda 123
Uzbekistan 124
Venezuela 124
Yemen 125
Zambia 126
Zimbabwe 126
Reference 127

Section 1: General Overview

The aim of this analysis is to estimate the contribution of individual risk factor to the overall risk of small-for-gestational-age (SGA) birth in 81 low- and middle-income countries (LMICs). This analysis focuses on risk factors for SGA birth, defined as birthweight below 10th percentile for gestational age. Since the definitions of SGA varied across the available studies, whenever possible, we gave priority to studies that defined SGA using INTERGROWTH 21st to maintain consistency. [1]

Once the risk factors were identified, computer-based searching and consultations with research team members were employed to find appropriate sources for the corresponding risk. When possible, published meta-analyses or Cochrane review papers that had the most recent data published were used to estimate risk. Additionally, priority was given to meta-analyses or Cochrane review papers that analyzed studies primarily conducted in LMICs. If estimates between a risk factor (RF) and SGA was reported as odds ratio, the estimates were converted to relative risks to calculate population attributable fraction for the respective RF. [2] When published research paper was available, we also included intervention trials to validate risk

association between the RF and SGA and have provided description of intervention trials that addressed SGA or the RF associated with SGA under the respective risk factor in Section 3.

To assess the quality of the evidence for each risk factor, PRISMA and GRADE checklist were used as reference to create two checklists to separately grade the quality of published research papers either on 1) risk factors of SGA or 2) interventions of the RF that addressed SGA. [3 4] Please refer to the quality assessment section to get detailed information on the two checklists. There were three categories of quality assigned to the research papers: high, medium, or low. In general, high quality evidence refers to systematic review and meta-analysis with a large number of randomized design or observational studies, were primarily conducted in LMICs, clearly defined the variables and the outcome, adjusted for sufficient potential confounders, had low heterogeneity, and estimates had sufficiently large magnitude measures of association. Medium quality evidence refers to systematic review and meta-analysis with a limited number of randomized design or observation studies, were primarily conducted in non-LMICs, did not clearly define the variables or the outcome, adjusted for insufficient potential confounders, and had medium heterogeneity. Low quality evidence refers to meta-analysis or single studies with low number of randomized design or observational studies, were only conducted in non-LMICs, did not clearly define the variables and the outcome, did not adjust for any potential confounders, had high heterogeneity, and estimates had small magnitude measures of association.

After grading observational studies of the RF and the interventional trials that addressed SGA based on the two checklists, we also estimated an "overall quality" of the risk estimate that took into account of both quality scores for each of the potential RF. In this combined overall quality assessment, there are six tiers of quality: high, medium-high, medium, medium-low, low, and very low. The complete coding rule for the overall quality is also presented in the quality assessment section (Section 5). If the overall quality of the RF was very low, we excluded the risk factor and did not provide a risk estimate. For all the other RFs with higher quality, we provided risk estimates with a 95% confidence interval (C.I.). The point estimate for the risk was from observational data for most of the risk factors except those where the risk factor is only due to nutrient deficiency, or there were inconclusive risk estimates from individual observational studies. For risk factors that are only due to nutrient deficiency, when rigorous randomized control trial that explored the impact of interventions on mitigating the risk factor was available, we took the inverse of the treatment effect to estimate the relative risks. When evidence from individual observational studies was inconsistent, we also used the inverse of the treatment effect from randomized control trial to estimate the relative risks of the RF.

Some of the identified risk factors were excluded from our final analytical model due to one of the following reasons: a) adequate evidence of no association between risk factor and SGA; or b) insufficient evidence of a relationship between the risk factor and SGA; or c) the risk factor is not independent of other SGA risk factors, i.e., the risk factor is either an intermediate outcome between another risk factor and SGA.

For anemia and indoor air pollution, both had evidence with high variability among systematic reviews and multiple single studies in LMICs. With regards to anemia, a 2016 review of data from LMIC found no association between anemia and SGA, while a later meta-analysis that included data from high-income countries did. Please refer to source of risk estimate section to see the description of existing evidence for anemia and indoor air pollution. Given that both tend to have a high prevalence in LMICs, we decided to construct an additional model (extended model) and include them in the model to make sure we capture all the potential association with SGA.

For the prevalence data, priority was given to country specific estimates from DHS surveys, or those developed by groups such as Institute for Health Metrics and Evaluation (IHME), Joint United Nations Programme on HIV/AIDS (UNAIDS), or the World Health Organization (WHO). When country-specific data were unavailable, regional data were used. In some instances, the average of each region was computed from available country-specific data from systematic reviews. In three instances, global data was calculated from limited numbers of country-specific data. We used Lives Saved Tool (LiST) database to get country level estimates of SGA rates for 2020.

Section 2: Summary of risk factors, interventions, and prevalence

In this section, the evidence available on all the risk factors for SGA is summarized in supplemental Tables 1a and 1b. We categorized risk factors into one of the seven categories: maternal infection; environment and other exposure during pregnancy; pregnancy history; maternal nutritional status; general health issues or morbidity; uterine, placental, and cervical factors; fetal characteristics. Supplemental Table 1a includes summary of 25 RFs that were included in our final analytical model, plus two additional RFs (anemia and indoor air pollution) that were included in the extended model. Although we have separate risk estimates for birth interval of < 18 months and 18- <24 months, we regarded both of them as one RF, i.e., short birth interval. Supplemental Table 1b provides summary of the 36 RFs that were excluded in the final model and the extended model. Agreement on effects pertains to having similar direction and strength of association between research papers on 1) RFs and 2) intervention trail for each of the potential RF. Supplemental Table 1c. provides weighted prevalence of the 27 risk factors by birth, globally and by six WHO regions.

Supplemental Table 1a. Summary of included risk factors

Risk Factor	Relative Risk (95% Confidence Interval)	Risk Score	Risk Quality	Intervention[d]	Intervention Quality	Intervention Score	Overall Quality	Agreement on Effects	Prevalence	Prevalence Quality
**Maternal Infection**										
Plasmodia falciparum malaria	1.10(1.02-1.18)[5]	10.5	High	Quinine	Medium	12.5	Medium-High	Yes	Country specific[c]	Medium
HIV	1.64(1.29-2.09)[11]	11	High	NA	NA	NA	Medium	NA	Country specific	High
Chlamydia	1.13(1.05-1.24)[15]	9	Medium	Erythromycin drug	Low	4	Medium-Low	No	Country specific	High
Trichomonas vaginalis	1.51(1.32-1.73)[26]	9	Medium	NA	NA	NA	Low	NA	Country specific	High
**Environmental and other Exposure**										
Heavy Physical workload	1.07(1.00-1.13)[44]	10.5	High	NA	NA	NA	Medium	NA	Regional & country level data	Medium-low
Secondhand smoking	1.03(1.00-1.07)[50]	9	Medium	Smoke-free legislation	Low	6.5	Medium-Low	No	Country specific	Medium

Indoor air pollution[a]	1.23(1.02-1.49)[52]	11	High	Vented stoves	Low	8.5	Medium	No	NA	NA
Ambient air pollution	1.12(1.08-1.16)[58]	11	High	Residential green and blue spaces	Low	8.5	Medium	Yes	Country specific	Medium
Smoking	1.86(1.81-1.91)[50]	10	Medium	NA	NA	NA	Low	NA	Country specific	Medium
Alcohol consumption	1.17(1.03-1.32)[62]	11	Medium	NA	NA	NA	Low	NA	Country specific	Medium-high
**Pregnancy History**										
Young maternal age < 18 & Primiparity	1.70(1.32-1.53)[64]	10.5	High	NA	NA	NA	Medium	NA	Country specific	High
Young maternal age 18-35 and Primiparity	1.32(1.25-1.39)[64]	10.5	High	NA	NA	NA	Medium	NA	Country specific	High
Birth interval of < 18 months	1.44(1.27-1.63)[65]	10.5	High	NA	NA	NA	Medium	NA	Country specific	High
Birth interval of 18- <24 months	1.20(1.03-1.40)[65]	10.5	High	NA	NA	NA	Medium	NA	Country specific	High
Birth interval of over 60 months	1.15(1.05-1.25)[65]	10.5	High	NA	NA	NA	Medium	NA	Country specific	High
**Maternal Nutritional Status**										
Maternal Stature	2.03(1.76-2.35)[70]	9.5	Medium	NA	NA	NA	Low	NA	Country specific	Medium
Anemia[a]	1.07(1.00-1.05)[73]	10	Medium	Iron folic acid	High	13.5	High	No	NA	NA
Low pre-pregnancy Body Mass Index	1.21(1.12-1.31)[77]	10	Medium	Balanced protein energy	High	14	High	Yes	Country specific	High

Low gestation weight gain	1.36(1.31-1.43)[83]	10.5	High	NA	NA	NA	Medium	NA	Regional & country level data	Low
Vitamin D deficiency[b]	1.39(1.01-1.92)[90]	10.5	High	Vitamin D supplementation	Medium	11	Medium-High	Yes	Global & country level data	Low
**General Health Issues/Morbidity**										
Pre-eclampsia	1.63(1.55-1.71)[99]	9	Medium	Magnesium Sulfate	High	15.5	High	NA	Regional[c]	low
Hypertension	1.38(Not reported)[99]	9	Medium	Antihypertensive agents	High	15	High	No	Country specific	High
Subclinical hypothyroidism	1.23(1.04-1.46)[109]	11	High	NA	N/A	NA	Medium	NA	Global	Low
Anxiety Disorder	1.35(1.17-1.54)[111]	10	Medium	Benzodiazepine	Low	9	Medium-Low	No	Global & country level data	Low
Inflammatory Bowel Disease	1.36(1.16-1.60)[114]	9.5	Medium	NA	NA	NA	Low	NA	Regional	Low
**Uterine, Placental, and Cervical Factors**										
Endometriosis	1.25(1.01-1.54)[117]	10.5	Medium	NA	NA	NA	Low	NA	Country specific	High
Adenomyosis	2.55(1.58-3.78)[117]	8.5	Medium	NA	NA	NA	Low	NA	Global	Low
**Fetal Characteristics**										
Twin pregnancy	3.98(Not reported)[127]	10	Medium	NA	NA	NA	Low	NA	Country specific	Medium

Note:
NA: Not applicable
a: Risk factors included in the extended model.
b: Risk factor that is only due to nutrient deficiency, therefore relative risk calculated from the inverse of the treatment effect.
c: Prevalence was adjusted for treatment effects.
d: Intervention trials of the risk factor that included SGA as an outcome.

Supplemental Table 1b. Summary of excluded risk factors

Risk Factor	Relative Risk (95% Confidence Interval)	Risk Score	Risk Quality	Intervention[c]	Intervention Score	Intervention Quality	Overall Quality	Agreement on Effects	Reason for Exclusion
**Maternal Infection**									
Plasmodium vivax malaria	Exclude	7.5	Low	NA	NA	NA	Very Low	NA	Insufficient[a]
Hepatitis C- Virus	1.41(0.78-2.57)[13]	9.5	Medium	NA	NA	NA	Low	NA	Sufficient
Cervicitis	Exclude	5	Low	NA	NA	NA	Very Low	NA	Insufficient[a]
Asymptomatic bacteriuria	0.9(0.5-1.5)[17]	4.5	Low	Antibiotics	8	Low	Low	Yes	Sufficient
Periodontal disease[a]	1.03(0.86-1.23)[22]	4	Low	Scaling and root panning or oral hygiene instructions	10	Medium	Medium	Yes	Sufficient
Helminth	Exclude	5	Low	NA	NA	NA	Very Low	NA	Insufficient[a]
Bacterial vaginosis[a]	0.93(0.78-1.10)[29]	5	Low	Antibiotics	7.5	Low	Low	No	sufficient
Influenza	1.0(0.8-1.3)[30]	8	Medium	Influenza vaccine	12	Medium	Medium	Yes	sufficient
Zika virus	Exclude	4	Low	NA	NA	NA	Very Low	NA	Insufficient[a]
Syphilis	Exclude	3.5	Low	NA	NA	NA	Very Low	NA	Insufficient[a]
Rubella	Exclude	1.5	Low	NA	NA	NA	Very Low	NA	Insufficient[a]
Measles	Exclude	4	Low	NA	NA	NA	Very Low	NA	Insufficient[a]
Toxoplasmosis	Exclude	3	Low	NA	NA	NA	Very Low	NA	Insufficient[a]
Herpes	Exclude	2.5	Low	NA	NA	NA	Very Low	NA	Insufficient[a]
**Environmental and other Exposure during Pregnancy**									
Shift work	1.01(0.92-1.10)[43]	8	Medium	NA	NA	NA	Low	NA	sufficient
Intimate partner violence	1.6(0.61-4.1)[45]	10	Medium	NA	NA	NA	Low	NA	sufficient
Stress during pregnancy	Exclude	5.5	Low	NA	NA	NA	Very Low	NA	Insufficient[a]
Acrylamide exposure	1.11(1.02-1.20)[62]	8.5	Medium	NA	NA	NA	Low	NA	Insufficient[b]
**Pregnancy History**									
Maternal age 18-35 and multiparous	0.93(0.88-0.99)[64]	10.5	High	NA	NA	NA	Medium	NA	Sufficient
Maternal age >35 & parity >3	0.98(0.87-1.09)[64]	10.5	High	NA	NA	NA	Medium	NA	Sufficient
Previous history of Small-for-Gestational Age	Exclude	7.5	Low	NA	NA	NA	Very Low	NA	Insufficient[a]
Previously induced Abortion	Exclude	7.5	Low	NA	NA	NA	Very Low	NA	Insufficient[b]

**Maternal Nutritional Status**									
Zinc deficiency[b]	0.98(0.89-1.09)[71]	NA	NA	Zinc supplement	13.5	High	High	NA	Sufficient
High pre-pregnancy Body Mass Index	0.71(0.66-0.76)[78]	9.5	Medium	NA	NA	NA	Low	NA	Sufficient
Vitamin A deficiency[b]	1.12(0.85-1.47)[84]	NA	NA	Vitamin A+carotenoids	11	Medium	Medium	NA	Sufficient
Low calcium intake[b]	1.18(0.83-1.67)[85]	NA	NA	Calcium supplement	13	Medium	Medium	NA	Sufficient
Maternal oxidant levels[b]	1.02(0.94-1.11)[93]	6.5	Low	Vitamin E and C	13	Medium	Medium	Yes	Sufficient
Low Iodine Intake	Exclude	4.5	Low	NA	NA	NA	Very Low	NA	Insufficient[a]
**General Health Issues/Morbidity**									
Gestational diabetes mellitus[a]	0.91(0.68-1.23)[104]	NA	NA	Diet modification, glucose monitoring, or insulin	9.5	Low	Low	NA	Sufficient
Polycystic ovary syndrome	1.45(0.96-2.20)[108]	8	Medium	NA	NA	NA	Low	NA	Sufficient
Depression	Exclude	7.5	Low	NA	NA	NA	Very Low	NA	Insufficient[a]
Migraine	1.06(0.99-1.14)[115]	9.5	Medium	NA	NA	NA	Low	NA	Sufficient
**Uterine, Placental, and Cervical Factors**									
Early vaginal bleeding	Exclude	3.5	Low	NA	NA	NA	Very Low	NA	Insufficient[a]
Placenta previa	Exclude	7.5	Low	NA	NA	NA	Very Low	NA	Insufficient[a]
Human Papilloma Virus vaccine	0.96(0.86-1.07)[123]	10.5	High	NA	NA	NA	Medium	NA	Sufficient
**Fetal Characteristics**									
Fetal sex	Exclude	5	Low	NA	NA	NA	Very Low	NA	Insufficient[a]
Vanishing twin	Exclude	4	Low	NA	NA	NA	Very Low	NA	Insufficient[a]

Notes:

NA: not applicable

a: Risk factor with no observational study or inconclusive evidence from individual observational studies, the relative risk estimate is the inverse of the treatment effect from randomized control trial.

b: Risk factor that is only due to nutrient deficiency, the relative risk estimate is the inverse of the treatment effect from randomized control trial.

c: Intervention trials that included SGA as an outcome.
Insufficient [a]: Insufficient evidence of risk relationship due to limited availability of evidence.
Insufficient [b]: Insufficient evidence of risk relationship due to limited availability of evidence and lack of evidence in LMICs
Sufficient: Sufficient evidence of no risk relationship between risk factor and SGA
Dependent: the risk relationship between the risk factor and SGA is already captured by other risk factors included in the model

Supplemental Table 1c. Weighted prevalence of included risk factors by World Health Organization regions

	**Global**	African region **(AFRO)**	Region of the Americas **(AMRO)**	Eastern Mediterranean Region **(EMRO)**	European Region **(EURO)**	South-East Asia Region **(SEARO)**	Western Pacific Region **(WPRO)**
Malaria	9.2%	20.9%	0.1%	1.1%	0.8%	0.4%	1.0%
HIV	1.5%	3.3%	0.8%	0.0%	0.2%	0.1%	0.2%
Chlamydia	1.8%	2.2%	2.8%	1.8%	3.3%	1.0%	2.3%
Trichomonas vaginalis	4.3%	6.4%	3.8%	2.6%	3.8%	2.7%	2.4%
Vitamin D Deficiency	60.4%	51.0%	54.0%	61.7%	54.0%	73.3%	38.1%
Maternal age <18 & Primiparity	4.6%	5.8%	7.7%	2.7%	1.7%	4.2%	2.9%
Maternal age 18-35 & Primiparity	25.0%	16.4%	22.5%	23.1%	35.4%	34.2%	30.6%
Birth interval < 18 months	6.7%	5.7%	6.3%	10.1%	6.8%	6.2%	5.9%
Birth interval 18- <24 months	10.6%	10.9%	9.0%	12.6%	9.7%	9.1%	9.2%
Birth interval 60 months or Greater	14.3%	8.7%	24.8%	12.6%	26.3%	18.0%	29.3%
Alcohol consumption	1.6%	2.1%	4.7%	0.0%	11.1%	0.4%	6.1%
Short height	6.0%	2.5%	5.9%	4.5%	1.5%	10.9%	7.2%
Low pre-pregnancy Body Mass Index	16.4%	12.1%	5.6%	12.1%	5.3%	24.4%	15.6%
Low gestational weight gain	41.1%	54.1%	23.0%	32.0%	28.8%	31.0%	30.5%
Hypertension	26.9%	28.2%	19.7%	28.5%	24.3%	24.8%	22.0%
Preeclampsia	3.1%	4.0%	2.3%	1.2%	3.8%	2.7%	4.2%
Subclinical hypothyroidism	3.0%	3.0%	3.0%	3.0%	3.0%	3.0%	3.0%
Inflammatory Bowel Disease	0.0%	0.0%	0.3%	0.1%	0.4%	0.0%	0.0%
Endometriosis	0.8%	0.7%	0.5%	0.9%	1.4%	0.9%	0.7%
Adenomyosis	0.3%	0.3%	0.3%	0.3%	0.3%	0.3%	0.3%
Twin pregnancy	1.2%	1.7%	0.9%	0.9%	0.9%	0.8%	0.7%

Anxiety	16.7%	15.2%	15.9%	16.9%	15.9%	17.8%	18.1%
Heavy physical workload	46.2%	64.8%	54.8%	22.0%	49.6%	30.3%	62.2%
Smoking during pregnancy	3.0%	2.3%	4.6%	3.4%	4.8%	3.0%	5.5%
Ambient air pollution	56.9%	47.0%	43.0%	63.3%	43.5%	68.1%	38.4%
Secondhand smoking	17.2%	11.0%	16.8%	18.9%	24.1%	20.2%	35.4%
Anemia	46.3%	46.7%	34.8%	42.1%	28.0%	49.3%	36.1%
Indoor air pollution	68.1%	85.8%	53.7%	44.8%	10.0%	61.3%	52.5%

Section 3: Sources of Risk Estimates and Quality

This section provides a brief description on the source of the published research articles that calculated risk estimates either on 1) risk factors of SGA or 2) interventions that addressed SGA or RF associated with SGA. We also assigned overall quality for each RF based on the quality of observational studies of the RF and, if available, the intervention trials that included SGA as an outcome. When multiple risk estimates were available, we chose the best estimate based on publication year, study design, and study setting. When possible, published meta-analyses or Cochrane review papers that had the most recent data published were used to estimate risk. Additionally, priority was given to meta-analyses or Cochrane review papers that analyzed studies primarily conducted in LMICs.

Maternal Infection

Malaria – Falciparum and Vivax

Plasmodium falciparum malaria

Risk: The primary estimate of risk of malaria on SGA babies is based on a paper by Saito, M. et al., a systematic review and meta-analysis of 16 studies (n= 4503). [5] The observational and RCT studies were conducted in Asia and Africa and were published between 2000-2018. Studies were included if plasmodium falciparum parasitemia was confirmed by microscopy before treatment. Parasitemia was measured in $\log_{10}$/μL and SGA was defined as birthweight of < 10th percentile. The analysis was adjusted for age, height, BMI, parity or gravidity, HIV status. Higher baseline parasitemia before treatment was statistically associated with a higher risk of SGA babies (aOR= 1.14 per 10-fold increase, 95% C.I.= 1.03-1.26). [5] Using the prevalence from the study, we converted the aOR to adjusted RR=1.10 (1.02-1.18). [5]

Another prospective study by Moore et al., enrolled 50,060 pregnant women in Thailand-Myanmar border between 1986–2015. [6] The odds of SGA babies increased linearly with the number of falciparum malaria episodes in pregnancy. The odds of SGA increased by 1.13 times with each episode of infection with falciparum malaria (95% C.I.: 1.09-1.17).

Another study was conducted HIV-infected pregnant women living in Tanzania, who were enrolled in an ongoing randomized, double-blind, placebo-controlled clinical trial of supplementation with vitamin A or multivitamins. The study included 822 women and was conducted from 1995 to July 1997. Multivariate-adjusted odds ratio from a logistic regression model controlling for multivitamin and vitamin A supplement group, gestational age at baseline, maternal weight, low vitamin E concentration, candidiasis, malaria, infant sex, and HIV status at birth. P. falciparum infection was not statistically associated with SGA babies (aOR=1.79; 95% C.I.=1.06, 3.02). [7]

Plasmodium vivax malaria

A retrospective study by Dombrowski et. al. was conducted between 2006 and 2014 in Brazil with a sample size of 16,444 live births. [8] The study performed multivariate logistic regression model adjusting for maternal age, gravidity, and education status. The study found that being

infected with malaria during pregnancy was statistically associated with an increased odds of SGA babies (aOR= 1.23, 95% C.I.= 1.05–1.45, p= 0.013). [8] The prevalence of malaria during pregnancy in this study was 8.9% and we used this value to calculate adjusted risk estimates aRR=1.21 (1.05,1.39). The study found that although P. vivax infection was statistically associated with an increased odds of SGA babies (aOR= 1.24, 95% C.I.= 1.02–1.52, p= 0.035). P. falciparum infection was not statistically associated with SGA babies (aOR= 1.21, 95%, CI= 0.89–1.64, p = 0.224). Although not statistically significant, it also indicates that there were fewer cases of P. falciparum than vivax.

Another prospective study by Moore et al., enrolled 50,060 pregnant women in Thailand-Myanmar border between 1986–2015. [6] The odds of SGA babies increased linearly with the number of vivax malaria episodes in pregnancy. The odds of SGA also increased by 1.27-fold for each episode of vivax malaria (95% C.I.: 1.21-1.33).

<u>Studies that did not categorize Plasmodium falciparum or vivax:</u>
In another meta-analysis study by Cates et. al.,14,633 singleton live birth pregnancies were included from 13 studies that were conducted from 1996 to 2015 in Africa and Western Pacific regions (4 from Kenya, 2 from Malawi, 2 from Papua New Guinea, Ghana, Benin, Burkina Faso, Tanzania, Democratic republic of Congo). [9] The paper includes RCT and cohort study designs. 9 studies used ultrasound-dated gestational age to assess SGA. SGA definition is birth weight less than the 10th percentile of the INTERGROWTH-21st reference. Some women were enrolled after 24 weeks gestation, which reduced the accuracy of ultrasound among these pregnancies and potentially underestimating gestational age in some SGA babies. Malaria infection defined as a positive LM (Light Microscopy), RDT (Rapid Diagnostic Test), or PCR (Polymerase Chain Reaction). Malaria at enrollment was not statistically associated with SGA babies (adjusted Odds ratio= 1.16, 95%, CI= 0.96–1.41). [9] Enrollment defined as the first antenatal care visit. Malaria at delivery was not statistically associated with SGA babies (adjusted Odds ratio= 1.09, 95%, CI= 0.88–1.35). [9] Although not statistically significant, the point estimate is similar to the Saito, M. et al.'s estimate.
Note: Other RCT/observational studies conducted in Africa (Malawi, Uganda) found mixed results with regards to malaria infection and SGA birth. One study conducted in Malawi found a non-significant association (RCT; N=1451), while another study in Malawi (observational study; N=321) found a significant risk of malaria and SGA, but only if women had malaria at the time of delivery. Another study conducted in Uganda found a non-significant association between falciparum malaria and SGA birth (cohort study; N=282).

Intervention:
<u>Plasmodium falciparum malaria</u>
A systematic review and meta-analysis was conducted to assess the efficacy of artemisinin based and quinine-based treatments for patent microscopic uncomplicated falciparum malaria infection in pregnancy was conducted. The study used data from 16 studies with 4440 women. Quinine was statistically associated with reduced odds of SGA babies compared to artemether-lumefantrine (aOR 0.22; 95% C.I. 0.05–0.92, p= 0.04). [5] The estimates were adjusted for potential confounders such as age, height, BMI, parity or gravidity, HIV status. Other

medications such as artesunate monotherapy, artesunate-amodiaquine, artesunate-mefloquine, artesunate-sulfadoxine-pyrimethamine, dihydroartemisinin-piperaquine, quinine with clindamycin used to treat malaria was not statistically associated with SGA compared to artemether-lumefantrine.
Based on the same systematic review and meta-analysis study, in both univariable and multivariable analyses, different antimalarial treatments (artesunate with atovaquone-proguanil, artesunate monotherapy, artesunate-amodiaquine, artesunate-mefloquine, artesunate-sulfadoxine-pyrimethamine, dihydroartemisinin-piperaquine, quinine monotherapy, quinine with clindamycin) were not statistically associated with SGA when compared with artemether-lumefantrine. [5]

A prospective cohort study of pregnant women by Augusto et. al. conducted in 3 African countries with a sample size of 1915 live births reported that women who were exposed to quinine had 60% higher prevalence of having an SGA baby compared to those who received artemisinin-based combination therapy (Prevalence Ratio= 8.60; 95% C.I.= 1.29–57.6, p-value= 0.027).[10] There was a heterogeneity of 0% (p value= 0.41).

Overall quality of risk estimate: Medium for falciparum malaria. The risk relationship between falciparum malaria and SGA was based on meta-analysis of observation studies and RCT studies conducted in African and Asian regions. The risk estimates were adjusted for confounders.
Very low for vivax malaria. Two low quality single studies in LMICs showed some increased risk of SGA.

Falciparum Malaria - MODEL INCLUSION: RR=1.10 (1·02-1.18). Evidence based on observational studies and randomized controlled trials. Quinine-based treatment is effective at reducing risk of SGA.

Vivax Malaria - MODEL INCLUSION: Not included. Insufficient evidence to demonstrate association with SGA due to limited availability of evidence.

HIV

Risk: The estimate of risk of HIV on SGA birth is from a systematic review and meta-analysis paper that had 7 studies (4 prospective cohort studies, 3 retrospective cohort studies) and included 16,683 women. [11] The studies were conducted in South Africa, India, Kenya, Rwanda, US, Zimbabwe, and Tanzania (between 1994 to 2012). SGA was defined as birthweight <10th centile. Maternal HIV infection is statistically associated with an increased risk of SGA baby (RR=1.64; 95% C.I.= 1.29–2.09) (I^2= 74.9%, p= 0.001). [11] Correction for confounders did not affect the significance of these findings.

Intervention: A systematic review and meta-analysis of 11 studies was published between 1980-2016. [12] However, in the paper, only 2 studies were used to analyze association between antiretroviral therapy and SGA (Botswana and Tanzania; N= 4152). Small for gestational age was an outcome in two studies and the overall risk of SGA (RR= 1.13, 95% C.I.= 0·94–1·35) did not differ significantly between women who began ART before conception and those who did so after conception. [12] However, the treatment effects were at conception or before, so it is not a

good estimate. Additionally, there was no comparison done between those who received that treatment versus the untreated group.

Overall quality of risk estimate: Medium. Systematic and meta-analysis with a large sample size based on observational studies conducted in LMIC and non-LMIC counties.

MODEL INCLUSION: RR= 1.64 (1.29–2.09). Evidence based on observational data.

Hepatitis C- Virus infection

Risk: The risk estimate is based on a systematic review published in 2016. [13] The review evaluated the association between maternal Hepatitis C- Virus (HCV) infection and intrauterine fetal growth disturbance (IUGR). HCV diagnosis was based on ICD code, anti-HCV (ELISA), or HCV-RNA. IUGR was defined as birth weight <10th percentile for gestational age based on population-based growth standards. Seven studies, with 4,185,414 participants, conducted between 2007-2014 were included. Five studies were conducted in USA, one in India and one in Pakistan. Different studies adjusted different covariates such as maternal age, parity, maternal smoking/alcohol abuse, drug abuse, coinfected with HBV/HIV, rate pregnancy complication (pre-eclampsia). Compared to non-HCV infected pregnant women, HCV infected pregnant women had an increased risk of having an infant born IUGR (OR=1.53 [1.40-1.68]). [13] The prevalence of HCV (+) among participants included in the review was 0.12%. The subgroup analysis conducted by location / region concluded that the two studies conducted in Asia (OR=1.41 [0.78-2.57]) did not find significant association between HCV and IUGR. [13] The quality of the study was assessed using Newcastle-Ottawa Scale. Five out of seven studies received a score 7 or higher, considered as high quality. Based on the subgroup analysis, the risk relationship was only observed in USA population.

Overall quality of risk estimate: Low. In the review, IUGR definition was similar to the SGA definition. Case-control studies and retrospective cohort studies were included in the review.

MODEL INCLUSION: Not included. Sufficient evidence to show no association between HCV infection and SGA in LMICs.

Cervicitis

The estimate of risk of cervicitis is from a retrospective cohort study conducted in the U.S., which enrolled 141,035 women and infants born from 1996-2002. [14] SGA was defined as birth weight below the 5th percentile for gestational age. Exposure (maternal infections) was ascertained using ICD-9 codes indicating a diagnosis of one or more of the following conditions during pregnancy: trichomoniasis, gonorrhea, Chlamydia trachomatis/ non-gonococcal urethritis (referred to throughout the rest of the article as chlamydia), vulvovaginal candidiasis, urinary tract infection, vaginitis, cervicitis, upper reproductive tract infections, and unspecified ‘‘infections of the genitourinary tract during pregnancy. [14]

After controlling for demographic covariates and maternal medical conditions in the regression model, maternal genitourinary infection (GUI) was not significantly associated with SGA (odds ratio=1.03; 95% C.I.=0.99-1.08 p=0.143). [14] When the study examined specific diagnoses on infection made at any time during pregnancy, only cervicitis (adjusted OR=1.14; 95% C.I.=1.02-1.27 p=0.019) and unspecified cervicitis/vaginitis (adjusted OR= 1.20; 95% C.I.=1.02-1.27 p=0.049) were statistically significant. [14] The prevalence of cervicitis during pregnancy in this study was 3.5% and we used this value to calculate adjusted risk estimates aRR=1.19 (1.02,1.26). Second trimester genitourinary infections had a statistically significant, though, weak association with SGA (adjusted OR=1.08, 95% C.I.=1.03-1.15, p=0.005). [14]

Overall quality of risk estimate: Very Low. The results were from a single study conducted in a non-LMIC country. The study only relied on billing records to identify women with gestational infection due to difficulty in accessing information about maternal infections and the authors indicated that ICD-9 codes often lack specificity. Women with subclinical infections would not have received any diagnosis unless they were screened, and widespread screening of pregnant women for infections other than chlamydia and gonorrhea generally is not conducted. So, it is likely that the actual rate of infection was higher than reported in the billing records. If so, then the ‘‘uninfected’’ women in our cohort would have included some women who had undiagnosed infections. This could have biased our results toward a null finding.

MODEL INCLUSION: Not included. Insufficient evidence to demonstrate association with SGA due to limited availability of evidence.

Chlamydia

Risk: The risk of Chlamydia infection on SGA births is from a 2018 systematic review and meta-analysis that included 25 studies, but the paper used 7 studies to explore SGA (2 from US, and 1 from Finland, Australia, India, Netherlands, Norway). [15] The sample size of the study was 362,727 (prospective and retrospective studies). Estimates of the prevalence of chlamydia in pregnant women in high income countries range from 3 to 14%. Chlamydia infection in pregnancy was associated with an increased risk of SGA (OR= 1.14; 95% C.I. = 1.05 - 1.25; p < 0.01). [15] The meta-analysis included 21,801 pregnant women with chlamydia, the total number of subjects was 614,892 and the estimated prevalence across the 25 studies was 3·54%. The information was used to convert the odds ratio to an estimated RR= 1.13 (1.05-1.24).

Intervention: A retrospective study was conducted in the U.S. with a sample size of 323 and published in 2018. [16] The frequencies of SGA infants were considerably lower in group 1 (successfully treated for Chlamydia) when compared with group 2 (remained positive for chlamydia infection during pregnancy), OR= 0.45 (95% C.I.= 0.23-0.88). [16] Erythromycin drug was given to treat chlamydia infection. The paper did not mention about adjusting for any variables. There is limited availability of evidence.

Overall quality of risk estimate: Low. There was evidence from systematic review and meta-analysis of observational studies with a large sample size. Only one study was conducted in a

LMIC country and others were all conducted in non-LMIC countries. SGA was not clearly defined. There were differences in laboratory methodologies to diagnose the infection across the studies. Additionally, only some of the included studies controlled for potential confounders. Although there a significant treatment effect it was a single observational study conducted in 1 non-LMIC country.

MODEL INCLUSION: RR= 1.13 (1.05-1.24). Evidence based on observational data.

Asymptomatic bacteriuria

Risk: A prospective study was conducted in the Netherlands between 2011 and 2013, which enrolled a total of 4242 women.[17] Small for gestational age was defined as <10th percentile. The analysis adjusted for smoking, low education, conception through in-vitro fertilization or intracytoplasmic sperm injection, and pre-existent hypertension. Asymptomatic bacteriuria occurs in 2–10% of pregnant women. The study used a single dipslide test to detect asymptomatic bacteriuria. The dipslide has 98.0% sensitivity and 99.6% specificity to detect asymptomatic bacteriuria in pregnancy and has been shown to be a good alternative for ordinary culture. SGA infants did not statistically differ between asymptomatic bacteriuria-positive women who were untreated or received placebo versus asymptomatic bacteriuria-negative women (aOR= 0.9; 95% C.I.= 0.5–1·5). [17]

Intervention: The common treatment for asymptomatic bacteriuria was antibiotic treatment. According to the 2019 Cochrane review, compared to no treatment, antibiotics was effective at reducing persistent bacteriuria (RR=0.3 [0.18-0,53]; 4 RCTs; 596 participants; I^2=76.04%)[18] However, the Cochrane review did not assess SGA. A recent unblinded cluster-randomized control trials in Sylhet, Bangladesh found that the risk of SGA did not differ significantly between control group and intervention group (RR=0.86 [0.74-1.01]). [19] Pregnant women in the intervention group received screening for abnormal vaginal flora and urinary tract infections. Both asymptomatic and symptomatic women received antibiotic treatment. The curative rate for abnormal vaginal flora was 58%. The outcome small for gestational age was defined as a birthweight less than 10% cutoff for gestational age and sex based on the Intergrowth-21st standard.

Overall quality of risk estimate: Very low. No systematic review of observational studies on asymptomatic bacteriuria and SGA were identified. The observational study from one non-LMIC concluded no risk relationship between asymptomatic bacteriuria and SGA. Based on randomized control trials, antibiotics were proven to be effective at curing bacteriuria, but not effective at reducing SGA. But we need to be cautious because the cure rate in the randomized control trial were relatively low.

MODEL INCLUSION: Not included. Sufficient evidence of no association between asymptomatic bacteriuria and SGA based on observational studies.

Periodontal disease

Risk: In a 2008 review of periodontal disease and adverse pregnancy outcomes, two observational studies were identified. [20] Both studies defined SGA as birth weight less than the 10th percentile for gestational age based on population standards. A prospective study was conducted in the U.S. from 1997 to 2001 with a sample size of 1017 participants. The analysis adjusted for age, smoking, drugs, marital and insurance status, and pre-eclampsia. Periodontal disease early in pregnancy was associated with an increased risk of an SGA infant (aRR=2.3; 95% C.I.=1.1-4.7) [20] Another prospective study was also conducted in the U.S from 1999 to 2002 with a sample size of 1,365 participants. The study adjusted for potential confounders such as age, race/ethnicity, smoking status, income, frequency of dental check-ups, pre-pregnancy body mass index, pregnancy weight gain, gravidity, prior history of preterm birth and history of genitourinary infection. Periodontal disease early in pregnancy was not statistically associated with delivery of an SGA infant aOR=2.11; 95% C.I.= 0.76–5.86. [20]

Intervention: The 2017 Cochrane review assess the effects of treating periodontal disease in pregnant women. 3 RCTs with a sample size of 3,610 participants reported SGA as one of the outcomes. [21] Two studies were conducted in the US and one in Australia. Periodontal treatments were scaling and root panning or oral hygiene instructions. SGA was defined as birthweight less than 10th percentile based on cohort-specific centile. The Cochrane review concluded no evidence of a difference in SGA when periodontal treatment is compared with no treatment (RR=0.97, 95% C.I. = 0.81-1.16). I^2=54.41%. [22] The risk estimate is inverse of the treatment effect (RR=1.03 [0.86-1.23]). A more recent systematic review on the same topic published in 2019 and the same three studies were included. [23]

Overall quality of risk estimate: Medium. There is a limited and conflicting evidence from observational studies and negative results from intervention trials. We prioritized the evidence with the randomized control trials. In addition, both observational studies and RCTs were conducted in non-LMIC.

MODEL INCLUSION: Not included. Evidence of no association with SGA based on inconsistent conclusion from observational studies and no treatment effect from randomized control trials.

Helminth

Risk: One estimate of risk of helminth on SGA births is from a cross-sectional study of 785 women conducted in 2006 in Ghana. [24] The study investigated plasmodium falciparum and intestinal helminth co-infection. Anemia was defined as hemoglobin levels < 11 g/dL of blood and SGA as sex-specific birth weight at or below the 10th percentile for the weight-for-gestational-age of an international reference population. Models adjusted for age, income, mother's weight and height, previous birth outcomes, gravidity, baby's sex, serum folate, HIV status, malaria and helminth prophylaxis, sickle cell trait and disease, and iron and folic acid supplementations.

Among women with anemia, women who were infected only with intestinal helminths had a statistically significant association with SGA babies (aOR = 2.2; 95% C.I.=1.4-3.8). [24] In non-

anemic women, there was statistically no significant association between intestinal helminths and SGA (aOR =1.2; 95% C.I. = 0.4–2.6). [24]

Intervention: A 2017 systematic review was conducted to assess the impact of deworming. [25] Meta-analysis, randomized trials, quasi-randomized trials, repeated cross-sectional studies, longitudinal studies, and nonrandomized community-based studies were included. None of the studies examined SGA as an outcome. 10 studies provide deworming treatment to pregnant women but the findings on birthweight were inconsistent. 5 studies found beneficial effect on birthweight and 5 studies found insignificant association. After grouping by experimental studies vs. observational studies, all 3 observational studies showed benefit effect on birthweight.

Overall quality of risk estimate: Very Low. No systematic review was found for the association between helminth and SGA. A single cross-sectional study conducted in Ghana reported an increased risk of SGA related to helminth infection only among anemic women. The impact on SGA might be influenced by pregnant women's hemoglobin status. The systematic review on deworming, a common treatment for helminth, showed inconsistent findings on increasing birthweight. If deworming could not increase birthweight, the treatment would likely to have no impact on SGA.

MODEL INCLUSION: Not included. Insufficient evidence of association with SGA based on observational study due to limited availability of evidence.

Trichomonas vaginalis

Risk: The primary estimate of risk of Trichomonas vaginalis on SGA births is based on a systematic review and meta-analysis of 2 studies (N= 72,077), conducted between 1973-2000 in Sweden and the U.S. [26] Trichomonas vaginalis was statistically associated with an increased risk of SGA birth (RR= 1.51; 95% C.I.=1.32–1.73; P ≤ 0.001) (I^2= 0.0%, P= 0.631). [26] Pathogenesis of Trichomonas vaginalis on SGA is unknown and may be quite different to preterm birth. Although speculative, the paper suggests that trichomoniasis may result in chronic low-grade intrauterine inflammation that interferes with uteroplacental circulation.

Overall quality of risk estimate: Low. Only two observational studies conducted in non-LMICs were included. SGA was not clearly defined, and the study did not have adjusted estimates. However, the paper had low heterogeneity and described the methods to assess and address for potential biases.

MODEL INCLUSION: RR= 1.51(1.32–1.73). Evidence based on observational studies.

Bacterial vaginosis

Risk: A 2015 literature review was published to explore the correlation between bacterial vaginosis and fetal growth restriction. [27] The paper neither had a clear definition of fetal growth restriction (FGR) nor was meta-analysis performed. Fourteen studies conducted between 1986-

2010 were included in the review. There is evidence toward abnormal vaginal microflora and impaired fetal growth from four studies, but the conclusions from various studies were inconsistent. Investigators of the Johns Hopkins study found C. albicans to be significantly associated with FGR (OR=1.9, 95% C.I. 1.2-3.14) and M.hominis had no significant association with FGR (N=801, OR=0.96, 95% C.I. 0.59-1.56). [27] Gravett et al found lower mean birthweight infant born to women with BV (N=534). Germain et al. (N=13,914) and Carey et al. (N=4,934) found BV was associated with a significant increased risk of FGR (OR= 1.79, 95% C.I.= 1.27-2.52) and lower birthweight. [27]

Intervention: The 2013 Cochrane review assessed the effects of antibiotic treatment of bacterial vaginosis in pregnancy. [28] A total of 21 trials, involving 7,847 women were included in the review. On comparing placebo to treatment, antibiotic therapy was effective at eradicating bacterial vaginosis during pregnancy (RR=0.42 [0.31-0.56]; 10 trials; 4,403 women; I^2=91%). [28] Antibiotic therapy included any antibiotic (any dosage regimen, any route of administration). SGA was not one of the outcomes assessed. A single RCT study was conducted in Bangladesh with a sample size of 9712 women between 2012 and 2015. [29] SGA was defined as <10% birth weight for gestational age and sex using the Intergrowth 21st standard. The prevalence of abnormal vaginal flora in Bangladesh is 16.5% and bacterial vaginosis is 9.8%. Abnormal vaginal flora was treated with oral clindamycin. RR estimates were adjusted for a priori determined risk factors for adverse pregnancy outcomes (age of pregnant woman at enrollment, asset quintile, prima parity, low MUAC at enrollment, religion, maternal education, household size, and gestational age at enrollment). Being BV infected and not treated was not statistically associated with SGA compared to women without BV (RR=1.23; 95% C.I.= 0.91–1.65). [29] Being BV infected, treated and cured was not statistically associated with SGA compared to women without BV (RR=1.08; 95% C.I.= 0.91–1.28). [29] The risk estimate is the inverse of the treatment effect: RR=0.93 (0.78—1.10). Those who were BV infected, treated and not cured was not statistically associated with SGA compared to women without BV (RR=1.00; 95% C.I.= 0.77–1.29). [29]

Overall quality of risk estimate: Low. Due to the heterogeneity of the study designs, the review of the observational studies did not perform a meta-analysis. Some observational studies found a significant higher risk of fetal growth restriction among BV infected women. Meta-analysis of randomized control trials shown that antibiotic treatment could eradicate bacterial vaginosis. Based on a single LMIC country’s RCT, antibiotic treatment was not effective at reducing risk of SGA.

MODEL INCLUSION: Not included. Insufficient evidence of association with SGA due to limited availability of the evidence.

Influenza

Risk: The estimate is from a prospective, multi-season cohort study of pregnant women conducted in Bangkok, Thailand, Lima, Peru, and Nagpur, India.[30] In the years 2017 and 2018, cohorts of pregnant women were enrolled starting up to 10 weeks before the anticipated start of the influenza season until the fourth week of the influenza season. Influenza was diagnosed

via real-time RT-PCR testing of the nasal swabs from symptomatic women. Small for gestational age infants were defined as an infant with birthweight <10% for infants of the same gestational age and gender based on INTERGROWTH-21 Project Standards. For the SGA analyses, variables such as smoking, exposure to indoor air pollution from cooking fuels, antenatal vitamin use, number of antenatal clinic visits, weeks pregnant in the cohort were adjusted. A total of 10826 women had a complete data on pregnancy and perinatal outcomes in the study and 22% of the women had SGA infants. Having an influenza episode during pregnancy was not associated with an increased risk of having an SGA infant (adjusted relative risk 1.0 95% C.I.= 0.8 to 1.3). [30] Findings were consistent in sensitivity analyses.

Treatment: The 2016 meta-analysis aims to determine the impact of maternal vaccination with either seasonal trivalent inactivated influenza vaccines (IIV) or A/H1N1pdm09 monovalent vaccines on the rates of preterm, small for gestational age, and low birth weight births. [31] The study inclusion also considered plausible effect sizes, baseline rates, and at least 80% power to detect differences for each of the outcomes. For SGA analyses, five retrospective cohort studies published between 2011 to 2014 were included (3 in US and 2 in Canada). All studies were adjusted for potential confounding variables either through multiple variable adjustment, propensity-score adjustment, or by subject to propensity-score matching. All the studies that were included defined SGA as birthweight<10th percentile for gestational age. There was no significant impact of vaccination on SGA birth rates in the meta-analysis independently of the vaccine group (OR=0.95 [0.86-1.06]; 5 studies; I^2=41%). [31] For sensitivity analysis, an RCT from Bangladesh was included and the pooled OR was similar. Stratified analysis by influenza activity also did not show an effect during the period of influenza circulation. A more recent 2019 systematic review included 17 studies that included SGA as an outcome. [32] The estimated adjusted odds ratio of outcome was 0.99 (95% C.I.: 0,94-1.04), suggesting no significant increase or decrease in the risk of SGA births associated with vaccination.

Overall quality of risk estimates: Medium. Adjusted RR from observational studies showed no risk association between influenza with SGA. The multi-site cohort study was conducted in three middle-income countries. Two meta-analysis of the observational studies also showed no significant impact of influenza vaccine no SGA.

MODEL INCLUSION: Not included. Sufficient evidence from observational studies showed no association with SGA births and lack of an effect from intervention trials.

Zika virus

Risk: The primary estimate of risk of zika virus on SGA babies is based on a paper published in 2018 that reviewed the evidence of the effects of congenital Zika virus infection on infant growth. [33] The exposure to the Zika virus during pregnancy was determined by positive result of PCR test for Zika virus in maternal blood, or presence of positive IgM for Zika virus in maternal blood, or probable congenital infection with Zika virus based on clinical-epidemiological criteria of the Brazilian Ministry of Health. Intrauterine growth restriction was defined as fetal weight <10th percentile in the standard curves used. IUGR was observed in approximately 10-18% of the pregnancies among women who were reported to be infected with Zika virus during

pregnancy. The case series of children exposed to Zika virus during pregnancy showed that the prevalence of low birth weight and of infants born small for gestational age may be twice the usual rate. [33] No relative risk estimates were reported.

Another cohort study was conducted from September 2015 through May 2016. [34] A total of 345 women were enrolled, of which, 182 women (53%) tested positive for ZIKV in blood, urine, or both. By 2016, a total of 123 ZIKV-affected and 61 ZIKV-unaffected pregnancies had fetal outcomes. Fetal growth restriction was defined as fetal weight estimated according to the Hadlock formula that was below the 10th percentile. SGA was defined as a z score for birth weight of less than -1.28. Infants who were SGA, as a potential consequence of fetal growth restriction or poor placental perfusion, constituted 8.6% (10/116) of ZIKV-exposed babies and 5.3% (3/57) of ZIKV-unexposed babies (P=0.06). [34] To calculate relative risk, RR=1.64.

Overall quality of risk estimate: Very Low. The two observational studies both showed a potentially increased risk of SGA birth due to zika virus. However, there is no adjusted RR from observational studies. The studies were all conducted in Brazil therefore lack generalizability to other LMICs.

MODEL INCLUSION: Not included. Insufficient evidence to show risk association between zika virus and SGA due to limited availability of studies.

Syphilis

Risk: To measure the impact of maternal syphilis on pregnancy outcome in Tanzania, 380 previously unscreened pregnant women were recruited into a retrospective cohort at delivery and tested for syphilis. IUGR was defined as a birth weight 2500 gm with a gestational age of ≥37 weeks. High-titer active syphilis was associated with an increased adjusted risk of SGA babies (aRR, 2.1; 95%= 1.0–4.2). [35] The RR estimates adjusted for gravidity, delivery site, antibiotic during pregnancy, and maternal stature. The PAF of syphilis associated with SGA babies is 5.2 (95% C.I.=3.3 to 13.7). [35]

Overall quality of risk estimate: Very Low. There is a limited evidence on syphilis and SGA. Other studies that explored infections like syphilis and SGA had also used the risk estimate from the observational study in Tanzania.

MODEL INCLUSION: Not included. Insufficient evidence to show risk association between syphilis and SGA due to limited availability of studies.

Rubella

Risk: A prospective study was conducted in a non-LMIC country, where are total of 1016 women with confirmed case of rubella infection were recruited in the study. [36] The study did not provide information on when the study was conducted but the paper was published in 1982. A significantly higher proportion of seropositive infants had birth weights below the 10th percentile; 25% as compared with 8% of those who were seronegative (but did not provide

information on p value or 95% C.I.). The study did not adjust for any potential confounders and did not have any crude or adjusted risk estimates.

Overall quality of risk estimate: Very Low. There is a limited evidence on rubella and SGA. A couple studies looked at rubella but did not include IUGR or SGA as an outcome. [37 38 39]

MODEL INCLUSION: Not included. Insufficient evidence of association between rubella and SGA due to limited availability of studies.

Measles

Risk: The study was conducted from 2009–2010 to provide a better understanding of the complications associated with measles during pregnancy. The researchers conducted a retrospective cohort study in the aftermath of the large laboratory-confirmed measles outbreak in Namibia. Compared with 172 pregnancies without measles, pregnancies with measles carried significantly increased risks for neonatal low birth weight (adjusted relative risk [aRR] = 3.5; 95% C.I.= 1.5–8.2), spontaneous abortion (aRR = 5.9; 95% C.I., 1.8–19.7), intrauterine fetal death (aRR = 9.0; 95% C.I.= 1.2–65.5), and maternal death (aRR = 9.6; 95% C.I.= 1.3–70.0). [40] The analyses only adjusted for age.

Overall quality of risk estimate: Very low. We did not find any study that examined measles and SGA.

MODEL INCLUSION: Not included. Insufficient evidence of association between measles and SGA due to limited availability of studies.

Toxoplasmosis

Risk: A multicenter prospective cohort study was conducted where women were enrolled from ten European centers that provided prenatal screening for toxoplasmosis. Small for gestational age birth (<10th centile for age and sex standardized birthweight). A total of 620 women were analyzed in the study. Association between infection and birth outcomes were analyzed based on seroconversion before 20 weeks (n=386) and after 20 weeks (n=234). The analysis between maternal toxoplasmosis infection and SGA was standardized for gestational age at birth and sex, and adjusted for country and parity. The study found no significant association between congenital toxoplasmosis and small for gestational age birth when seroconversion occurred before 20 weeks [aOR=1.27; 95% C.I.= 0.45. 3.62)]. [41] Similarly, there was no significant association between congenital toxoplasmosis and small for gestational age birth when seroconversion occurred after 20 weeks [aOR=1.07; 95% C.I.= 0.35, 3.28]. [41]

Overall quality of risk estimate: Very low. There is a limited evidence but the observation study in a non-LMIC showed no increased risk of SGA due to toxoplasmosis.

MODEL INCLUSION: Not included. Insufficient evidence of association between toxoplasmosis and SGA due to limited availability of studies.

Herpes simplex virus

Risk: A population-based case–control study was conducted in Australia with a total sample size of 1326 (cases=717 adverse pregnancy cases; controls=609 children). [42] SGA was defined as <10th percentile. The study did not adjust for any potential confounding factors. The presence of CMV (cytomegalovirus) was not associated with SGA (OR=1.01; 95% C.I.=0.71-1.44). [42] The presence of HSV (herpes simplex virus) was associated with PTB and SGA combined (OR=2.21; 95% C.I.= 1.03–4.73; p value <0.05). No significant association was observed between SGA and exposure to herpes simplex virus infection (OR=1.04; 95% C.I.= 0.49-2.20). [42] No significant association was observed between SGA and exposure to enterovirus infection (OR=0.67; 95% C.I.=0.25-1.82). [42]

Overall quality of risk estimate: Very Low. Different types of herpesvirus all showed no risk association with SGA. However, there is only one observational study in a non-LMIC that looked at herpes and SGA.

MODEL INCLUSION: Not included. Insufficient evidence of association between herpes and SGA due to limited availability of studies.

Environmental and Other Exposure during Pregnancy

Shift work

Risk: A 2013 systematic review assessed the evidence relating preterm, low birthweight, small for gestational age, pre-eclampsia and gestational hypertension to five occupational exposure (working hours, shift work, lifting, standing and physical workload). [43] Eleven studies reported on shift work and SGA. The types of shift work considered relevant varied between studies, some comparing night workers with day workers and others investigating workers on rotating shifts. Moreover, several papers did not specify what kind of shift work was investigated, or classed women as exposed if they worked either at night or in rotating shifts. Some of the studies did not report the timing of exposure during pregnancy while others focused on the first and second trimesters of the pregnancy. It included papers reporting at least one risk estimate for one or more of the specified outcomes, comparing women exposed to shift work with working women who were unexposed (or less heavily exposed). Small for gestational age was defined by cut point on an expected distribution (usually the 10th centile). The pooled estimate is 1.01 (0.92-1.10). [43] When only higher quality studies (N=7) were included, RR=0.98 (0.90-1.08). [43]

Overall quality of risk estimate: Low. Ten studies were conducted in non-LMICs. The quality of the studies was assessed using nine items, no standard quality assessment scales were used. Some of the included studies provided adjusted RR and some only had unadjusted RR. If the study did not adjust for smoking and ≥1 of socioeconomic status, maternal height, or pre-pregnancy weight, the study was classified as having higher potential for confounding.

MODEL INCLUSION: Not included. Sufficient evidence of no association with SGA based on observational studies.

Heavy physical workload during pregnancy

Risk: A systematic review and meta-analysis by Cai et. al included studies conducted between 1983-2016 and were conducted in LMIC or non-LMIC countries. [44] Prolonged standing was defined as standing for =>4 hours per day, prolonged walking as =>4 hours per day. A total of 13 studies (N=39,096) were used to assess the association between prolonged standing, however, to calculate adjusted odds ratio estimates, only 10 studies (N=32110) were used. Prolonged standing during pregnancy was associated with a 19% adjusted increase in the odds of SGA (95% CI=1.08–1.32; I^2=0%). [44] For prolonged walking with SGA babies there were only 4 studies (N=13612) that could be used to calculated adjusted estimates for prolonged walking. Prolonged walking was associated with an increase in adjusted odds of SGA babies (aOR=1.19; 95% CI= 1.03–1.37; I^2=0%). [44] A heavy physical workload was defined as a job that requires heavy physical effort or physical exertion. The current study defined a heavy workload as 1 that met at least 1 of the following criteria: 1) studies report that the job requires "high" or "heavy" physical effort (vs no physical effort); 2) studies assign the job to the highest physical exertion score category; or 3) the job combines >2 physically demanding tasks (e.g., prolonged standing, heavy lifting, and prolonged bending). Although 8 observational studies (N=25,967) were used for physical workload and the outcome, there were only 7 studies (N=24,903) that were used to calculate adjusted risk estimates [44].

Heavy physical workload was associated with an increased adjusted odds of SGA babies (aOR= 1.26; 95% CI= 1.01–1.56; I^2=24%) [44]. Using the data provided, we estimated that prevalence of heavy physical workload was 67%. This was used to convert the adjusted odds ratio to an estimated adjusted aRR= 1.07 (95% C.I. 1.00–1.13).

Overall quality of risk estimate: Medium. There was evidence from meta-analysis of observational studies (prospective cohort, case-control, and cross-sectional) after adjusting for potential confounders.

MODEL INCLUSION: RR=1.07 (1.00–1.13). Evidence from observational studies.

Intimate partner violence

Risk: The primary estimate of risk of intimate partner violence is based on a systematic review and meta-analysis by Donovan et. al that included studies published between 1994-2014. [45] A total of 7 studies (N=90,496) were used to assess the association between intimate partner violence (physical, emotional, and sexual violence) and SGA babies. Five of the seven studies that assessed SGA were conducted in high income countries. There were two cross-sectional studies that belonged to high income countries and rest of the other studies were cohort studies.

The unadjusted pooled OR for SGA outcomes was significantly increased among women who experienced IPV (Intimate partner violence) during pregnancy, compared with women who did not (OR =1.37, 95% C.I.= 1.02–1.84, I^2=32%). [45] The adjusted pooled OR for SGA outcomes was not significantly associated between IPV and SGA babies (aOR= 1.6; 95% C.I.=0.61–4.1, I^2=78%). [45] The adjusted analysis only included 2 studies. However, when only studies conducted in LMICs (2 studies) were analyzed, the unadjusted increased odds were not statistically significant (OR= 1.32; 95% C.I.= 0.87–2.02). [45] Although the OR is relatively high the small sample size could influence the statistical significance of the association. Women who experienced IPV in LMICs had a statistically significant increased unadjusted odds of having PTB babies. This result was observed in studies that were either cross sectional or case-control in design.

A meta-analysis of 4 studies (2 in Canada, 1 in US, and 1 in Nicargua) also did not find significant association between IPV and SGA/IUGR (aOR= 1.46; 95% C.I.= 0.65–2.27) I^2=0.16; p=0.001. [46] The study explored physical, psychological, sexual, emotional types of violence and adjusted for confounding factors. [46]

Overall quality of risk estimate: Low. Based on the adjusted estimates, there was evidence of no risk from meta-analysis of observational studies.

MODEL INCLUSION: Not included. Sufficient evidence of no association with SGA based on observational studies.

Stress during pregnancy

Cero´n-Mireles et al. enrolled 2623 women to conduct an observational study in Mexico and the study was published in 1996. [47] The analysis adjusted for maternal age, education, parity, history of LBW, and medical conditions during pregnancy. Conflicts at work predicted SGA only among women who delivered at the public assistance hospital (aOR = 4.93, CI = 2.09, 11.66). It is important to note that the public assistance hospital typically sees the poorest women.

Koen et al. enrolled 544 pregnant women to conduct prospective cohort study in South Africa and the study was published in 2016. [47] Women with posttraumatic stress disorder (PTSD) did not predict SGA. Questionnaires were used to assess PTSD.

Ruwanpathirana et al. enrolled 835 women to conduct a case-control study in Sri Lanka. A self-administered questionnaire was used to assess the psychological stress level of the pregnant mothers. High stress levels during the second trimester predicted SGA in univariate (unadjusted OR = 2.17, CI = 1.43, 3.30) and multivariate (adjusted OR = 1.92; CI = 1.17, 3.14) analyses. [47]

Valladares et al. enrolled 147 women to conduct a cross-sectional study in Nicaragua and published the study in 2009. [47] Cortisol level was analyzed through two salivary samples that were collected at different times of the day. A significant association was found between violence during pregnancy and SGA births (p = 0.000), but not with preterm deliveries (p = 0.298). However, the study did not provide risk estimates. Violence during pregnancy was

associated with an increased PM cortisol level but the study did not explore association between cortisol level and SGA.

Overall quality of risk estimate: Very low. The systematic review had observational studies that were conducted in LMICs and non-LMICs but did not have pooled estimate. Not all the studies calculated risk estimates and the studies that had risk estimates did not adjust for confounding factors.

MODEL INCLUSION: Not included. Insufficient evidence of association with SGA due to lack of meta-analysis

Secondhand smoking

Risk: A 2010 systematic review and meta-analysis which included 18 observational studies (n=20297), conducted between 1990-2004 was available. [48] The paper did not clearly mention which countries did the studies belonged to that were used for SGA analysis. SGA was defined as birthweight < 10th percentile for gestational age and exposure as passive maternal smoking. ETS (Environmental tobacco smoke) exposure was defined either as a positive response on a patient questionnaire or biochemical assays within the accepted passive smoking range (serum, hair, or saliva measurements of 2–10 ng/ml of cotinine; none of the included studies assayed nicotine). Studies were excluded if mothers actively smoked or if it could not be determined from the article if active maternal smoking was a confounding factor. The analysis did not adjust for any potential confounders. There was no significant difference in SGA between pregnant women exposed to environmental tobacco versus those who were not exposed OR 1.06 (95% C.I.= 0.75–1.50). [48] An older systematic review found an increased risk of SGA among women exposed to environmental tobacco smoke only in retrospective studies (adjusted OR=1.21 [1.06-1.37]; 9 studies), but not in prospective studies (adjusted OR=1.05 [0.87-1.28]; 9 studies). [49]

The 2020 systematic review which included 28 cohorts from Europe and North America also assess the impact of paternal smoking on SGA. Among nonsmoking mothers, parental smoking was associated with higher risk of SGA (OR=1.04 [1.00-1.09]). [50] The prevalence of parental smoking among men included in the systematic review was 21.46%, which converted the OR to RR= 1.03 (1.00-1.07). Child's sex, maternal education level, parity, and alcohol consumption during pregnancy, maternal age, pre-pregnancy or early-pregnancy maternal and paternal BMI were adjusted in the meta-analysis. [50]

Intervention: A systematic and meta-analysis of 3 studies was conducted between 2012-2013 to investigate the effect of smoke-free legislation on perinatal and child health in Ireland, Belgium, and UK [51]. Smoke-free legislation was defined as smoking ban in workplaces, public places, or both. Small for gestational age was defined as a birthweight below the 5th centile for gestational age or as a birthweight below the 10th centile by others. Introducing smoke-free legislation did not have statistically significant reduction in SGA (Risk change= -1.40%; 95% C.I.= -3.20 to 0.40) [51]. The meta-analysis used 3 studies with 1,905,153 participants.

Overall quality of risk estimate: Medium-Low. There was an inconsistent evidence based on the observational studies, especially in the case of paternal smoking. Since the paternal smoking is the major exposure of secondhand smoking for pregnant women, we think it is necessary to include secondhand smoking as a risk factor.

MODEL INCLUSION: RR=1.03 (1.00-1.07). Evidence of association with SGA based on observational studies.

Indoor air pollution

Risk: A 2014 systematic review examined the relations between solid fuel use at home and pregnancy outcomes. [52] A total of 19 studies were included in the review. Most studies examined birthweight and low birth weight as outcomes. Two studies (one in Ghana and one in India) assessed term LBW; a proxy for intrauterine growth retardation (IUGR). Exposure to household air pollution was associated with higher risk of IUGR (summary-effect estimates=1.23 [1.01-1.49]; I2=0%). [52] Exposure to household air pollution was assessed indirectly using questionnaires to solicit information on primary cooking and heating fuels used by maternal households routinely or during the index pregnancy. Potential confounders including demographic, household and socioeconomic factors, maternal nutritional, health and lifestyle factors; neonatal characteristics; and second-hand smoke exposure were controlled. Detailed confounding factors were not provided.

A population cohort study was conducted in India from 1998 to 2000, where a total of 11728 live born infants were analyzed. [53] Exposure to indoor particulates due to the use of solid biomass fuel sources was defined as the use of wood or dung as the primary fuel source for cooking in the household compared with biogas or kerosene. Infants below the 10th percentile of weight-for-GA at birth were considered SGA. Low birth weight (LBW) estimates are also calculated. SGA was statistically higher among infants born into households that used wood or dung as cooking fuel (aRR = 1.21; 95% C.I.=1.11 to 1.31). [53] The analysis adjusted for number of children <5 years of age in the household, place of delivery, roof material, religion, maternal night blindness, maternal age, maternal education, parity, television/radio ownership, electricity in the household and SHTS exposure.

Another prospective cohort study analyzed 1323 women in Ghana between 2018 and 2019. [54] SGA was defined as birthweight less than the 10th percentile. The study extracted exposure questions from indoor air pollution district survey questionnaire (Indoor Air Pollution District Survey Questionnaire, Questionnaire, 2020) to assess indoor air quality. Babies of mothers who used charcoal did not have statistically significant association with SGA (aRR=1.72 times; 95% C.I. 0.52–5.65) compared to those who used gas/electricity/biogas. [54] Similarly, using firewood also did not have statistically significant association with SGA babies (aRR=1.70 times; 95% C.I.= 0.49–5.92) compared to those who used gas/electricity/biogas. [54] The risk estimates controlled for maternal BMI at first visit and anemia.
Similarly, another birth cohort study conducted from 2010-2012 in China with a total of 9,895 was analyzed. [55] Compared to using a gas stove for cooking, use of biomass (aOR = 1.22, 95 % CI= 0.70–2.08), coal (aOR = 1.27, 95 % CI: 0.90–1.80), and electromagnetic stoves (aOR = 1.27,

95 % CI= 0.93–1.74) were not significantly associated with the risk of SGA. [55] The analysis adjusted for maternal age, education, family income, maternal weight gain, vitamin supplement during pregnancy, preeclampsia, caesarean section, parity, smoking, and ventilation.

A study conducted a secondary analysis of data from two cross-sectional cohorts in India. [56] In one study, enrollment occurred over a 12-month period in 2006 at urban and rural facilities. In the other study, recruitment occurred from 2007 to 2008 in urban and rural facilities. Women were interviewed about the primary cooking fuel used in their household and the average number of hours per day they spent cooking during their pregnancy. A total of 1744 of women was analyzed. SGA is defined if their birth weight was less than the 10th percentile for gestational age using an India specific derived reference curve. 74.9% used wood in their homes as the primary cooking fuel. Women using wood was not significantly associated with the risk of SGA aOR= 0.95 (95% C.I.= 0.58, 1.57) compared to those who used gas. [56] The analysis adjusted for cohort site/location, maternal age, body mass index, gravidity, hemoglobin at delivery, and time spent cooking.

Interventions: Two sequential trials were conducted in rural area of Nepal from 2010 to 2014. [57] Traditional mud brick biomass stoves were commonly used. Trial 1 was a cluster-randomized step-wedge trial comparing traditional biomass stoves and improved biomass stoves vented with a chimney. Trial 2 was a parallel household-randomized trial comparing vented biomass stoves and liquid petroleum gas stoves with a year's supply of gas. The sample size for both trials was based on number of households needed to detect a difference in acute lower respiratory infection rates. A total of 2,379 live born infants were enrolled in trial 1 and 270 and 279 in the biomass stoves and liquid petroleum gas stoves groups in trial 2. CO and $PM_{2.5}$ were measured prior to and after the stove installation. SGA was defined as those whose sex- and gestational-age-specific birth weights fell below the 10th percentile of the intergrowth population distribution using the upper bound of the weekly published data. Compare to traditional biomass stoves, vented biomass stoves did not reduce the risk of SGA (RR=1.00, 95% C.I.: 0.74, 1.34) [57] . Compare to vented biomass stoves, liquid petroleum gas stoves did not reduce the risk of SGA (RR=0.98, 95% C.I.: 0.79, 1.21) [57].

Overall quality of risk estimate: Medium. There was inconsistent evidence from four single observational studies, but the point estimates were similar across different studies. The systematic review found significant risk association between indoor air pollution and SGA. But the systematic review used term LBW--a proxy of SGA as an outcome. In addition, there is no evidence of a strong intervention effect, perhaps due to the intervention not reducing exposure sufficiently. Therefore, we decided to exclude indoor air pollution in the primary model but to included it in the extended model.

MODEL INCLUSION: 1.23 (1.01-1.49) Evidence of association with SGA based on observational studies and only used in the extended model.

Ambient air pollution

Risk: The primary estimate of risk of ambient air pollution is from a systematic and meta-analysis of 6 cohort or cross-sectional studies that were conducted from 1996 to 2009 (N= 151,5887). [58] The studies were conducted in non-LMIC countries (Canada, Australia, UK, and 3 from US). The individual studies adjusted for potential confounders in their analysis, but the number of confounders differed among the studies. Exposure assessment methods among most of the studies were often based on existing ambient monitoring stations, pollutant dispersion or land-use regression model, but few studies used satellite data and personal monitoring data. SGA was defined as weight below the 10th percentile of infants born at a given gestational age. Low birth weight (LBW) estimates were also calculated.

With regards to the exposure in the entire pregnancy period, a 10 μg/m3 increase in PM2.5 was positively associated with SGA babies (OR = 1.15; 95 % CI, 1.10–1.20; p value <0.001) I2 = 0%. [58]

Majority of the studies were conducted in the USA, so the prevalence estimates from the GBD 2015 of $PM_{2.5}$ exposure (17·5%) was used to convert the OR to an RR= 1.12 (1.08-1.16). [58]

During 1st trimester pregnancy, a 10 μg/m3 increase in PM2.5 was positively associated with SGA (OR = 1.07; 95 % CI= 1.05–1.10; p value <0.001) [58] . I^2 = 5%.

During 2nd trimester pregnancy, a 10 μg/m3 increase in PM2.5 was positively associated with SGA (OR = 1.06; 95 % CI= 1.02–1.10; p value 0.004) [58]. I^2 = 58.1%.

During 3rd trimester pregnancy, a 10 μg/m3 increase in PM2.5 was positively associated with SGA (OR = 1.06; 95 % CI= 1.04–1.08; p value <0.001) [58]. I^2 = 13.4%.

A retrospective cohort study was conducted from 2015 to 2017 with a sample size of 506,000 births in China. [59] Except for 4 districts, one station in each district measured 24 hour averaged daily ambient air pollutant concentrations (in μg/m^3), including particulate matter with an aerodynamic diameter less than or equal to 10 μm or 2.5 μm (PM_{10} and $PM_{2.5}$, respectively), nitrogen dioxide (NO_2), and sulfur dioxide (SO_2). In the case of ozone (O_3), a daytime 8-hour average of each day was used. SGA was defined as birth weight less than the 10th percentile. The analysis adjusted for covariates, including maternal age, parity, season of conception, medical conditions during pregnancy, delivery mode, and infant sex.

The adjusted ORs between $PM_{2.5}$ (10ug/m^3) exposure during the entire pregnancy and SGA was 1.02 (95% C.I.= 1.00–1.04). [59] The adjusted ORs between NO_2 exposure during the entire pregnancy and SGA was 1.08 (95% C.I.= 1.04–1.12). [59]The adjusted ORs between SO_2 exposure during the entire pregnancy and SGA was 1.02 (95% C.I.= 1.01–1.03). [59] The adjusted ORs between O_3 exposure during the entire pregnancy and SGA was 1.14 (95% C.I.= 1.11–1.17). [59] The adjusted ORs between PM_{10} exposure during the entire pregnancy was not statistically associated with SGA 1.01 (95% C.I.= 0.99–1.03). [59]

Intervention: A systematic review and meta-analysis of thirty-seven studies was conducted to explore the association between residential green and blue spaces and pregnancy outcomes. [60] Different approaches were performed in the studies where exposure was treated differently. Normalized difference vegetation index (NDVI), a green biomass density indicator was used as the estimate of surrounding greenness. The study did not provide a definition for SGA. The individual studies adjusted for covariates in their analysis, though the number of covariates differed among the studies. To explore association between the residential green and blue

spaces and SGA, a systematic and meta-analysis of 12 cross sectional studies was done where studies were conducted from 1999 to 2019 (N= 415,1031). The studies were conducted in non-LMIC countries (Canada, Sweden, Israel, Lithuania, 2 from Spain, and 6 from US). [60] Residential greenness within 300 m buffer was weakly but statistically associated with reduced odds of SGA birth (OR=0.95; 95% C.I. =0.92, 0.97), I^2=57%[60]. A 0.1 unit increase in NDVI (green density indicator) was positively associated with SGA (OR = 0.95; 95 % CI= 0.91- 0.99), I^2=55%. [60]

Overall quality of risk estimate: Medium. A systematic review and meta-analysis paper, where the studies were conducted in non-LMICs. The sample size is large with low heterogeneity.

MODEL INCLUSION: RR= 1.12 (1.08-1.16). Evidence of association with SGA based on observational studies.

Smoking

Risk: A total of 28 cohorts from Europe and North America were included. 22 cohorts were population-based, with birth years ranging from 1991 to 2005. [50] The exposures examined in the review were quitting or reducing smoking during pregnancy and combination of maternal and paternal smoking. Maternal smoking was first categorized by trimester: nonsmoking; first-trimester-only smoking; continued smoking. Then trimester-specific maternal smoking information was categorized into nonsmoking, ≤4 cigarettes/day, 5-9 cigarettes/day, and ≥10 cigarettes/day. Paternal smoking was categorized into smoking and nonsmoking. Taking both maternal and paternal smoking into consideration, there were five categories: (1) maternal and paternal nonsmoking (reference category); (2) maternal nonsmoking and paternal smoking; (3) maternal first trimester-only smoking and paternal nonsmoking; (4) maternal first-trimester-only smoking and paternal smoking; (5) maternal continued smoking and paternal nonsmoking; (6) maternal continued smoking and paternal smoking. The outcome small size for gestational age was defined per cohort as sex- and gestational age-adjusted birth weight below the 10th percentile.

Compared to nonsmoking mothers, mothers who only smoked during first trimester, regardless of numbers of cigarettes per day, did not have a higher risk of having small sized for gestational age newborns (OR=0.99 [0.85-1.15]). [50] Compared to nonsmoking mothers, mothers who continued smoking during pregnancy had a significantly higher risk of having SGA babies (OR=2.15 [2.07-2.23]). [50] The prevalence of continued smoking among the cohorts included in the review was 13.4%, which can be used to convert OR to RR=1.86 (1.81-1.91). There were also dose-related association between number of cigarettes per day and the risk: ≤4 cigarettes/day (OR=1.57 [1.45-1.70]); 5-9 cigarettes/day (OR=2.40 [2.25-2.56]); ≥10 cigarettes/day (OR=2.93 [2.76-3.10]). [50]

Reducing the number of cigarettes, without quitting, from first to third trimester lower the risk of delivering SGA infants, but risks were still higher compared with those of nonsmoking mothers;(OR=2.79 [2.39-3.25] when reducing from ≥10 to 6-9 and 1.93 [1.46-2.57] when reducing from ≥10 to ≤4 cigarettes/day. [50] Similarly, the OR was1.89 [1.52-2.34] when women

reduced smoking from 5-9 to ≤4 cigarettes/day. The OR for SGA was 2.43 [2.05-2.89] when increasing from <=4 to 5–9 cigarettes/day.[50]

Overall quality of risk estimate: Low. Child's sex, maternal education level, parity, and alcohol consumption during pregnancy, maternal age, pre-pregnancy or early-pregnancy maternal and paternal BMI were adjusted in the meta-analysis. The review only included cohort studies in Europe and North America. No information was available for LMICs.

MODEL INCLUSION: RR=1.86 (1.81-1.91). Sufficient evidence of association with SGA from observational studies.

Alcohol consumption

Risk: A 2017 systematic review assessed the effects of low-to-moderate levels of maternal alcohol consumption on pregnancy and longer-term offspring outcomes. [61] Epidemiological studies sampled from general population, quasi-experimental studies, and negative control studies were included. The definition of low alcohol consumption was up to 32g/week. Small for gestational age was defined as <10th percentile in weight or <-2SD scores. The review included 24 prospective studies and 7 studies assessed SGA as an outcome. Compared to no alcohol drinking, low alcohol drinking during pregnancy was associated with an increased the risk of SGA (OR=1.08 [1.02-1.14]; 288,512 participants). [61] The prevalence of low alcohol consumption was not available; therefore, the OR could not be converted to RR. One US study contributed to 95% of the participants. Confounding factors, such as maternal smoking, socioeconomic position and ethnicity were controlled in the analysis. Newcastle-Ottawa scale as used to assess the risk of bias for included reports.

An older systematic review that used 8 observational studies (6 cohort; 2 case control), assessed the dose-response association of maternal alcohol exposure before and during pregnancy. [62] The study included in the paper were conducted only high-income countries (Canada, United States, the UK, New Zealand, and Australia). All studies on SGA adjusted for confounders (such as smoking, socio economic status, body mass index, etc.). SGA was defined as 10th percentile of gestational age adjusted birth weights. Alcohol consumption was measured in number of drinks, where 1 standard US drink=12 grams. Comparing any alcohol consumption to no alcohol consumption, the adjusted pooled relative risk did not show a statistically significant association between alcohol consumption and SGA babies, and there was a high heterogeneity (aRR=0.99; 95% C.I.=0.89-1.10; I^2 = 82% and p <0.0001). [62] The risk of SGA with alcohol consumption showed significantly linear increase in mothers who consumed an average of 2 drink or more per day. With an average of 2 or more drinks a day, risk of having SGA looks more apparent (aRR=1.17; 95% C.I.= 1.03-1.32). [62] Similarly, there was a statistically significant association between increased alcohol consumption and increased risk of SGA; 3 drinks aRR=1.32; 95% C.I.=1.12-1.55); (4 drinks aRR=1.48; 95% C.I.=1.21-1.81); (5 drinks aRR=1.65; 95% C.I.=1.29-2.10); (6 drinks aRR=1.83; 95% C.I.=1.38-2.42); (7 drinks aRR=2.02; 95% C.I.=1.47-2.77). [62]

Overall quality of risk estimate: Low. There was consistent evidence from systematic reviews of observational study. We also found a dose-dependent relationship between alcohol consumption and SGA. The newer systematic review identified increased risk of SGA among low alcohol consumption (1 to 2 standard drinks).

MODEL INCLUSION: RR=1.17 (1.03-1.32). Sufficient evidence of association with SGA based on observational data.

Acrylamide exposure

Risk: The pooled OR of three studies revealed an association between maternal acrylamide exposure and SGA (crude OR=1.14 [1.06-1.23], P<0.001). [63] The summary estimate was pooled from comparisons between higher exposure with lower exposure, using the lowest quantile group as reference. Two studies used food frequency questionnaires and one study used Hb adducts of acrylamide to measure acrylamide exposure. Subgroup analysis by smoking status, acrylamide exposure measure method, partition of exposure level, and study quality were performed. No significant difference on SGA were found between highest vs. lowest exposure group. In the sensitivity analysis, after controlling for potential confounders, the pooled OR of high exposure vs. low exposure group for SGA was 1.11 (1.02-1.20, P=0.017). [63] Adjusted factors were gestational age, parity, sex of the child, maternal age, maternal BMI, gestational weight gain, smoking during pregnancy, maternal age at delivery, specific maternal weight gain during pregnancy. All three studies were conducted in Europe, including a total of 52,835 women. One study conducted in Norwegian included 50,651 participants. Using Newcastle-Ottawa Scale, two studies were deemed high quality and one study were deemed medium quality.

Overall quality of risk estimate: Low. There was evidence from meta-analysis of observational studies to show the potential association between acrylamide exposure and SGA in high-income countries. And the risk remained after controlling for confounders. However, the measurement method for acrylamide exposure (food frequency questionnaires) is hard to validate. The estimate of the prevalence of acrylamide exposure is not available in LMICs.

MODEL INCLUSION: Not included. Weak evidence of association with SGA due to lack of evidence in LMICs and limited prevalence of acrylamide exposure in LMICs.

Pregnancy History

Maternal age and parity

Risk: The primary estimate of risk on SGA babies is based on a systematic review and meta-analysis by Kozuki N et. al that included studies conducted between 1993-2004 and published in 2013. [64] Maternal age and parity are often cited in the literature as independent risks for preterm birth. However, it is very difficult to separate the risks entirely; younger women are more often primiparous and older women often have higher parity. In the analysis below, comprised of 14 cohort studies from multiple countries. Due to the interconnectedness of the relationship, we will be considering these two risk factors together in the analysis. SGA was

defined as below the 10th percentile of the U.S. 1991 reference distribution described by Alexander and colleagues. The estimate adjusted for potential confounders (socioeconomic and maternal nutritional variables). In the analysis, all except one category (older maternal age >35 and parity >3) was based on a meta-analysis of 14 prospective cohort studies with a sample size of 90168. For the analysis of "older maternal age >35 and parity >3" section, it was a meta-analysis of 13 prospective cohort studies. Primiparous women, regardless of their age were at higher risk of SGA birth compared to multiparity of 1-2.

Overall quality of risk estimate: Medium. The 14 prospective cohorts included in the analysis result in a large sample size in 9 different countries in Asia, Africa, and South America. The adjusted ORs estimated in the analysis were adjusted for the following potential confounders: maternal education, ethnicity, land ownership, housing characteristics, income, antenatal care, height, weight, and MUAC. It is unclear why there was a slight, but significant protective association between parity ≥3 and SGA. With the parity ≥5 cut-off, the magnitude of the associations remained almost identical, although the associations lost statistical significance. The lack of change in risk with increasing parity may again hint that confounders are not captured by the covariates.

I. Young maternal age < 18 & Primiparity

The risk estimate for the association between primiparity, maternal age <18 years, and SGA birth is aOR= 1.8 (95% C.I.= 1.36-1.60), compared to women ages 18-35 with multiparity 1-2 after adjusting for potential confounders. [64] There was a similar statistically significant trend between the outcome and young (<18 years) primiparous women. The prevalence of primiparous women with a maternal age <18 years was 7.37%, which was used in the conversion of the adjusted odds ratio to an adjusted risk ratio equal to aRR=1.70 (1.32- 1.53).

MODEL INCLUSION: aRR=1.70 (1.32- 1.53). Evidence of association with SGA based on observation studies.

II. Maternal age 18-35 and Primiparity

The risk of SGA birth among primiparous women between 18 and <35 compared to women ages 18-35 with parity 1-2 was significant (aOR= 1.51, 95% C.I.= 1.39-1.64) with adjustment for potential confounders. [64] However, the risk of preterm birth among primiparous women between 18 and <35 compared to women ages 18-35 with parity 1-2 was statistically insignificant. The prevalence of primiparous women with a maternal age of 18-35 years was 28.27%, which was used in the conversion of the adjusted odds ratio to an adjusted risk ratio equal to aRR=1.32 (1.25-1.39).

MODEL INCLUSION: aRR= 1.32 (1.25-1.39). Evidence of association with SGA from observational studies.

III. Maternal age 18-35 and multiparous

The parity ≥3 and age 18 to <35 category did not have an adverse association with SGA birth compared to women of the same age with a parity of either 1 or 2. Instead, there was a small

but statistically significant protective effect against SGA (aOR= 0.92, 95% C.I.= 0.86-0.99) after adjusting for potential confounders. [64] However, the association was in the opposite direction between women of 18 to <35 and parity >3, and preterm birth, where there was a significantly increased odds of PTB (OR=1·20; 95% C.I.= 1·06-1·35) compared to women of the same age with a parity of either 1 or 2. [64]. The prevalence of this age and parity category was equal to 13·42%, which was used to convert the aOR to aRR=0.93 (0.88-0.99).

MODEL INCLUSION: Not included. Sufficient evidence of no risk for SGA birth based on observation studies.

IV. Older maternal age >35 & Parity >3

Risk: The risk estimates for the association between parity > 3, maternal age >35 years, and SGA birth was not statistically significant (aOR= 0.98, 95% C.I.=0.87, 1.09) after adjusting for potential confounders. [64] However, the association was in the opposite direction between parity > 3, maternal age >35 years, and preterm birth, where there was a significantly increased odds of PTB (OR=1·43; 95% C.I.= 1·21-1·69) compared to women of the 18-35 with a parity of either 1 or 2.

MODEL INCLUSION: Not included. Sufficient evidence of no association with SGA birth based on observation studies.

Birth interval

Risk: The risk estimates for the association between short birth interval (< 18 months) compared to recommended pregnancy intervals (36-60 months) is from a meta-analysis of 5 datasets representing three countries (3 from Brazil, 1 from Philippines, and 1 from Zimbabwe). [65] It had a sample size of 32,670 with studies conducted between 1982 to 2004, where 4 studies were longitudinal and 1 was RCT designed study. SGA was defined as below the 10th percentile of a gender-specific reference distribution at each completed gestational week, using births in the US in 1991. The analysis controlled for parity, age, and available socioeconomic, nutritional, and reproductive health confounders in each dataset to calculate adjusted odds ratios (aOR).

Overall quality of risk estimate: Medium. The systematic review used large data sets from 5 LMICs. Outcome and exposures were clearly defined and hence was able to meta-analyze five studies with the same exposure and outcome definitions. The analysis also controlled for potential confounders.

I. Birth interval <18 months

Birth interval of < 18 months had a statistically significant increased odds of SGA birth (pooled aOR= 1.51, 95% C.I.= 1.31-1.75) compared to recommended pregnancy interval (36-60 months) after controlling for potential confounding factors. 65 Using the prevalence of 10% from the paper, the conversion of the odds ratio to a risk ratio equal to aRR=1.44 (1.27-1.63).

MODEL INCLUSION: aRR= 1.44 (1.27-1.63). Evidence of association with SGA from observational studies.

II. Birth interval 18-<24 months

Birth interval of 18- <24 months had a statistically significant increased odds of SGA (pooled aOR= 1.23, 95% C.I.= 1.03-1.48) compared to recommended pregnancy intervals (36-60 months); after controlling for potential confounding factors. [65] Using the 11.72% prevalence from the paper, the conversion of the odds ratio to a risk ratio equal to aRR= 1.20 (1.03-1.40).

MODEL INCLUSION: aRR= 1.20 (1.03-1.40). Evidence of association with SGA from observational studies.

III. Birth interval >60 months

Birth interval of over 60 months had a statistically significant increased odds of SGA (pooled aOR= 1.22, 95% C.I.= 1.07-1.39) compared to recommended pregnancy intervals (36-60 months), after controlling for potential confounding factors. [65] Using the 28.96% prevalence from the paper, the conversion of the odds ratio to a risk ratio equal to RR=1.15 (1.05-1.25).

MODEL INCLUSION: aRR=1.15 (1.05-1.25). Evidence of association with SGA from observational studies.

Previous history of SGA

Risk: The primary estimate of risk on SGA babies is from an observational study that used 2 data sets (1st from 2006-2016; 2nd from 2014-2018). [66] Both were from the UK and a total sample size of 31,652. SGA= birthweight <10th percentile for gestational age at delivery, based on the Fetal Medicine Foundation fetal and neonatal population weight charts. OR adjusted for age, weight, height, race, ovulation drugs, IVF conception, smoking, chronic hypertension, diabetes, parity.

Previous history of SGA infant in parous women was statistically associated with SGA compared to nulliparous women without prior history of SGA (aOR=1.23; 95% C.I.= 1.17–1.29). [66]

Another single observational study conducted in Australia among those who gave birth between 2007 to 2010. [67] SGA was defined as less than the 10th percentile of Australian national gestational age- and sex-specific birthweight percentiles.

- <u>In Preterm birth (n=15541 birth)</u>

Previous history of 1 SGA infant was statistically associated with SGA compared to women without prior history of SGA (crude OR=2.06; 95% C.I.= 1.75- 2.42). [67]
Previous history of 2 SGA infants was statistically associated with SGA compared to women without prior history of SGA (crude OR=3.26; 95% C.I.= 2.37-4.48). [67]

- <u>In Term infant, diabetic mother (n=17292 birth)</u>

Previous history of 1 SGA infant was statistically associated with SGA compared to women without prior history of SGA (crude OR=1.89; 95% C.I.= 1.58-2.25). [67]
Previous history of 2 SGA infants was statistically associated with SGA compared to women without prior history of SGA (crude OR=3.40; 95% C.I.= 2.22-5.19). [67]

- <u>In Term infant, non-diabetic mother (n=266357 birth)</u>

Previous history of 1 SGA infant was statistically associated with SGA compared to women without prior history of SGA (crude OR=2.41; 95% C.I.= 2.31-2.52). [67]
Previous history of 2 SGA infants was statistically associated with SGA compared to women without prior history of SGA (crude OR=5.42; 95% C.I.= 4.92-6.00). [67]
Previous history of 3 SGA infants was statistically associated with SGA compared to women without prior history of SGA (crude OR=7.70; 95% C.I.= 6.19-9.58). [67]

Overall quality of risk estimate: Very low. The risk estimate was from a single study conducted in non-LMIC and did not adjust for any potential confounders in the analysis.

MODEL INCLUSION: Not included. Weak evidence of association with SGA due to limited availability of studies and lack of data from LMICs. More importantly, potential of this not representing an independent risk.

Previously induced abortion

Risk: A systematic review and meta-analysis was conducted to evaluate the risk of PTB among women with a history of uterine evacuation for I-TOP or SAB. [68] I-TOP was defined as an intervention to voluntarily terminate a pregnancy (induced abortion) and SAB as spontaneous abortion (spontaneous intrauterine pregnancy loss prior to 20 weeks). SGA was defined as birthweight < 10th percentile for gestational age.
For SGA outcome, 3 studies were used with a sample size of 43,411 women. The 3 observational studies were conducted between 1989 to 2011 in non-LMIC countries (France, Finland, and the 3rd study collected data from New Zealand, Australia, Ireland, and UK).

Women with a history of uterine evacuation for either I-TOP or SAB had a significantly higher risk of SGA babies (OR= 1.19; 95% C.I.= 1.01-1.42; I^2= 57%; P value=0.10) compared with controls. Control group are those without prior uterine evacuation. Women with a prior surgical I-TOP had a significantly higher risk of SGA (OR= 1.19, 95% C.I.= 1.01-1.42) compared with controls. No estimates were provided for those with only SAB and SGA babies.
It was a similar association in case of PTB (aOR= 1.44, 95% C.I.= 1.09-1.90). The analysis adjusted for confounding factors only for PTB but not for SGA. [68]
Another systematic and meta-analysis of 3 studies was conducted in Scotland, France, Finland from 1989-2001. [69] I-TOP was defined as an intervention performed using vacuum aspiration or were medically induced to terminate a pregnancy (induced abortion). SGA was defined as birthweight < 10th percentile for gestational age. A history of one I-TOP (induced abortion) was not statistically associated with SGA compared with those without history of induced abortion (adjusted OR=0.99, 95% C.I.= 0.86–1.16, I^2=64%). [69] A history of more than one previous I-TOP (induced abortion) was not statistically associated with SGA compared with those without history of induced abortion (adjusted OR=1.06, 95% C.I.= 0.84–1.33, I^2=0%). [69]

Overall quality of risk estimate: Very Low. The evidence from systematic and meta-analysis of observational studies were only from non-LMIC countries and did not adjust for any potential confounders in the analysis. In addition, there were inconsistent evidence from two systematic reviews.

MODEL INCLUSION: Not included. Insufficient evidence of association with SGA due to frequent inconsistent evidence.

Maternal Nutritional Status

Maternal stature

Risk: The study by Kozuki et. al. conducted a systematic review and meta-analysis to estimate the association between short maternal stature and SGA birth. [70] A total of 12 prospective cohort studies from LMIC, with a sample size of 40,375 live births (4 studies from Asia, 4 from Africa, and 4 from the Americas). SGA as a birth weight below the 10th percentile of a sex-specific birth weight distribution by gestational age. the height cutoffs of ‘‘short stature’’ ranged from <155 cm to <173 cm.

Women <145 cm had the highest adjusted RRs for term SGA (aRR: 2.03; 95% C.I.: 1.76- 2.35; p <0.001) and preterm SGA (aRR= 2.13; 95% C.I.= 1.42, 3.21; P = 0.031). [70] Population attributable fraction (PAF) of short stature on Term-SGA is 18.6% (95% C.I.= 17.4- 19.7); PAF of short stature on Preterm-SGA is 16.6% (95% C.I.= 13.7- 18.9). [70]

Overall quality of risk estimate: Low. The paper included observational studies, which were all from LMICs. All the prospective cohort studies from LMICs controlled for available confounders in the analysis.

MODEL INCLUSION: 2.03 (1.76, 2.35). Evidence of association with SGA births from observational studies.

Zinc deficiency

Risk: no observational studies found.

Intervention: The 2021 Cochrane review found that zinc supplement during pregnancy did not reduce the risk of SGA compare to placebo (RR=1.02, 95% C.I. 0.92 to 1.12; 9 studies, 5330 participants, moderate-certainty evidence) [71]. SGA was defined as birthweight less than 10th centile for gestational age. No subgroup analysis for low zinc at baseline was conducted because very few studies used normal zinc population. Taking the inverse of the treatment effect, the risk estimate of SGA birth with zinc deficiency is RR=0.98 (0.89-1.09). The older Cochrane review of RCTs of zinc supplementation versus no zinc (with/without placebo) among pregnant women with “low zinc or nutrition” was published in 2015.[72] The review demonstrated a treatment effect of RR=1.03 (0.95 – 1.12). [72] The “low zinc or nutrition” status was assumed for pregnant women from low-income groups or zinc status from previous records.

Overall quality of risk estimate: High. The Cochrane review deemed the overall quality of the 9 randomized control trials (with 5330 participants) included of “moderate” quality due to design

limitations. Nine studies included both low-and middle-income countries and high-income countries, making the results more generalizable.

MODEL INCLUSION: Not included. Sufficient evidence of no risk for SGA birth based on randomized control trials.

Anemia

Risk: A 2019 systematic review examined the association of maternal Hb concentration with a range of maternal and infant health outcomes. [73] SGA was defined as birth weight below the 10th percentile for gestational age or as defined by study authors. A total of 95 studies met the criteria for inclusion in the meta-analysis. Included studies adjusted for one or more confounders. Maternal age, education level, body mass index, and parity were the variables that were commonly adjusted in the studies. For small for gestational age (N=21), low maternal Hb at any time during pregnancy were associated with higher risk: ≤110 g/L adjusted OR=1.08 (1.00-1.18). [73] The reference group from individual studies varied: some used a range (e.g., 110-119 g/L) and others a cut-off (>110g/L). The category ≤110g/L was cumulative, which included studies using the ≤110 g/L cut off or any subset where all Hb values were ≤110 g/L. The association was stronger with lower cutoffs: ≤70 g/L OR=1.29 (1.15-1.43); ≤80 g/L OR=1.39 (1.22-1.60); ≤90 g/L OR=1.32 (1.20-1.44); ≤100 g/L OR=1.13 (1.01-1.27). [73] Low maternal Hb (≤110g/L) during preconception was also associate with higher risk of SGA: OR=1.79 (1.39-2.31) [73]. Since the paper reported risk estimates in OR and did not have prevalence estimate of low maternal Hb (<110g/L), we used WHO's global prevalence estimate of anemia in pregnant women (41.8%) to convert the OR=1.13 (1.01-1.27) into RR=1.07 (1.0-1.05).

A 2016 systematic review and meta-analysis explored the impact of maternal anemia on birth outcomes in low-and middle-income countries. [74] One of the outcomes was SGA birth (birthweight below the sex-specific 10th percentile of the gestational age). Anemia was defined based on WHO standard thresholds (<10-11g hemoglobin/dL or <30-34% hematocrit). Comparing to nonanemic group, anemia during the first or second trimester was not associated with SGA. For studies reported adjusted RR, the pooled adjusted RR=1.00 (0.84-1.19); 5 studies. [74]

Since there were severe heterogeneity, various stratified analysis, including study design, confounding factors, and geographic region, were performed, but the conclusion did not change. Trim-and-fill method concluded that there was no missing from literature because of publication or other forms of bias. An older meta-analysis (2013), including 9 studies, did not find a risk relationship between prenatal anemia and SGA (adjusted OR=1.08 [0.90-1.29]). [75]

Intervention: The 2015 Cochrane review assessed the effects of daily oral iron supplementation during pregnancy [76]. The IFA formulation varied between studies. The daily dose of elemental iron ranged between 9mg to 900mg. Daily folic acid ranged from 10ug to 5000ug. On comparing any supplements containing iron folic acid (IFA) to same supplements without IFA, IFA supplement reduced the risk of maternal anemia at term (Hb less than 110g/L): RR=0.34 (0.21-0.54); 3 RCTs; 346 participants; I^2=0%. [76] A 2013 meta-analysis found that iron, with or without folic acid was not associated with SGA; RR=0.85 (0.67-1.08); 8 trials; I2=59%. When the

reverse is taken, RR=1.18 (0.93-1.49). Six higher quality trials reached the same conclusion (RR=0.84 [0.62-1.14]). [75]

Overall quality of risk estimate: High. Evidence from randomized control trials show that IFA was effective at reducing anemia, but not SGA. There was inconsistent evidence from systematic reviews. The systematic review that only included LMICs found no increased risk of SGA due to maternal anemia. The most recent systematic review that looked at both LMICs and high-income countries, found significant risk association between SGA and maternal anemia. Since our study focused on LMICs, we decided to include anemia in the extended model.

MODEL INCLUSION: 1.07 (1.0-1.05). Evidence of association with SGA based on observational studies in the extended model.

Low pre-pregnancy BMI

Risk: A 2016 systematic review assessed the dose-response relationships between maternal anthropometric variables and SGA risk. [77] Nine studies (323,243 participants) included BMI as an exposure. The definition of SGA varied across included studies: below 2 SD relative to the mean weight for GA, below 10th percentile for GA, individualized birth weight ratio on or below 3rd percentile, or unclear. All included studies provide adjusted RRs. The reference group is BMI=21.5. For BMI<12.5kg/m^2, the RR=1.91 (1.48-2.46); For BMI<15.5kg/m^2, the RR=1.51 (1.28-1.79); For BMI<18.5kg/m^2, the RR=1.21 (1.12-1.31); For BMI>24.5kg/m^2, the RR=0.88 (0.81-0.94); For BMI>27.5kg/m^2, the RR=0.81 (0.69-0.94); For BMI>30.5kg/m^2, the RR=0.76 (0.60-0.96). [77] Most of the included studies were conducted in non-LMICs, except one in India and one in Vietnam.

Another 2016 systematic review examined the association between maternal pre-pregnancy BMI and the risk of adverse perinatal outcomes. [78] BMI was defined by the WHO and NIH—underweight ≤18.5 kg m^2, normal weight 18.5–24.9 kg m^2, overweight 25.0–29.9 kg m^2 and obese ≥30 kg m^2. The normal weight level was used as the reference group for the analysis. Crude ORs were used in the meta-analysis. Infants whose mothers were underweight had a significantly higher risk of SGA (crude OR=1.67 [1.49-1.87], 9 studies). [78] SGA was defined as birth weight below the 10th percentile based on sex-specific and parity specific standards of the Netherlands Perinatal Register. Of the 26 studies (893,122 pregnant women) reported data for the four BMI levels, the prevalence of underweight was 8.18%, which could be used in the conversion of the odds ratio to a risk ratio equal to crude RR=1.58 (1.43, 1.75). An older systematic review also found that low pre-pregnancy BMI was associated with an increased risk of IUGR (defined as birth weight <10% for gestational age) based on 4 cohort studies (adjusted RR=1.54 [1.38-1.72]; I^2=36%). [79]

Intervention: Balanced protein energy supplement provides nutritional supplementation with less than 25% of the total energy content comes from protein. For baseline marginal nutrition population, providing balanced protein energy supplement can increase weekly gestational weight gain: MD=20.74 (1.46-40.02); 10 studies; 2,571 participants and reduce risk of SGA: RR=0.68 (0.56-0.84); 6 studies; 3,396 participants; I2=0%. [80] A more recent 2011 systematic

review also showed that providing balanced energy protein supplementation to undernourished mothers reduced the risk of having an SGA infant by 31% (RR=0.69 [0.56-0.85]); 6 studies; 335 participants; I2=0%. [81]

Overall quality of risk estimate: High. There was consistent evidence from meta-analysis of observational studies. And the risk estimates were adjusted for confounders. Although the baseline population were not necessarily underweight, evidence from RCTs showed that balanced protein energy supplement was an effective treatment for SGA related to low BMI.

MODEL INCLUSION: RR=1.21 (1.12-1.31) Consistent evidence of risk from observational studies. The intervention—balanced protein energy supplement is effective at reducing risk of SGA for women with low BMI.

High pre-pregnancy BMI

Risk: The risk relationship between high pre-pregnancy BMI and SGA was assessed in a 2016 systematic review. [78] The same paper included low pre-pregnancy BMI and SGA. Similarly, BMI was defined by the WHO and NIH—underweight ≤18.5 kg/m^2, normal weight 18.5–24.9 kg/m^2, overweight 25.0–29.9 kg/m^2 and obese ≥30 kg/m^2. All pregnant women included did not have pre-existing hypertension or pre-existing diabetes. Crude ORs were used in the meta-analysis. Compared to normal weight, overweight or obese mothers had significantly lower risks of SGA (OR=0.71 [0.66-0.76] or OR=0.88 [0.78-0.99], respectively). [78] Moreover, being overweight or obese were found to be associated with being large for gestational age (OR=1.45 [1.29-1.63] or OR=1.88 [1.67-2.11], respectively). [78] An older systematic review also found increased risk of being large for gestational age associated with overweight or obese (OR=1.53 [1.44-1.63]; OR=2.08 [1.95-2.23], respectively). [82]

Overall quality of risk estimate: Low. Liu et al. included 11 studies to estimate the risk of being overweight. Those eleven studies included both developed and developing countries, but no subgroup analysis was conducted to assess developed or developing countries separately. In addition, the reported OR did not control for other confounding factors. However, there were consistent evidence that high pre-pregnancy BMI were associated with higher risk of large for gestational age.

MODEL INCLUSION: Not included. Sufficient evidence of no risk for SGA birth based on observational studies.

Low gestational weight gain

Risk: A 2018 systematic review included eleven studies that assessed association between gestational weight gain above and below IOM guidelines with risk of SGA. [83] IOM guidelines provided different gestational weight gain range for each BMI category: 12.5-18 kg for underweight women; 11.5-16 kg for normal weight women; 7-11 kg for overweight women; and 5-9 kg for obese women. Five studies defined SGA as birth weight <10th percentile for gestational age and five other studies defined SGA by additionally accounting for sex. Across

the BMI categories, gestational weight gain below guidelines was associated with higher risk for SGA than gestational weight gain within guidelines (OR= 1.53 [95% C.I. = 1.44 to 1.64]; I2= 82.8%; absolute risk difference, 5% [95% C.I.= 4% to 6%]). [83] A total of 1,309,136 pregnant women were included in the study and 23% had gestational weight gain below recommendations. The RR=1.36 (1.31-1.43) was calculated based on the OR and the prevalence of low gestational weight gain among the included studies. Moreover, the association was greater in lower pre-pregnancy BMI (underweight: OR=1.89 [1.67-2.14]; normal weight: OR= 1.63 [1.54-1.71]; overweight: OR=1.34 [1.24-1.44]; obese: OR=1.24 [1.06-1.45]). [83]

Overall quality of risk estimate: Medium. All included studies were observational studies with sample size larger than 500. Most studies were conducted in non-LMICs countries. The pooled odds ratio was a combination of crude or adjusted ORs from individual studies.

MODEL INCLUSION: RR=1.36 (1.31-1.43). Evidence of association with SGA birth based on observational data.

Vitamin A deficiency

Risk: No observational studies found.

Intervention: A systematic review included two randomized control trials conducted in Arica, a total of 627 pregnant women [84]. Among mothers who are HIV positive and prior to anti-retroviral therapy, 5000 IU vitamin A per day + 30 mg carotenoids + 200,000 IU dose of vitamin A at delivery did not reduce the risk of SGA (RR=0.89 [0.68, 1.17], I^2=0%) [84]. On calculating the inverse treatment effect RR=1.12 (0.85-1.47). Control group received a placebo: one placebo not specified, the other group received iron-folate and prophylactic chloroquine phosphate. Participants were not necessarily vitamin A deficient, but about 30% of them had a serum retinol less than 0.7 umol at baseline.

Overall quality of risk estimate: Medium. There was limited evidence on vitamin A deficiency and SGA. Although two RCTs were deemed high quality using GRADE criteria, there were also significant limitations. Both studies were conducted among HIV-infected African women.

MODEL INCLUSION: Not included. Insufficient evidence of risk for SGA birth based on randomized control trials.

Low calcium intake

Risk: No observational studies found.

Intervention: Based on a Cochrane review published in 2014, there was no reduction in SGA due to calcium supplementation (3 trials, 9,026 women: RR=0.85 [0.6, 1.21], I^2=0%) in women who received high-dose calcium supplementation (>1g/day). [85] The estimate applied to all women with baseline low calcium intake (mean intake less then 900mg/day) but was not exclusive to women who were determined to be calcium deficient. The inverse of the treatment

effect is RR=1.18 (0.83-1.67). The same review also found that high-dose calcium supplementation significantly reduced blood pressure with or without proteinuria (12 trials, 15,470 women: RR=0.65 [0.53-0.81]), and pre-eclampsia (13 trials, 15,730 women: RR=0.45 [0[84]1-0.65]). [85] Additionally, the impact of high-dose supplementation on pre-eclampsia risk (8 trials, 10,678 women: RR=0.36 [0.20-0.65]) or high blood pressure risk (7 trials, 10,418 women: RR=0.44 [0.28-0.70]) was greater for women with baseline low calcium diets. [85] The updated Cochrane review published in 2018 included the same studies and reached the same conclusion [86]. Another meta-analysis focusing on LMICs, combined data from two studies, also showed a non-significant reduction in risk of SGA (RR =0.90; 0.59-1.38) with calcium supplementation. [87]

Overall quality of risk estimate: Medium. Three randomized control trials with no heterogeneity did not find a risk relationship between high-dose calcium supplementation and SGA. The evidence on pre-eclampsia also deemed a high quality based on GRADE.

MODEL INCLUSION: Not included. Sufficient evidence of no risk for SGA birth based on randomized control trials.

Vitamin D deficiency

Risk: A 2018 meta-analysis concluded that vitamin D deficiency was associated with a higher risk of SGA (adjusted OR=1.41 [1.14-1.75], I^2=62%). [88] Vitamin D deficiency was defined as a 25-OHD level below 50 nmol/L. SGA was defined as neonates with birth weighs below the 10th percentile for their gestational age but not below 5th percentile. The analysis included 10 studies from 2006 to 2016. The adjusted factors were varied across studies, including pre-pregnancy BMI, smoking during pregnancy, season of blood draw, maternal age, gestation age at blood sampling, marital status, insurance status, smoking in the year before pregnancy, multivitamin use, race, allocation, maternal weight, country of birth. Seven studies reported prevalence of maternal vitamin D deficiency. The average prevalence among those seven studies was 27.7%, which was used to convert adjusted odds ratio to RR=1.27 (1.10-1.45).

Intervention: A systematic review on vitamin D supplementation showed that compare to control group, the risk of SGA was lower in the intervention groups [RR=0.69, 95% C.I. 0.51-0.92; p=0.018]. [89] But the baseline vitamin D status was unknown. An overview of the systematic review about vitamin D supplementation was published in 2019. [90] Systematic reviews of RCTs showed a significant difference on risk of SGA (RR=0.72 [0.52-0.99]; low quality). [90] We chose this study as our risk estimate since it included more recently published paper, had larger sample size and more numbers of RCTs. The inverse of the treatment effect from the review is 1.39 (95% C.I. =1.01-1.92). A systematic review of observational studies showed association between vitamin D level and SGA (RR=1.35 [1.18-1.54] 25(OH) D<50nmol/l; low quality). [90] A randomized, double-blinded, placebo-controlled trial were conducted in Bangladesh from 2014 to 2015. This trial was not included in any systematic reviews. A total of 1300 pregnant women were enrolled mid-pregnancy. The baseline serum 25-hydroxyvitamin D concentration were similar across groups, around 26.6-28.7. Overall, 64% of women had vitamin D deficiency. [91] Small for gestational age was defined as weight-for-age z score that was below the 10th percentile according to neonatal standards established by the INTERGROWTH-

21st Project. There was no difference in number of SGA birth (P=0.38) between intervention group and placebo group. [91]

Overall quality of risk estimate: Medium-High. Meta-analysis of observational studies demonstrated the risk relationship between vitamin D deficiency and SGA. And the risk estimates were adjusted for confounders. The most recent RCT showed null effect of vitamin D on SGA. But given the previous evidence of treatment effect of vitamin D supplementation on SGA were abundant, we decided to include the vitamin D deficiency as a risk factor.

MODEL INCLUSION: RR=1.39 (1.01-1.92) Evidence of association with SGA birth based on both randomized control trials and observational data.

Maternal oxidant level

Risk: A 2015 systematic review found no association between antioxidant level and SGA. [92] The common antioxidants assessed were vitamin E, vitamin C and vitamin A. The antioxidants were reported in different units and standardized mean differences across three or more studies were pooled for each antioxidant, stratified by trimester of measurement. All three studies that measured γ-tocopherol in the second trimester adjusted for total cholesterol, and each reported no difference between SGA cases and controls. To explore the association with SGA, in two of the studies vitamin C was measured in the second trimester while in the other two studies, it was measured in the third trimester. The results showed consistently lower vitamin C levels in SGA cases compared to controls. Three studies found that retinol levels measured in the second trimester were similar among pregnancies resulting in SGA versus appropriate for gestational age (AGA) birth. Studies measured retinol levels at either third trimester or shortly after delivery found no differences between mothers delivered SGA and mothers delivered AGA. The review also found a significantly negative standardized mean difference (SMD) for Vitamin E and Vitamin C between women who developed preeclampsia and those who did not, but there was substantial heterogeneity. Included studies had high heterogeneity. Different study designs were included: prospective cohort studies, nested case-control studies, RCTs, case-control studies and cross-sectional studies. Also, most studies were small with important risk of bias. The outcomes were IUGR, defined by serial ultra-sounds or SGA, defined as birth weight <10th percentile.

Intervention: The 2016 Cochrane review assess the effects of vitamin E supplementation, along or in combination with another separate supplementation. [93] Under random-effects model, vitamin E did not influence risk of SGA: RR=0.98 (0.90-1.06); 8 studies; 10,161 participants; I^2=40.66%. [93] The 2016 Cochrane review evaluate the effect of vitamin C supplementation, along or in combination with other separate supplements [94]. Vitamin C supplementation was not related to SGA: RR=0.98 (0.90-1.06); 9 studies; 10,320 participants; I^2=37.29%. [94] The RCTs were conducted in both high-income countries and LMICs.

Overall quality of risk estimate: Medium. Although the systematic review of the observational studies did not provide a pooled risk estimate, the included observational studies had

inconsistent conclusions. And the negative results from intervention trials agreed with observational studies.

MODEL INCLUSION: Not included. Sufficient evidence of no risk for SGA birth based on observational studies and randomized control trials.

Low iodine intake

Risk: Among a total of 6979 mothers in a UK birth cohort, urinary iodine-to-creatinine (I:Cr) was used as the primary indictor for iodine status. [95] In unadjusted observations, the children of mothers in the lowest I:Cr group tended to weigh less and were more likely to be SGA than whose mothers had higher iodine concentration. In adjusted analyses, mothers at 25th I:Cr percentile had 1.9% (0%-3.7%) higher probability of SGA than mothers at 75th percentile. Another study in the UK used urinary iodine concentration as the primary indicator of maternal iodine status. [96] No association was observed between SGA and mean urinary iodine concentration (adjusted OR=1.09 [1.00-1.20]) or mean urinary iodine-to-creatinine ratio (adjusted OR=1.02 [0.96-2.09]).

Another study in Finnish collected blood samples from pregnant women to analyze serum iodide. [97] In both unadjusted and adjusted models, serum iodide was not associated with odds of having an SGA newborn. Based on nationally representative urinary iodine concentration survey, the greatest proportions of children with inadequate iodine intake are in European (43.9%). [98]

Overall quality of risk estimate: Very Low. Although only individual studies from non-LMICs were available, the findings were consistent across different studies and the estimates were all adjusted for potential confounders.

MODEL INCLUSION: Not included. Insufficient evidence of association with SGA birth based on observational studies due to lack of meta-analysis.

General Health Issues or Morbidity

Pre-eclampsia

Risk: A systematic review and meta-analysis collected data from 29 countries (Africa, Latin America, the Middle East, and Asia) in 2010–11 to estimate the association between the birth of SGA infants and the risk factors. [99] The paper defined SGA as a birthweight below the 10th percentile, AGA (appropriate-for-gestational age) as between the 10th and 90th percentiles. In the analyses of the association between SGA and fresh stillbirths and early neonatal death, the paper adjusted for maternal age, marital status, education, parity, medical conditions during pregnancy such as chronic hypertension, pre-eclampsia/eclampsia, severe anaemia, malaria/dengue and HIV/AIDs at the individual level, and capacity of health facilities at the facility level by four categorized HDI (Human Development Index) groups.
The risk factors of delivering Term SGA infants were significantly higher compared to AGA among women diagnosed with pre-eclampsia/eclampsia (aOR= 2.05; 95% C.I.= 1.88–2.23). [99]

The prevalence of pre-eclampsia/eclampsia in this study population (Term delivery) was 24.5% and converting this adjusted odds ratio to an adjusted risk ratio gave an estimate equal to aRR= 1.63 (1.55-1.71).

A secondary data analysis of 2 multicenter RCTs in the U.S. was conducted to evaluate whether women with preeclampsia in a prior pregnancy are at an increased risk of having a pregnancy complicated by a SGA, even in the absence of recurrent pre-eclampsia. [100] A total of 4052 women were included in the analysis. SGA was defined as birth weight below the 10th percentile of normative birthweights for singletons at the time of the original trials. Pre-eclampsia was defined as hypertension (defined as a SBP of ≥140 mm Hg or a DBP of ≥90 mm Hg on 2 occasions 4 hours apart) plus proteinuria (either ≥300 mg per 24 hours or 2+ or more by dipstick on 2 or more occasions 4 hours apart).

Compared with healthy nulliparas, a history of pre-eclampsia was associated with a significantly increased odds for a SGA infant, even if recurrent preeclampsia did not occur (aOR=1.48, 95% C.I.=1.02–2.17). [100] The analysis adjusted for potential confounding variables but did not mention what the adjusted variables were.

Another study was conducted between 1993 and 1995 in Norway with a sample size of 926. SGA infant was defined as having a birth weight 2 SD or more below the expected birth weight. [101] Adjusted for parity, smoking, weight, repeat preeclampsia. The risk of SGA was four times higher in infants born after preeclampsia than in control pregnancies (RR= 4.2; 95% C.I.= 2.2, 8.0). [101] Among nulliparas, preeclampsia was associated with a nearly threefold higher risk of SGA (RR = 2.8; 1.2, 5.9), and among paras, the risk of SGA was particularly high after recurrent preeclampsia (RR = 12.3; 3.9, 39.2). [101]

Intervention: A Cochrane review assessed the effectiveness and safety of antiplatelet agents for women at risk of developing pre-eclampsia. [102] All randomized trials comparing antiplatelet agents with either placebo or no antiplatelet agent were included. Quasi-random studies were excluded. Participants were pregnant women at risk of developing pre-eclampsia (e.g., those with a history of previous early onset severe disease or with diabetes). Interventions were any comparisons of an antiplatelet agent (such as low-dose aspirin or dipyridamole) with either placebo or no antiplatelet. 59 trials (37,560 women) were included in the study. There is a 17% reduction in the risk of pre-eclampsia associated with the use of antiplatelet agents (46 trials, 32891 women; relative risk (RR) 0.83, 95% C.I.= 0.77 to 0.89). [102] Graphs of the effect size against sample size for each trial have been consistently asymmetric, suggesting that small negative trials may be missing. In this review, most of the small positive trials were published in the 1980s and early 1990s.

- Antiplatelet agents versus placebo or no treatment for the primary prevention of pre-eclampsia and its complications

10% reduction in SGA babies (36 trials, 23638 women, RR= 0.90, 95% C.I.=0.83-0.98), RD =0.87% (-1.57, -0.16). [102] There were no statistically significant differences between treatment and control groups for any other outcomes.

- <u>Antiplatelet agents versus placebo or no treatment for secondary prevention of pre-eclampsia and its complications in women with gestational hypertension</u>

Three trials (344 women) reported whether the baby was SGA. There was no clear difference between the groups (RR= 0.76, 95% C.I.= 0.52- 1.10). [102]

Another systematic review and meta-analysis were conducted where the number of RCT trials included in each review ranged from 1-24, with the number of participants ranging from 10 to 21,426. [103]Antiplatelets given before 16 weeks gestation to women who at risk of pre-eclampsia reduced the risk of SGA (RR= 0.47; 95% C.I.= 0.30–0.74). [103] and progesterone therapy for reduces the risk of preterm birth (RR= 0.64; 95% C.I.= 0.49–0.83). Antiplatelet agents for preventing pre-eclampsia toxemia (at moderate risk) were associated with reduced risk of SGA (RR= 0.91; 95% C.I.= 0.83–0.99). [103]

Another systematic review and meta-analysis reviewed effectiveness of interventions to prevent SGA. There were 834 randomized controlled trials (>668 672 participants), reporting on 45 different interventions. Oral magnesium prior to 25 weeks of gestation was associated with reduced odds of SGA babies (RR=0.70; 95% C.I.= 0.46-0.96). [103]

Overall quality of risk estimate: Medium. The evidence from systematic and meta-analysis used cross-sectional data from 29 countries which had LMICs. The quality of the data, especially birthweight and gestational age was questionable in some countries since errors might occur in dating the pregnancy, especially in countries where gestational age is based on the last menstrual period. Additionally, analyzed facility-based data, therefore adverse perinatal outcomes and maternal medical conditions may have been overestimated because only the most severe cases are presented in higher-level facilities. However, the study adjusted for any potential confounders in the analysis.

MODEL INCLUSION: 1.63 (1.55-1.71). Evidence of association with SGA birth based on observational data and randomized control trials. The intervention—magnesium sulphate is effective at reducing risk of SGA.

Gestational diabetes mellitus

Risk: No observational studies were identified

Intervention: The risk of Gestational Diabetes Mellitus (GDM) was estimated as the inverse of the potential treatment effect. On comparing pregnant women receiving standard care to those who received treatment for GDM, pregnant women who were in the latter category showed no difference in the risk of having SGA births (RR=1.10 [0.81-1.48]). [104] Calculating the inverse of the treatment effect, we yielded an estimated RR=0.91 (0.68-1.23). The study participants had no known preexisting diabetes and the treatment ranged from diet modification, glucose monitoring, to insulin. All participants received diagnostic test for GDM at or after 24 weeks' gestation; however, studies used different the glucose inclusion criteria: screening positive on

the 50-g glucose challenge with non-diagnostic oral glucose tolerance tests or meeting National Diabetes Data Group criteria for a diagnosis of GDM.

Quality of risk of estimate: Low. The estimates were pooled from 4 RCTs, including 2,345 participants. 4 RCTs, with no heterogeneity, were deemed moderate quality using Cochrane risk-of-bias tool. The review also concluded that treating GDM were associated with lower risk of having large-for-gestational age neonates (3RCTs, n=2261; RR=0.56 [0.45-0.69]). Although only 4 RCTs were included and SGA were not a primary outcome, the additional evidence of association between GDM and LGA would enhance the evidence of no association between gestational diabetes and SGA.

MODEL INCLUSION: Not included. Sufficient evidence of no risk for SGA birth based on randomized control trials.

Hypertension

Risk: The primary estimate of risk on SGA babies is from a systematic review and meta-analysis study, which used data from 29 countries (Africa, Latin America, the Middle East, and Asia). [99] The study was conducted between 2010–11 to estimate the association between the birth of SGA infants and the risk factors. The paper defined SGA as a birthweight below the 10th percentile, AGA as between the 10th and 90th percentiles. In the analyses of the association between SGA and fresh stillbirths and early neonatal death, the paper adjusted for maternal age, marital status, education, parity, medical conditions during pregnancy such as pre-eclampsia/eclampsia, severe anaemia, malaria/dengue and HIV/AIDs at the individual level, and capacity of health facilities at the facility level by four categorized HDI (Human Development Index) groups. The adjusted risk of delivering Term SGA infants was not significantly higher compared to AGA (Appropriate-for-Gestational Age) among women with chronic hypertension during pregnancy (aOR: 1.20; 95% C.I.= 0.96-1.49). [99] However, there was a statistically significant association between chronic hypertension during pregnancy and increased risk of Term SGA in unadjusted risk estimate (OR=1.47; $p < 0.001$). [99] The prevalence of term SGA was 13.8% and this estimate was used to convert OR into RR=1.38. The risk of delivering Preterm SGA infants was significantly higher compared to AGA (Appropriate-for-Gestational Age) among women who had chronic hypertension during pregnancy (aOR: 1.68; 95% C.I.= 1.22–2.30). [99]

Intervention: A systematic review and meta-analysis compared beta-blockers with placebo/no beta blocker to compare beta-blockers with methyldopa. [105] When oral beta-blockers were compared with other drugs, methyldopa was most used (13 trials); other comparative drugs were hydralazine (three trials), nicardipine (one trial), and isradipine (one trial). Randomized controlled trials were used and quasi-random designs were excluded. SGA defined as either less than the third, fifth or 10th percentiles when birth weight was corrected for gestational age.

- In Beta-blockers versus placebo or no beta-blocker

12 trials were used to analyze 1346 women to analyze association between SGA and beta-blockers versus placebo or no beta-blocker. Use of Beta-blockers was statistically associated with an increased risk of SGA infants (RR= 1.36, 95% C.I.= 1.02-1.82). Although the result of one

small trial appears to be different, the 95% C.I. is wide and overlaps with those of all the other trials. This trial (Scotland 1990) randomized 33 women with chronic hypertension to atenolol versus placebo early in pregnancy (as in USA 1987a) and had two post-randomization exclusions from the control group for severe hypertension, which may be exaggerating the real effect. Also, if this trial is removed from the analysis, the effect size is essentially unchanged, although the confidence interval just crosses statistical significance (RR= 1.30, 95% C.I.= 0.97 to 1.74; 11 trials, N = 1317 women). [105]

- <u>In Beta-blockers versus other antihypertensive agents</u>

When beta-blockers were compared with any other antihypertensive drug, beta-blockers were associated with a lower risk of small-for-gestational-age infants, and this effect was borderline for statistical significance (RR= 0.69, 95% C.I.= 0.48- 0.99; 7 trials, N = 485 women). [105]
Note: a recent meta-regression analysis of all trials of antihypertensive medication for mild to moderate pregnancy hypertension found an association between treatment-induced fall in mean arterial pressure (by administration of any antihypertensive agent) and an increased risk of SGA infants. [106] This would suggest that if the effect of beta-blockers on fetal growth were real, it might not be specific to beta-blockers.

Another systematic and meta-analysis compared the efficacy and safety of antihypertensive agents in pregnant women with chronic hypertension. [107] Twenty-two studies (14 randomized controlled trials and 8 cohorts) were included, comprising 4464 women. The studies were published between 1976 to 2019, representing developed and developing countries (8 from United Kingdom, Panama, Italy, 3 United States, Egypt, 2 Canada, 2 Israel, South Africa, Japan, Brazil, Argentina). SGA was defined as birthweight <10th percentile for the gestational age. Atenolol was associated with significantly higher risk of SGA compared with placebo (odds ratio= 26.00; 95% confidence interval= 2.61–259.29) when compared with no drug intake. [107]

Overall quality of risk estimate: High. The evidence from systematic and meta-analysis used cross-sectional data from 29 countries which had LMICs. The quality of the data, especially birthweight and gestational age was questionable in some countries since errors might occur in dating the pregnancy, especially in countries where gestational age is based on the last menstrual period. Additionally, analyzed facility-based data, therefore adverse perinatal outcomes and maternal medical conditions may have been overestimated because only the most severe cases are presented in higher-level facilities. However, the study adjusted for any potential confounders in the analysis.

MODEL INCLUSION: RR=1.38 Evidence of association between hypertension and SGA based on observational studies.

Polycystic ovary syndrome

Risk: A 2016 systematic review assessed the association between pregnancy in women with polycystic ovary syndrome (PCOS) and pregnancy complications. [108] 10 studies shown that PCOS in pregnancy was not associated with risk of SGA (RR=1.45 [0.96-2.20] I^2=40%). [108] Individual studies adjusted for different factors, but the information was not presented. Since those

studies shown significant heterogeneity, subgroup analysis was conducted. PCOS in pregnancy was associated with increased risk of SGA in studies with prospective design (RR=1.86 [1.27-2.71]), and pregnancies with pre-BMI greater than 25 (RR= 1.97 [1.13-3.46]). [108]
Overall PCOS in pregnancy was associated with greater risk of gestational diabetes mellitus (RR=2.78 [2.27-3.40] I^2=71.7%), preeclampsia (RR=2.79 [2.29-3.38] I^2=0%), pregnancy-induced hypertension (RR=2.46 [1.95-3.09] I^2=39.3%), preterm delivery, cesarean delivery, miscarriage, hypoglycemia, and perinatal death. [108]

Overall quality of risk estimate: Low. All included studies were observational studies. Newcastle-Ottawa Scale was used to evaluate the quality of the study and all the included studies were deemed high quality. Egger and Begg test shown no evidence of publication bias. Although subgroup analysis found different conclusions, given PCOS were also associated with preeclampsia and pregnancy-induced hypertension, the potential risk posed by PCOS might only be effective as an intermediate factor.

MODEL INCLUSION: Not included. Sufficient evidence of no risk for SGA birth based on observational studies.

Subclinical hypothyroidism

Risk: A 2020 systematic review examined the association between maternal thyroid function and birthweight. [109] Definition of thyroid function test abnormalities were based on cohort-specified percentile for thyroid stimulating hormone (TSH) and free thyroxine (FT4), only including women with negative thyroid peroxidase antibody (TPOAb). Subclinical hypothyroidism was defined as a TSH concentration greater than the 97.5th percentile and a normal FT4 concentration. Isolated hypothyroxinaemia was defined as an FT4 concentration of <2.5th percentile and a normal TSH concentration. Compared with euthyroidism, maternal subclinical hypothyroidism was associated with a higher risk of SGA (OR=1.24 [1.04-1.48]; p=0.015) and isolated hypothyroxinaemia was associated with a lower risk of SGA (OR=0.70 [0.55-0.91]; P=0.0073). [109] When analyzed as a continuous variable, 1 SD higher maternal TSH concentration was associated with higher risk of SGA (OR=1.05 [1.01-1.08]). 1SD higher FT4 concentration was associated with a higher risk of SGA (OR=1.07 [1.04-1.11]). [109] The prevalence of maternal subclinical hypothyroidism was 3.1% among 48,145 women included in the systematic review. The RR (1.23 [1.04-1.46]) can be calculated from the OR and the prevalence. Subclinical hyperthyroidism, overt hyperthyroidism, TPOAb positivity, and TgAb positivity were not associated with SGA. An older systematic review also found the similar finding that subclinical hypothyroidism increased the risk of SGA (crude OR=1.54 [1.06-2.25]). [110]

Overall quality of risk estimates: Medium. The analyses adjusted for maternal age, BMI, ethnicity, smoking, parity, gestational at blood sampling, fetal sex, and gestational age at birth (the latter two for birthweight only). Only singleton pregnancies were included. Participants who had pre-existing thyroid disease or thyroid-interfering medication usage, or in-vitro fertilization treatment were excluded. SGA was defined as a standardized birthweight of less than the tenth cohort-specific percentile. The studies included in the paper were conducted in

both developed countries and developing countries. In subsequent stratified analysis, the risk estimate of the association between subclinical hypothyroidism was larger when the TSH and FT4 concentration were measured at second or third trimester than in the first trimester.

MODEL INCLUSION: RR=1.23 (1.04-1.46) Evidence of association with SGA birth from observational studies

Anxiety disorder

Risk: The 2018 systematic review analyzed available data on the association between exposure to antenatal anxiety and the risk of various perinatal outcomes. [111] Maternal anxiety required either a clinical diagnosis of any anxiety disorder or use of a validated self-report anxiety measure with a cut off score for high anxiety. 29 studies were included in the meta-analysis; 16 were above a quality threshold and 13 were below. SGA was significantly associated with maternal anxiety (7 studies, adjusted OR=1.48; 95% C.I.= 1.26-1.74; I^2=0%). [111] Studies included in the paper had different definitions for SGA. For the subgroup (n=3, conducted in Taiwan, France, Ireland), which defined SGA as <10th percentile, adjusted OR=1.46 (1.21-1.75). [111] Commonly adjusted confounders in the studies were age, marital status, SES, smoking habits, BMI, and maternal education. The prevalence of anxiety among the three studies was 17.7%, which can be used to convert OR to RR=1.35 (1.17-1.54).

Intervention: Based on 4 studies, exposure to benzodiazepine (BZD) was not found to be associated with SGA (2279 exposed and 1,324,081 unexposed; OR=1.34 [0.97-1.86]; I^2=46%). [112] Acceptable assessment of BZD exposure included either filing a prescription for a BZD or notation of BZD use in maternal/clinical chart. The systematic assessment of quality in observational research, adapted from the Downs and Black checklist and the Newcastle-Ottawa Scale, was used to assess article quality. Based on the visual inspection of the funnel plot, SGA appeared to be positive for slight publication bias. The definition of SGA not specified. Not all studies reported adjusted estimates. When adjusted estimates were not provided in the published data, crude OR were calculated. Most studies were conducted in Europe and North America.

Overall quality of risk estimate: Medium-Low. The evidence from meta-analysis of observational studies was adjusted for potential confounders and showed risk relationship. The evidence was from non-LMICs. The common drug for anxiety disorder showed no impact on SGA.

MODEL INCLUSION: RR=1.35 (1.17-1.54) Evidence of association with SGA from observational studies.

Depression

Risk: A 2018 meta-analysis explored the impact of SSRIs use for prenatal depression on LBW and SGA. [113] Ten studies examined the association between SSRIs use during maternal depression and SGA. Majority of the studies had mixed SSRI usage. SSRIs use for maternal depression increased the risk of SGA (RR=1.45 [1.18-1.76]; I^2=66.6%). [113] Subgroup analysis by

study design did not change the conclusion. For cohort studies only: RR=1.46 (1.18-1.80). For prospective cohort studies only: RR=2.90 (1.58-5.29). For retrospective cohort study only: RR=1.32 (1.09-1.61). All the RR estimates were unadjusted values. There was no definition of SGA.

Overall quality of risk estimate: Very low. The common antidepressants were found to be associated with higher risk of SGA. But the results did not consider confounding factors and there was no clear definition of SGA. Depression was not a public health concern in LMICs, therefore we assumed low usage of SSRIs in LMICs.

MODEL INCLUSION: Not Included. Insufficient evidence from observational studies of the intervention to demonstrate association with SGA in LMICs.

Inflammatory bowel disease

Risk: A 2015 systematic review estimated the risk of adverse pregnancy outcomes in women with inflammatory bowel disease. [114] The diagnosis of IBD were either based on charts, ICD codes, or self-report. If available, adjusted ORs were pooled, otherwise unadjusted ORs were calculated. Thirteen studies reported on the incidence of SGA birth. The pooled ORs was 1.36 (1.16-1.60); I2=56%. [114] The comparison group were women without the disease. Among the thirteen studies, the prevalence of IBD was 0.28% (11,635 women with IBD and 4,132,914 controls). With the prevalence, ORs can be convert to RR=1.36 (1.16-1.60), indicating IBD increased the risk of SGA. The systematic review included studies conducted over time (1989-2012). However, there were lack of inclusion criteria, for example, studies were included irrespective of patient's disease severity and treatment. Authors developed their own study validation scoring system based on the Newcastle Ottawa scale.

Overall quality of risk estimate: Low. There was evidence from meta-analysis of observational studies; however, not all the risk estimates were not adjusted for potential confounders.

MODEL INCLUSION: RR=1.36 (1.16-1.60). Evidence of association with SGA from observational studies.

Migraine

Risk: a 2019 systematic review aimed to estimate the association between a history of migraine and adverse birth outcomes. [115] Compared to pregnant women without a history of migraine, pregnant women with migraine did not have a higher risk of SGA (unadjusted OR=1.06 [0.98-1.15]; adjusted OR=1.06 [0.99-1.14]). [115] SGA was defined as birth weight below the 10th percentile. Prospective and retrospective cohorts and case-control studies were included. The pooled OR were based on two studies with a total of 30,149 women. In the adjusted analysis, one study adjusted for maternal age, family history of hypertension, smoking habits. The other study adjusted for maternal age, the year of delivery, hypertension, diabetes, hyperlipidemia, coronary heart disease, infant gender, parity, highest maternal education level, parental age difference, mothers' marital status and family monthly income. Another national wide

population-based cohort study in Denmark also did not find association between migraine and SGA (adjusted prevalence ratio=0.94 [0.88-0.99]) [116].

Overall quality of risk estimate: Low. There was evidence of no risk from meta-analysis of observational studies. And the conclusion remained after controlling for confounders.

MODEL INCLUSION: Not included. Sufficient evidence of no risk for SGA birth based on observational studies.

Uterine, Placental, and Cervical Factors

Endometriosis and adenomyosis

Risk: The risk estimate is from a systematic review and meta-analysis of 21 observational studies which were published from 2003 to 2017 (n=2,517,516 women). [117] A total of 13 cohort studies were analyzed to explore association between endometriosis and SGA (2= Denmark, 2=Italy, 2=Japan, 2=Finland, Australia, Sweden, China, US, and Italy and Spain), and 3 studies were used to explore association between adenomyosis and SGA (1=Italy, 2=Japan). SGA was defined as birthweight < 10th centile for gestational age. Each study adjusted for demographic and maternal factors. In most of the studies, diagnosis of endometriosis was made histologically after surgery.

Women with endometriosis had an increased odds of SGA infant (aOR 1.26, 95% C.I. 1.04–1.54); $I^2 = 72\%$, $p < 0.0001$. [117] Since the paper did not have prevalence, we calculated the estimates based on the data provided, which was 0.02 (24700/1033744). This was used to convert the adjusted odds ratio to an estimated adjusted RR= 1.25 (95% C.I. 1.03–1.54). Women with adenomyosis had an even higher odds of SGA infant (OR =3.23, 95% C.I. 1.71–6.09). $I^2 = 4\%$, $p = 0.35$. [117] We calculated the estimates based on the data provided, which was 0.12 (115/974). This was used to convert the odds ratio to an estimated RR=2.55 (95% C.I. 1.58–3.78).

For endometriosis

Overall quality of risk estimate: Low. The evidence from systematic and meta-analysis used observational studies conducted in non-LMIC countries. The study adjusted for potential confounding factors but had high heterogeneity.

MODEL INCLUSION: 1.25 (1.03–1.54). Evidence of association with SGA birth based on observational studies.

For adenomyosis

Overall quality of risk estimate: Low. The evidence from systematic and meta-analysis used observational studies conducted in non-LMIC countries. The study did not adjust for potential confounding factors.

MODEL INCLUSION: 2.55 (1.58–3.78). Evidence of association with SGA birth based on observational studies.

Early vaginal bleeding

Risk: An observational study was conducted in 1988 in the U.S. with a sample size of 7658 women. [118]. SGA was defined as below the 10th percentile of birth weight for gestational age, using US national standards specific to race, infant sex, and parity. Vaginal bleeding was considered light when the women did not need hospital care and heavy when she was admitted to hospital. The analysis adjusted for maternal age, education, prenatal care, and cigarette smoking during pregnancy. Less severe vaginal bleeding during pregnancy was not associated to SGA births compared to those without vaginal bleeding (aOR= 0.9; 95% C.I. = 0.7-1.3). [118] Similarly, there was no association between women with more severe vaginal bleeding during pregnancy and SGA births (aOR= 1.3 (95% C.I. = 0.9-2.0). [118]

A prospective one-year birth cohort study of 807 women was conducted in Finland (1985–1986). [119] SGA definition was baby whose weight is below two standard deviations (<X −2 SD). Vaginal bleeding was considered light when the women did not need hospital care and heavy when she was admitted to hospital. Odds ratios were adjusted for previous low birth weight; previous miscarriages; infertility examined and/or treated; contraception method (none or barrier methods/OC/IUCD/ IUCD *in situ* at the beginning of actual pregnancy); maternal age (<19 years or ≥35 years); parity (0-parous or ≥5-parous); previous preterm birth; and previous stillbirths and/or perinatal mortality (<7 days). Bleeding in 2nd trimester of pregnancy was statistically associated with an increased risk of SGA aOR=2.5 (95% C.I.=1.3–4.9). [119] Light bleeding or heavy bleeding during pregnancy was not statistically associated with SGA compared to those without vaginal bleeding (Light bleeding aOR=1.5; 95% C.I.= 0.9-2.5); (Heavy bleeding aOR=1.9; 95% C.I.= 0.9-4.2). [119] Although the point estimates are relatively large, the C.I. overlaps one because of sample size.

Another prospective birth cohort study of 3531 women was conducted in the U.S to explore the association (1975-1985). [120] Light bleeding was characterized as spotting or slight, and heavy bleeding was characterized as moderate or heavy. Adjusted for maternal age, race, place of birth, pre-pregnancy weight, phase of study entrance, sex of baby, length of gestation at interview, cigarette smoking, parity, induced and spontaneous abortions, working during pregnancy, and gynecologic conditions. Light bleeding during pregnancy was not statistically associated with SGA compared to those without vaginal bleeding (aOR=0.8; 95% C.I.= 0.5-1.5). [120] Heavy bleeding during pregnancy was not statistically associated with SGA compared to those without vaginal bleeding (aOR=1.3; 95% C.I.= 0.5-3.4). [120]

Overall quality of risk estimate: Very low. The risk estimates were from three individual studies conducted in non-LMIC setting. The studies adjusted for potential confounders in the analysis.

MODEL INCLUSION: Not included. In sufficient evidence of association between early vaginal bleeding and SGA due to lack of meta-analysis and lack of evidence in LMICs.

Placenta previa

Risk: The estimate is from a systematic and meta-analysis of 13 studies, where the studies were conducted between 1991 to 2012 (No previa=1,582,651 and previa= 10,575). [121] The

observational studies were conducted in middle income and non-LMIC countries (5 from US, 4 from Israel, and 1 from Finland, Denmark, China, and Turkey). Pregnancies with placenta previa were associated with a mild increase in the risk of IUGR/SGA, with a pooled OR=1.19 (95% C.I. 1.10–1.27). [121] IUGR and SGA were used interchangeably but the definition was the same, i.e., below the 10th percentile for gestational age. Statistical heterogeneity was high with an I^2=94%. The analysis did not adjust for confounders.

Another systematic and meta-analysis of 5 studies was conducted from 1988 to 2010 with a large sample size (N= 1,137,103). [122] The observational studies were conducted in non-LMIC countries (2 from U.S., and 1 each from Israel, Australia, and Turkey). Risk of SGA in those with placenta previa was statistically not higher than those without placenta previa (RR= 1.01; 95% C.I.= 0.62- 1.65). [122]

Overall quality of risk estimate: Very low. The evidence from systematic and meta-analysis based on studies conducted in both the LMIC and non-LMIC countries. The risk estimates did not adjust for any potential confounders in the analysis and had high heterogeneity.

MODEL INCLUSION: Not included. Insufficient evidence of association between placenta previa and SGA based on observational studies.

HPV vaccine

Risk: HPV vaccine exposed pregnancies were not associated with higher risk of SGA (RR=0.96 [0.86-1.07], I2= 0%). [123] Four studies (n=45,408; 9867 exposed and 35,541 control) assessed SGA in HPV vaccine exposed/unexposed pregnancies. Two defined SGA as birthweight less than the 10th percentile for gestational age, one defined by additionally accounting for sex, and one did not specify the definition. Two prophylactic vaccines (bivalent and quadrivalent) were evaluated. The subgroup analysis by vaccine types did not change the conclusion. Most included studies were RCTs and retrospective cohort studies. The Newcastle-Ottawa quality assessment scale was used to assess the risk of bias cohort studies. And the methodological quality of RCTs were assessed using the Jadad Scale. The RRs were not adjusted because most data were from studies that were conducted for regulatory purposes by the manufacturers of the HPV vaccine.

Overall quality of risk estimate: Medium. There was evidence from meta-analysis of observational studies. But the risk estimate was unadjusted; therefore, the estimate might be biased.

MODEL INCLUSION: Not included. Sufficient evidence of no risk for SGA birth based on observational studies.

Fetal Characteristics

Fetal sex

Risk: An observational study was conducted in the UK between 2016 and 2017. A total of 4112 women were enrolled but the study analyzed only 412 women. "Ultrasound SGA" was defined as "SGA at birth" as birthweight < 10th centile adjusted for sex. Among 412 cases of ultrasound SGA, female was statistically more likely to be SGA fetuses than male (OR=1.50; 95% C.I.=1.22–1.85; *p* <.001). [124]

Another observational study of 998 newborns conducted in Brazil in 2014 found that female sex was 1.69-fold more likely to be SGA than male sex (p=0.002). SGA was defined as <10th percentile. The risk estimate did not adjust for potential confounders in the analysis. [125] Similarly, another study is based on a randomized, double-blind, placebo-controlled trial of neonatal vitamin A supplementation conducted in Tanzania between 2010 and 2013 (N=19269 women). [126] SGA defined as birth weight <10th percentile for gestation age and sex using INTERGROWTH standards. Male child had a statistically significant increased risk of SGA compared with female (RR=1.10; 95% C.I.= 1.03–1.17). [126]

Overall quality of risk estimate: Very low. The risk estimate did not adjust for potential confounders in the analysis and the study based on a single observational study conducted non-LMIC country.

MODEL INCLUSION: Not included. Insufficient evidence of association with SGA based on observational studies.

Twin pregnancy

Risk: The estimate of risk is from a systematic and meta-analysis of studies conducted in 23 low- and middle-income countries. The study had a sample size of 276,187 singleton births and 6,476 twin births (twin births representing 1·2% of births in the data set). [127] The study reported that 38% of the twin pregnancies were SGA birth, compared to 9.7% of single pregnancies (Chi-squared p-value <0.001). The incidence estimates were not adjusted. Using these estimates, we calculated an unadjusted RR= 3.98. [127]

Another study performed a retrospective cohort analysis of prospectively recorded maternity data from 1995 to 1997 in the US, with a sample size of 16120 women. [128] Twin pregnancies were categorized into two groups: twins with one fetus affected by a non-chromosomal, structural anomaly (exposed group) and twins without structural anomaly in either fetus (nonexposed group). SGA defined as birth weight <10th percentile for expected gestational age in different sex, according to birth weight distribution of twins born in the United States during 1995 and 1997. Logistic regression analysis adjusted for adjusted for maternal age, race, level of education, marital status, smoking during pregnancy, and initiating time of prenatal care. Compared with the control subject, the presence of an anomalous cotwin significantly increased the risk of SGA (aOR 1.21; 95% C.I. 1.07–1.36). [128] It was a similar association in case of PTB at <32 weeks of gestation (aOR 1.85; 95% C.I., 1.65–2.07).

Overall quality of risk estimate: Medium. The evidence from systematic and meta-analysis is based on observational studies conducted in LMIC countries. However, there was no adjustment for potential confounders in the analysis.

MODEL INCLUSION: RR=3.98. Evidence of association with SGA birth based on observational studies.

Vanishing twin

Risk: The 2017 systematic review explored the actual obstetric outcome of vanishing twin syndrome. [129] The population included women who had singleton delivery after in vitro fertilization-embryo transfer and freezing-thawing embryo transfer. There was no overall definition for SGA. Two case-control studies (one in Austria, one in Israel) assessed SGA as one of the outcomes. Vanishing twin syndrome was not associated with small for gestational age (RR=1.29 [0.52-3.18]) [129]; heterogeneity 72%. This paper did not adjust for any variables.

An observational study was conducted during a 7-year period (1995 to 2001) in Denmark with a sample size of 8542 pregnancies. [130] The study was conducted to assess the effect of a vanishing twin on the risk of being SGA in IVF (In vitro fertilization) singletons and to evaluate the effect of gestational age at the time of vanishing on the risk of being SGA. SGA is birthweight below the lower 10th percentile for each gestational age. Vanished twin is an empty sac or intrauterine fetal demise in the first, second or third trimester. The risk of SGA infants was 50% higher in singleton pregnancies with a vanishing twin than in the primary singleton cohort (OR: 1.50, 95% C.I.= 1.03–2.20). [130]The study did not adjust for confounding factors. Adjusting for maternal age, parity, child gender, cohort (singleton/survivors), late gestational age at vanishing was significant predictor of being SGA compared to early gestational age at the time of vanishing (aOR: 2.08, 95% C.I.: 1.00–4.35). [130] This indicates that the risk of SGA is increased with increasing gestational age at the time of vanishing.

A case-control study in Austria was conducted between 1999 and 2005. A total of 794 singleton deliveries after IVF/intracytoplasmic sperm injection were enrolled, but 46 were included as survivors of vanishing twin syndrome (study group) and 92 as matched singletons (control group). The frequency of being SGA (32.6% vs. 16.3%; p value=0.03) was significantly lower in the singleton pregnancy groups than in those of vanishing twin. [131]

Overall quality of risk estimate: Very low. There were inconsistent conclusions from observational studies conducted in High Income Countries (HICs). All the risk estimates were crude and no adjustment for confounders. In addition, vanishing twin syndrome occurs after in vitro fertilization, which is not likely a concern in LMICs.

MODEL INCLUSION: Not included. Insufficient evidence of association with SGA birth due to frequent conflicting conclusion and vanishing twin was not a potential risk factor in LMICs.

Section 4: Sources of Prevalence Estimates and Quality

This section contains information of the prevalence of the 27 risk factors included in the one of the models. The source of the prevalence estimate is listed first; second is the quality of the source; third is an explanation of process to correcting the prevalence estimates for treatment, if applicable.

Malaria

Prevalence: To calculate the prevalence estimate, we used country-level total malaria cases, irrespective of gender and ages from the 'World Malaria Report [132] and used total country population from the UN population database [133]. Country level data on malaria cases have corresponding 95% confidence intervals. The data from these two sources were used calculate country level incidence estimates of malaria.

Quality: Medium. Country-specific data collected.

Treatment: The prevalence of malaria was adjusted for coverage of prevention of malaria during pregnancy, including percent of pregnant women receiving 2+ doses of Sp/Fansidar or seeping under an insecticide treated net. The coverage data was extracted from LiST.

HIV

Prevalence: We used the UNAIDS's country-specific estimates on the prevalence of women with HIV status from 2019. [134] Country level data have corresponding 95% confidence intervals. Global, regional level estimates on the prevalence of HIV among women adults were also provided. If countries used <0.1 estimates for prevalence and 95% C.I., we used 0.01 as prevalence estimate, 0.001 as lower 95% C.I.., and 0.05 as upper 95% C.I.
To get prevalence estimates for the countries with missing HIV prevalence estimate, we calculated median of the upper bound estimates for each 7 WHO regions. These 7 median values were used to get the prevalence estimates for the countries based on the WHO region it belonged to. Since there were no 95% C.I. estimates, median of the prevalence estimates the WHO was used for all the countries.
Quality: High. Global, regional, country-specific data collected by UNAIDS in 2019, irrespective of the pregnancy status.

Chlamydia

Prevalence: The prevalence estimates for chlamydia are IHME/GDBx [135], which has estimated global, regional, and country level age standardized prevalence of chlamydia, 2017. International classification of disease ICD-9 and ICD-10 were used to define chlamydia infection. Both symptomatic and asymptomatic cases were included.
Quality: High. Has global, country, and regional-specific data in WRA, irrespective of the pregnancy status.
Treatment: Not adjusted for treatment since there is lack of systematic review/ meta-analysis or Cochrane review on treatment for chlamydia and SGA.

Trichomonas vaginalis

Prevalence: The prevalence estimates for chlamydia are IHME/GDBx [135], which has estimated global, regional, and country level age standardized prevalence of Trichomoniasis, 2017. International classification of disease ICD-9 and ICD-10 were used to define Trichomonas vaginalis. Both symptomatic and asymptomatic cases were included.

Quality: High. Has global, country, and regional-specific data in WRA, irrespective of the pregnancy status.

Heavy physical workload during pregnancy

Prevalence: The prevalence is based on labor force participation rate of women above 15 years collected by the World Bank and International Labor Organization (ILO) from 2016 or 2017. [136] [137] The data is from more than 100 countries and has provided global, regional, and country level data. The labor force participation rate is the proportion of the population ages 15 and older that is economically active; women who are either employed (including part-time employment starting from one hour a week) or unemployed (including anyone looking for job, even if it is for the first time). Estimates do not have 95% confidence intervals.
Quality: Medium-low. Regional and country level data only in WRA, irrespective of the pregnancy status.

Secondhand smoking

Prevalence: The study was part of overall Global Burden of Disease Study 2015. [138] A daily smoker was defined as an individual using any type of smoked tobacco product daily. Age-standardized prevalence of daily smoking for male in 2015 were available.
Quality: Medium. Country-specific data were available, but the prevalence included all women.

Indoor air pollution

Prevalence: The prevalence of indoor air pollution is calculated based on three datasets: access to clean fuels and technologies for cooking (% of population) [139]; Deaths-household air pollution from solid fuels-Both sex: Age standardized in 2019 (rate) [135]; total population in 2020 [140]. Prevalence of indoor air pollution is calculated as (death-household air pollution from solid fuels/total population)-% access to clean fuels and technologies for cooking.
Quality: Medium. Country-specific data for the general population.

Ambient air particular matter

Prevalence: The prevalence estimates for ambient air particulate matter (PM2.5) are from an analysis for the Global Burden of Disease study 2015. [141] The authors estimated the “summary exposure value” SEV, which was age standardized and estimated for women in every country. The authors state that the SEV represents the relative-risk weight prevalence of the exposure. Furthermore, the exposure estimates for PM2.5 had a “data representativeness index” (DRI) of 100% because there were data points on the exposure for all countries and all years. The estimates for exposure to ambient air pollution were calculated using the WHO air pollution

data base and satellite-based estimates. Exposure to ambient air pollution is defined as the population-weighted annual average mass concentration of particles with an aerodynamic diameter less than 2.5 micrometers (PM2.5) in a cubic meter of air.
Quality: Medium. Modeled country-specific data in WRA, irrespective of the pregnancy status.

Maternal age and parity

Prevalence: The prevalence for the maternal age and parity categories comes from the Family Planning module of the LiST data base, which compiles data from national surveys, demographic health surveys and multiple indicator cluster surveys.
Quality: High. Country specific estimates from population-representative surveys.

Birth interval

Prevalence: The country specific estimates for prevalence of short birth interval (<18 months and 18-24 months) are from the LiST database, which has compiled data from multiple surveys (DHS, MICS, Fertility and Family Survey- FFS and national surveys). The prevalence of birth interval >60m is obtained from STAT complier [142], containing data from DHS surveys. Some of the prevalence of birth interval >60m is calculated based on the logistic relationship between total fertility and >60m birth interval.
Quality: High. Country-specific estimates available.

Smoking during pregnancy

Prevalence: The study was part of overall Global Burden of Disease Study 2015. [138] A daily smoker was defined as an individual using any type of smoked tobacco product daily. Age-standardized prevalence of daily smoking for female in 2015 were available.
Quality: Medium. Country-specific data were available, but the prevalence included all women.

Alcohol consumption

Prevalence: The estimates for prevalence of alcohol consumption is from a Lancet paper that has regional (Africa, Eastern Mediterranean, European, Americas, South Asia) and country level estimates of prevalence of alcohol consumption during pregnancy. [143] Data has 95% confidence intervals. The estimates were based on the systematic review and meta-analysis of studies. Studies on the prevalence of alcohol use during pregnancy were omitted if they used a sample population not generalizable to the general population of the respective country. For countries with one or no empirical studies, the predicted prevalence of alcohol use during pregnancy using fractional response regression modelling and prevalence of Fetal Alcohol Syndrome using a quotient of the average number of women who consumed alcohol during pregnancy per one case of FAS. Some of the countries made estimate based on a meta-analysis of the current literature. Since the alcohol consumption >2 or more drinks per day is associated with an increased risk of SGA, we also adjusted the prevalence using regional prevalence of heavy episodic drinking among drinkers 15+ years. [144]
Quality: Medium-High. Modelled country-specific data available.

Short maternal height

Prevalence: The estimate for prevalence of women with short maternal height (<145 cm) is from the ICF website that has compiled information from the Demographic and Health Surveys (DHS). For countries with a DHS survey between 2006-2016, country-specific estimates were used. For countries without a recent DHS, a regional estimate was calculated using the average of the 2006-2016 survey data from country estimates in the region. 95% confidence intervals were not given for the estimates.
Quality: Medium overall. High for countries with country-specific data, low for those with regional estimates.

Anemia

Prevalence: The estimates for prevalence of anemia among pregnant women are from LiST database. The original source is from WHO estimates of anemia prevalence 1996-2011 [145]. We used the most recent data i.e., 2011.
Quality: High. Country-specific estimates available for most countries.

Low pre-pregnancy BMI (BMI<18.5)

Prevalence: The estimates for prevalence of lower maternal BMI (<18·5) are retrieved from the LiST database. The original source is from a systematic analysis of world-wide trends in BMI from 2000-2014 [146]. We used the most recent data from 2013 or 2014. 95% confidence intervals were not given for the estimates, therefore a confidence interval with a width of 10% was constructed around the point estimate.
Quality: High. Country-specific estimates available for most countries.

Low gestational weight gain (below IOM guidelines)

Prevalence: A systematic review included 23 cohort studies, reporting data on more than 1 million women. [147] Ten study from US, five from Western Europe, and eight from East Asia (China, Korea, Taiwan, and Japan). For gestational weight gain below guidelines, prevalence was 21%, 18%, and 31% in the USA, Europe and Asia, respectively. In another systematic review, 16 studies, conducted in sub-Saharan Africa, provided proportion of inadequate gestational weight gain according to the IOM. Nine of the 16 studies reported the percentage of women with inadequate GWG as >50%. The prevalence of inadequate GWG were higher among pre-pregnancy low BMI women. Some country-specific data were reported, but for some countries, there were two data from two individual studies.
For other Sub-Saharan African countries, the average of the reported prevalence from other sub-Saharan countries were used. [148] For all the countries in Asia, the 31% was applied. 18% was applied to all high-income countries in Europe. For all other countries, we used 23%, the average from the three regional estimates. In addition, the systematic review we referred to estimate the RR of low gestational weight gain on SGA had an overall prevalence of 23%.
Quality: Low. Only regional data from non-LMICs or country-specific data from individual studies were available.

Vitamin D deficiency (<50 nmol)

Prevalence: A systematic review of recent literature (2003-2013) on global vitamin D status, including population-based studies and cross-section studies. [149] The cutoff points used were: <30 nmol/l (12 ng/mL); <50 nmol/l (<20 ng/mL) and <75 nmol/l (30 ng/mL). For pregnant and lactating women, a total of 17 studies were found: 2 in America; 6 in Europe; 1 in Africa; 7 in Asia; 1 in Oceania. Another systematic review included 95 publications that reported circulating 25(OH)D concentrations in maternal and newborn population. [150] 88 of them reported Vitamin D status in mother. 25(OH)D <50 nmol were defined as deficiency and <25 nmol were defined as severe deficiency. Vitamin D deficiency during pregnancy was presented in 42–72% of women from the Americas, 18–90% from the European,46% from the Eastern Mediterranean, 66–96% from South-East Asian and 41–97% from the Western Pacific region.

For countries that did not have Vitamin D deficiency, we applied the average of all the available country-specific prevalence.

Quality: Low. Although some country-specific data were available, those were from individual studies, not representative of the total population.

Hypertension

Prevalence: Global, regional, and country level estimates for chronic hypertension prevalence are from the World Health Organization that had mostly used population-based surveys. [151] Most recent year of country-specific estimates were used, where majority of the countries had data that were collected in 2015. It had 95% C.I. estimates but do not exclusively include women during pregnancy. Percent of defined population with raised blood pressure was based on measured blood pressure systolic where systolic blood pressure ≥ 140 OR diastolic blood pressure ≥ 90). If multiple blood pressure readings were taken, first reading per participant was dropped and average of remaining readings was used. WHO provided age standardized estimate and crude estimate of women >18 years; some countries' age standardized estimate was higher than the crude estimate, while in others it was vice-versa. Although in each country there was a difference between these two types of estimates, none of the country had values that had a difference of more than two times the estimate. So, we chose age standardized estimates.

Quality: High. Global, regional, and country level data only in WRA, irrespective of the pregnancy status.

Pre-eclampsia

Prevalence: The prevalence data are from 74 reports with 78 datasets analysis, which reported regional level data for preeclampsia. [152] It used the WHO's Systematic Review of Maternal Mortality and Morbidity Project Protocol as a template for screening, identification and selection of studies to all potentially eligible papers published during the period 2002–2010. For countries without national data, a logistic model was developed to estimate the incidence of HDP using country macroeconomic indicators, variables related to health care, and variables related to population characteristics. There were two values reported, a crude pooled preeclampsia incidence estimates from the available study data and a modeled incidence estimate that populated data for countries without crude data using "country macroeconomic

indicators" that includes a 95% confidence interval. For four of the six WHO regions, the modeled estimate was larger (in some cases, nearly two times as larger) compared to the crude incidence. Due to this difference, the crude data is currently in the model to avoid overestimation. Country estimates were provided for some countries that were included to calculate the regional estimate; however, it did not have 95% C.I.
Quality: Low. Six regional level data available.
Treatment: The prevalence of pre-eclampsia was adjusted for MgSO4 treatment. The coverage of MgSO4 treatment was extracted from LiST, using quality (antenatal care visit coverage) and readiness data (availability of equipment in the health facility). For the special case of missing antenatal care visit coverage in China, the data was calculated from China Health Statistical Yearbook 2018.

Sub-clinical hypothyroidism

Prevalence: Different studies used different reference to defined subclinical hypothyroidism, usually based on cohort-specific centile. The prevalence in large population-based studies in many different countries (Italy, Netherlands, Australia, US, China, UK) has been reported to be 3% to 10%. [153] Within US, National Health and Nutrition Examination Survey (NHANES) III shown that the prevalence was about 2-fold greater in females, increased with age, and was about three times greater in whites than in blacks. But NHANES excluded pregnant women. Another review provided reported prevalence of subclinical hypothyroidism in pregnancy for a few countries, including Texas, USA; multicenter, USA; UK and Italy; Czech Republic; Shenyang, China [154]. Prevalence ranged from 2.2% (multicenter, USA in 2008) to 15.13% (USA in 2012). We used a general prevalence estimate of 3% for all countries because most of the reported prevalence of subclinical hypothyroidism were around 3%. In addition, the systematic review we referred to estimate the RR of subclinical hypothyroidism on SGA had an overall prevalence of 3.1% among 48,145 women.

Quality: Low. Only non-LMICs country specific prevalence is available. An estimated global prevalence is used.

Anxiety

Prevalence: The review identified 13 studies that reported point prevalence data about common mental disorders in pregnant women. [155] In almost all studies, participants were recruited while attending a health facility for antenatal care. The average prevalence is 15.9%: 95% confidence interval, C.I.: 15.0-16.8%. For countries with no country-specific data, the average 15.9% was applied.
Quality: Low. Country-specific data from cohort studies.

Inflammatory bowel disease

Prevalence: Lancet paper conducted systematic review of population-based studies on the incidence (119 studies) or prevalence (69 studies) of IBM from 1990 to 2016. [156] Regional prevalence based on United Nations classification of economic regions was available (North America, Eastern Europe, Northern Europe, Southern Europe, Western Europe, Eastern Asia,

South-eastern Asia, Southern Asia, Western Asia, South America, Oceania, Africa). Prevalence was reported separately for Crohn's disease and Ulcerative colitis, reported as lowest estimate and highest estimate. In the supplemental material, the prevalence of inflammatory bowel disease was available. The lower estimate and higher estimate from different countries in the region were applied as 95% C.I.
Quality: Low. Only regional prevalence was available.

Endometriosis

Prevalence: The prevalence estimates for chlamydia are IHME/GDBx, which has estimated global, regional, and country level age standardized prevalence of endometriosis, 2017. [135] Diagnosis by either ultrasound or pelvic exam was set as the reference category. Study-level covariates for diagnosis by hysterectomy only, pelvic exam only, self-report, and pelvic exam, ultrasound or hysterectomy were included.
Quality: High. Has global, country, and regional-specific data in WRA, irrespective of the pregnancy status.

Adenomyosis

Prevalence: A systematic review and meta-analysis was conducted that included 9 studies representing 6 countries (2 from Asia, 5 from Europe, and 2 from Australia). [157] The observational studies were published between 1998-2013. They have not provided 95% C.I. for the prevalence estimates but have values of women with adenomyosis during pregnancy for each study. Since there were no 95% C.I. estimates, median of the prevalence estimates from the 9 countries were used and the single/same median value was used for all the countries.
Quality: Low. Has information only on data of women with adenomyosis during pregnancy for each study.

Twin pregnancy

Prevalence: Data on incidence of twinning was extracted from birth histories of women aged 15–49 interviewed in 150 DHS surveys conducted between 1987-2010 in 75 LMICs. [158] National twinning rates were computed by dividing the number of twin births by the total number of births and multiplying the outcome by 1000. Country estimates were provided, however, it did not have 95% C.I. Two types of estimates were provided for each country: Natural Twinning rate and Standardized Twinning rate. There was not much difference between these two types of estimates (difference of less than 2.0). So, we chose Standardized Twinning rate since it was standardized for age at birth of the mother.
In order to get prevalence estimates for the countries with missing HIV prevalence estimate, we calculated median of the prevalence estimates for each 7 WHO regions. These 7 median values were used to get the prevalence estimates for the countries based on the WHO region it belonged to. Since there were no 95%C.I. estimates, median of the prevalence estimates the WHO was used for all the countries.
Quality: Medium. Country level data available from 75 LMIC representing Asia, Africa, Latin America, Europe. Regional and global estimates were not provided.

Section 5: Quality Assessment

Supplemental Table 2a. Quality checklist for risk factors

	Checklist		Score
**A**	**Method section**		
1	**Outcome definition: Small-for-Gestational Age**	<10th percentile birthweight for Gestational Age, using standard reference	1
		<10th percentile birthweight for Gestational Age, using unknown/cohort reference	0.5
		Other or Undefined	0
2	**Exposure/covariates definition**	Defined	1
		Some defined while some not or defined differently	0.5
		Not defined	0
3	**Timeline**	This paper was published in the last 5-10 years	1
		This paper was published more than 11 years ago	0
		All the studies included in the paper had data collected in the last 10-15 years	1
		Some of the studies included had data collected in the last 10-15 years	0.5
		Study included had data collected more than 16 years ago	0
4	**Country where the study was conducted**	Sufficient (4-5 studies conducted in Low-Middle Income-Countries)	1
		Some (<4 of studies conducted in Low-Middle-Income Countries)	0.5
		None in LMICs	0
5	**Number of data sets**	Systematic review and meta-analysis (=>5 datasets)	2
		Systematic review and meta-analysis (<5 datasets)	1
		Single studies (but consistent evidence from # of single studies)	0.5
		Only a single observational study	0
6	**Data items**	Large sample size in the research paper (>10,000)	1
		Small sample size in the research paper	0
**B**	**Result section**		
2	**Number of variables adjusted in the model**	Adjusted for relatively sufficient variables (#)	1
		Adjusted for relatively insufficient variables (#)	0.5
		Did not adjust for any variables	0
3	**Risk of bias**		
	**In individual or across studies**	Describe methods used for assessing risk of bias of individual studies (including specification of whether this was done at the study or outcome level), and how this information is to be used in any data synthesis.	1
	**Across studies**	Specify any assessment of risk of bias that may affect the cumulative evidence (e.g., publication bias, selective reporting within studies).	1
4	**Heterogeneity**	Low heterogeneity (0-30%)	2

	Checklist		Score
		Medium heterogeneity (30-60%)	1
		High heterogeneity (>60%)	0.5
		No heterogeneity provided	0
5	**Magnitude of effect**	Statistically significant large magnitude (>=1.10)	1
		**Total**	**14**
	High Quality >10; Medium Quality 8-10; Low Quality <8		

Supplemental Table 2b. Quality checklist for intervention

	Checklist		Score
A	**Method section**		
1	**Outcome definition: Small-for-Gestational Age**	<10th percentile birthweight for Gestational Age, using standard reference	1
		<10th percentile birthweight for Gestational Age, using unknown/cohort reference	0.5
		Other or Undefined	0
2	**Exposure/covariates definition**	Defined	1
		Some defined while some not or defined differently	0.5
		Not defined	0
3	**Timeline**	Published in the last 5-10 years	1
		This paper was published more than 11 years ago	0
		All the studies included in the paper had data collected in the last 10-15 years	1
		Some of the studies included had data collected in the last 10-15 years	0.5
		Study included had data collected more than 16 years ago	0
4	**Country where the study was conducted**	Sufficient (4-5 of studies conducted in Low-Middle-Income Countries)	1
		Some (<4 of studies conducted in Low-Middle-Income Countries)	0.5
		None in LMICs	0
5	**Number of data sets**	Systematic review and meta-analysis (=>5 datasets)	2
		Systematic review and meta-analysis (<5 datasets)	1
		Single studies (but consistent evidence from # of single studies)	0.5
		Only a single RCT/observational study	0
6	**Data items**	Large sample size in the research paper (>10,000)	1
		Small sample size in the research paper	0
7	**Study design**	Both Randomized Controlled Trial and Observational	2
		Only Randomized Controlled Trial	1
		Only Observational	0
B	**Result section**		
1	**Adjusted versus unadjusted estimates**	Adjusted Risk Ratio/Odds Ratio	1

	Checklist		Score
		Some studies adjusted while some unadjusted	0.5
		Did not adjust for any variables	0
2	**Number of variables adjusted in the model**	Adjusted for relatively sufficient variables (#)	1
		Adjusted for relatively insufficient variables (#)	0.5
		Did not adjust for any variables	0
3	**Risk of bias**		
	**In individual or across studies**	Describe methods used for assessing risk of bias of individual studies (including specification of whether this was done at the study or outcome level), and how this information is to be used in any data synthesis.	1
	**Across studies**	Specify any assessment of risk of bias that may affect the cumulative evidence (e.g., publication bias, selective reporting within studies).	1
4	**Heterogeneity**	Low heterogeneity (0-30%)	2
		Medium heterogeneity (30%-60%)	1
		High heterogeneity (>60%)	0.5
		No heterogeneity provided	0
5	**Magnitude of effect**	Large magnitude (>1.x)	1
		**Total**	**17**
	High quality: >13; Medium quality 10-13; Low quality <10		

Supplemental Table 2c. Overall quality of risk estimates

Observational	Randomized Controlled Trial/Intervention Trials	Overall Quality
High	High	High
Medium	High	High
Low	High	High
High	Medium	Medium-High
Medium	Medium	Medium
Low	Medium	Medium-Low
High	Low	Medium
Medium	Low	Medium-Low
Low	Low	Low
High	None	Medium
Medium	None	Low
Low	None	Very Low
None	High	High
None	Medium	Medium
None	Low	Very low

Section 6: Global, Regional, and Country Population Attributable Fractions

Supplemental Table 3. Unadjusted global and regional Population Attributable Fractions

RISK FACTOR	GLOBAL (min-max)	SUB SHARAN AFRICA (min-max)	SOUTH ASIA* (min-max)
**Maternal Infection**			
Malaria	0.53% (0.00%-3.91%)	1.76% (0.00%-3.91%)	0.04% (0.00%-0.05%)
HIV	0.70% (0.01%-17.65%)	2.26% (0.01%-17.65%)	0.07% (0.01%-0.10%)
Chlamydia	0.22% (0.10%-0.62%)	0.30% (0.10%-0.56%)	0.16% (0.12%-0.29%)
Trichomonas vaginalis	1.87% (0.91%-6.10%)	3.24% (1.00%-6.10%)	1.31% (1.04%-1.39%)
**Environmental and other Exposure during Pregnancy**			
Heavy Physical workload during pregnancy	2.85% (0.44%-5.92%)	4.51% (1.10%-5.92%)	2.01% (1.79%-5.70%)
Secondhand smoking	0.56% (0.04%-1.43%)	0.34% (0.13%-1.17%)	0.59% (0.26%-1.17%)
Indoor air pollution	12.97% (0.18%-18.56%)	16.20% (1.52%-18.56%)	12.10% (8.20%-15.26%)
Ambient air pollution	6.56% (1.29%-9.17%)	5.39% (1.80%-8.09%)	7.63% (6.86%-8.68%)
Smoking	2.64% (0.34%-11.46%)	1.92% (0.52%-7.21%)	2.63% (1.53%-9.88%)
Alcohol consumption during pregnancy	0.19% (0.00%-1.95%)	0.35% (0.08%-0.73%)	0.06% (0.00%-0.08%)
**Pregnancy History**			
Maternal age < 18 & Primiparity	2.88% (0.51%-9.18%)	3.92% (1.29%-6.87%)	2.45% (1.51%-9.18%)
Maternal age 18-35 and Primiparity	7.82% (2.45%-11.50%)	4.88% (2.45%-9.22%)	9.30% (6.24%-10.27%)
Birth interval of < 18 months	2.93% (0.88%-5.58%)	2.47% (0.88%-4.16%)	3.23% (1.17%-5.58%)
Birth interval of 18- <24 months	1.99% (0.73%-3.20%)	2.09% (0.82%-3.08%)	2.00% (0.82%-2.81%)
Birth interval of over 60 months	2.25% (0.03%-7.45%)	1.44% (0.03%-6.07%)	2.19% (1.35%-6.20%)
**Maternal Nutrition Status**			
Short height	6.81% (0.21%-20.67%)	2.34% (0.21%-6.90%)	9.31% (4.90%-11.49%)
Anemia	3.27% (1.50%-4.31%)	3.19% (1.71%-4.23%)	3.37% (2.29%-3.54%)
Low pre-pregnancy Body Mass Index	3.75% (0.00%-5.02%)	2.51% (0.00%-4.01%)	4.72% (2.62%-5.02%)
Low gestational weight gain	11.96% (6.73%-21.66%)	16.27% (6.73%-21.66%)	10.13% (10.13%-10.13%)
Vitamin D Deficiency	20.01% (0.77%-23.19%)	16.73% (0.77%-17.40%)	22.44% (17.40%-23.19%)
**General Health Issues/Morbidity**			
Hypertension	9.12% (5.64%-11.99%)	9.79% (7.98%-11.99%)	8.89% (8.59%-10.09%)

Pre-eclampsia	1.47% (0.58%-2.35%)	2.04% (0.71%-2.35%)	1.27% (0.62%-1.58%)
Subclinical Hypothyroidism	0.69% (0.69%-0.69%)	0.69% (0.69%-0.69%)	0.69% (0.69%-0.69%)
Anxiety Disorder	5.51% (4.04%-10.34%)	5.01% (4.04%-6.24%)	5.84% (5.27%-10.34%)
Inflammatory Bowel Disease	0.01% (0.00%-0.19%)	0.01% (0.01%-0.01%)	0.00% (0.00%-0.00%)
**Uterine and Cervical Factors**			
Endometriosis	0.21% (0.13%-0.50%)	0.18% (0.17%-0.21%)	0.22% (0.22-0.23%)
Adenomyosis	0.40% (0.40%-0.40%)	0.40% (0.40%-0.40%)	0.40% (040%-0.40%)
**Fetal Characteristics**			
Twin pregnancies	3.13% (1.90%-21.70%)	4.80% (3.09%-7.63%)	2.38% (2.10%-2.84%)
**Total for final model**	**97.50%**	**95.59%**	**99.93%**
**Total for extended model**	**113.28%**	**114.98%**	**115.40%**

* Bangladesh, Bhutan, India, Nepal, Pakistan

Adjusted Population Attributable Fractions for low- and middle-income country

Afghanistan

Risk Factor	PAF	Risk Factor	PAF
Malaria	0.15%	Alcohol consumption during pregnancy	0.00%
HIV	0.00%	Short height	4.47%
Chlamydia	0.13%	Low pre-pregnancy BMI	2.58%
Trichomonas vaginalis	0.69%	Low gestational weight gain	6.84%
Heavy Physical workload during pregnancy	0.95%	Vitamin D Deficiency	11.75%
Secondhand smoking	0.45%	Hypertension	7.06%
Ambient air pollution	4.34%	Pre-eclampsia	0.48%
Maternal age < 18 & Primiparity	1.76%	Subclinical Hypothyroidism	0.46%
Maternal age 18-35 and Primiparity	3.16%	Anxiety Disorder	3.56%
Birth interval of < 18 months	2.68%	Inflammatory Bowel Disease	0.00%
Birth interval of 18-<24 months	2.16%	Endometriosis	0.15%

Birth interval of over 60 months	0.77%	Adenomyosis	0.27%
Smoking	3.85%	Twin pregnancies	2.54%
		Total	61.21%

Algeria

Risk Factor	PAF	Risk Factor	PAF
Malaria	0.00%	Alcohol consumption during pregnancy	0.11%
HIV	0.00%	Short height	3.98%
Chlamydia	0.07%	Low pre-pregnancy BMI	0.00%
Trichomonas vaginalis	0.70%	Low gestational weight gain	5.41%
Heavy Physical workload during pregnancy	0.77%	Vitamin D Deficiency	12.20%
Secondhand smoking	0.38%	Hypertension	6.00%
Ambient air pollution	3.65%	Pre-eclampsia	1.35%
Maternal age < 18 & Primiparity	2.54%	Subclinical Hypothyroidism	0.48%
Maternal age 18-35 and Primiparity	4.90%	Anxiety Disorder	3.70%
Birth interval of < 18 months	1.74%	Inflammatory Bowel Disease	0.01%
Birth interval of 18-<24 months	1.62%	Endometriosis	0.15%
Birth interval of over 60 months	2.52%	Adenomyosis	0.28%
Smoking	1.31%	Twin pregnancies	3.53%
		**Total**	57.41%

Angola

Risk Factor	PAF	Risk Factor	PAF
Malaria	1.31%	Alcohol consumption during pregnancy	0.27%
HIV	1.02%	Short height	4.28%
Chlamydia	0.13%	Low pre-pregnancy BMI	1.74%
Trichomonas vaginalis	1.16%	Low gestational weight gain	10.23%

Heavy Physical workload during pregnancy	3.37%	Vitamin D Deficiency	11.24%
Secondhand smoking	0.28%	Hypertension	6.54%
Ambient air pollution	3.37%	Pre-eclampsia	1.28%
Maternal age < 18 & Primiparity	3.70%	Subclinical Hypothyroidism	0.44%
Maternal age 18-35 and Primiparity	2.52%	Anxiety Disorder	3.41%
Birth interval of < 18 months	2.11%	Inflammatory Bowel Disease	0.01%
Birth interval of 18-<24 months	1.50%	Endometriosis	0.11%
Birth interval of over 60 months	0.98%	Adenomyosis	0.26%
Smoking	0.88%	Twin pregnancies	3.25%
		**Total**	65.40%

Azerbaijan

**Risk Factor**	**PAF**	**Risk Factor**	**PAF**
Malaria	0.00%	Alcohol consumption during pregnancy	0.57%
HIV	0.00%	Short height	0.50%
Chlamydia	0.34%	Low pre-pregnancy BMI	0.59%
Trichomonas vaginalis	1.47%	Low gestational weight gain	7.01%
Heavy Physical workload during pregnancy	3.04%	Vitamin D Deficiency	12.04%
Secondhand smoking	0.86%	Hypertension	5.57%
Ambient air pollution	3.34%	Pre-eclampsia	1.36%
Maternal age < 18 & Primiparity	0.93%	Subclinical Hypothyroidism	0.48%
Maternal age 18-35 and Primiparity	7.96%	Anxiety Disorder	3.65%
Birth interval of < 18 months	2.95%	Inflammatory Bowel Disease	0.04%
Birth interval of 18-<24 months	1.33%	Endometriosis	0.20%
Birth interval of over 60 months	2.00%	Adenomyosis	0.27%

Smoking	0.94%	Twin pregnancies	1.59%
		**Total**	59.03%

Bangladesh

Risk Factor	PAF	Risk Factor	PAF
Malaria	0.00%	Alcohol consumption during pregnancy	0.05%
HIV	0.00%	Short height	7.20%
Chlamydia	0.12%	Low pre-pregnancy BMI	3.13%
Trichomonas vaginalis	0.76%	Low gestational weight gain	6.35%
Heavy Physical workload during pregnancy	1.48%	Vitamin D Deficiency	10.90%
Secondhand smoking	0.73%	Hypertension	5.42%
Ambient air pollution	5.44%	Pre-eclampsia	0.99%
Maternal age < 18 & Primiparity	5.75%	Subclinical Hypothyroidism	0.43%
Maternal age 18-35 and Primiparity	4.53%	Anxiety Disorder	6.48%
Birth interval of < 18 months	0.73%	Inflammatory Bowel Disease	0.00%
Birth interval of 18-<24 months	0.51%	Endometriosis	0.14%
Birth interval of over 60 months	3.89%	Adenomyosis	0.25%
Smoking	0.96%	Twin pregnancies	1.78%
		**Total**	68.04%

Benin

Risk Factor	PAF	Risk Factor	PAF
Malaria	2.07%	Alcohol consumption during pregnancy	0.19%
HIV	0.52%	Short height	1.03%
Chlamydia	0.12%	Low pre-pregnancy BMI	0.00%
Trichomonas vaginalis	1.40%	Low gestational weight gain	10.74%

Heavy Physical workload during pregnancy	3.24%	Vitamin D Deficiency	11.80%
Secondhand smoking	0.18%	Hypertension	6.55%
Ambient air pollution	3.55%	Pre-eclampsia	1.34%
Maternal age < 18 & Primiparity	1.67%	Subclinical Hypothyroidism	0.47%
Maternal age 18-35 and Primiparity	3.51%	Anxiety Disorder	3.58%
Birth interval of < 18 months	1.19%	Inflammatory Bowel Disease	0.01%
Birth interval of 18-<24 months	1.11%	Endometriosis	0.12%
Birth interval of over 60 months	1.01%	Adenomyosis	0.27%
Smoking	0.58%	Twin pregnancies	5.17%
		**Total**	61.40%

Bhutan

**Risk Factor**	**PAF**	**Risk Factor**	**PAF**
Malaria	0.00%	Alcohol consumption during pregnancy	0.04%
HIV	0.06%	Short height	4.22%
Chlamydia	0.13%	Low pre-pregnancy BMI	1.74%
Trichomonas vaginalis	0.80%	Low gestational weight gain	6.73%
Heavy Physical workload during pregnancy	2.70%	Vitamin D Deficiency	11.55%
Secondhand smoking	0.18%	Hypertension	6.31%
Ambient air pollution	4.55%	Pre-eclampsia	0.83%
Maternal age < 18 & Primiparity	3.07%	Subclinical Hypothyroidism	0.46%
Maternal age 18-35 and Primiparity	5.46%	Anxiety Disorder	3.50%
Birth interval of < 18 months	1.86%	Inflammatory Bowel Disease	0.00%
Birth interval of 18-<24 months	1.19%	Endometriosis	0.15%
Birth interval of over 60 months	3.32%	Adenomyosis	0.26%

Smoking	2.11%	Twin pregnancies	1.55%
		**Total**	62.75%

Bolivia

**Risk Factor**	**PAF**	**Risk Factor**	**PAF**
Malaria	0.00%	Alcohol consumption during pregnancy	0.51%
HIV	0.04%	Short height	6.10%
Chlamydia	0.13%	Low pre-pregnancy BMI	0.38%
Trichomonas vaginalis	0.89%	Low gestational weight gain	5.29%
Heavy Physical workload during pregnancy	2.62%	Vitamin D Deficiency	11.91%
Secondhand smoking	0.68%	Hypertension	3.95%
Ambient air pollution	3.28%	Pre-eclampsia	0.75%
Maternal age < 18 & Primiparity	3.03%	Subclinical Hypothyroidism	0.47%
Maternal age 18-35 and Primiparity	4.26%	Anxiety Disorder	3.61%
Birth interval of < 18 months	1.61%	Inflammatory Bowel Disease	0.01%
Birth interval of 18-<24 months	1.43%	Endometriosis	0.09%
Birth interval of over 60 months	2.27%	Adenomyosis	0.27%
Smoking	4.83%	Twin pregnancies	1.30%
		**Total**	59.70%

Botswana

**Risk Factor**	**PAF**	**Risk Factor**	**PAF**
Malaria	0.00%	Alcohol consumption during pregnancy	0.12%
HIV	8.60%	Short height	3.90%
Chlamydia	0.25%	Low pre-pregnancy BMI	1.13%
Trichomonas vaginalis	1.84%	Low gestational weight gain	9.71%

Heavy Physical workload during pregnancy	2.80%	Vitamin D Deficiency	10.66%
Secondhand smoking	0.08%	Hypertension	6.18%
Ambient air pollution	2.13%	Pre-eclampsia	1.15%
Maternal age < 18 & Primiparity	2.84%	Subclinical Hypothyroidism	0.42%
Maternal age 18-35 and Primiparity	5.04%	Anxiety Disorder	3.23%
Birth interval of < 18 months	1.71%	Inflammatory Bowel Disease	0.01%
Birth interval of 18-<24 months	1.10%	Endometriosis	0.11%
Birth interval of over 60 months	1.43%	Adenomyosis	0.24%
Smoking	2.19%	Twin pregnancies	3.09%
		**Total**	69.97%

Burkina Faso

**Risk Factor**	**PAF**	**Risk Factor**	**PAF**
Malaria	2.26%	Alcohol consumption during pregnancy	0.27%
HIV	0.42%	Short height	0.41%
Chlamydia	0.16%	Low pre-pregnancy BMI	2.00%
Trichomonas vaginalis	2.84%	Low gestational weight gain	10.55%
Heavy Physical workload during pregnancy	2.72%	Vitamin D Deficiency	11.59%
Secondhand smoking	0.26%	Hypertension	7.47%
Ambient air pollution	3.76%	Pre-eclampsia	1.56%
Maternal age < 18 & Primiparity	2.06%	Subclinical Hypothyroidism	0.46%
Maternal age 18-35 and Primiparity	2.87%	Anxiety Disorder	3.51%
Birth interval of < 18 months	0.77%	Inflammatory Bowel Disease	0.01%
Birth interval of 18-<24 months	0.98%	Endometriosis	0.12%
Birth interval of over 60 months	0.24%	Adenomyosis	0.26%

Smoking	2.33%	Twin pregnancies	3.01%
		**Total**	62.91%

Burundi

Risk Factor	PAF	Risk Factor	PAF
Malaria	1.43%	Alcohol consumption during pregnancy	0.39%
HIV	0.58%	Short height	3.10%
Chlamydia	0.18%	Low pre-pregnancy BMI	1.87%
Trichomonas vaginalis	2.54%	Low gestational weight gain	10.43%
Heavy Physical workload during pregnancy	3.65%	Vitamin D Deficiency	11.46%
Secondhand smoking	0.20%	Hypertension	6.97%
Ambient air pollution	3.95%	Pre-eclampsia	1.35%
Maternal age < 18 & Primiparity	0.86%	Subclinical Hypothyroidism	0.45%
Maternal age 18-35 and Primiparity	3.41%	Anxiety Disorder	3.47%
Birth interval of < 18 months	1.35%	Inflammatory Bowel Disease	0.01%
Birth interval of 18-<24 months	1.24%	Endometriosis	0.12%
Birth interval of over 60 months	0.78%	Adenomyosis	0.26%
Smoking	0.51%	Twin pregnancies	3.32%
		**Total**	63.88%

Cambodia

Risk Factor	PAF	Risk Factor	PAF
Malaria	0.00%	Alcohol consumption during pregnancy	0.71%
HIV	0.26%	Short height	3.54%
Chlamydia	0.19%	Low pre-pregnancy BMI	2.18%
Trichomonas vaginalis	0.75%	Low gestational weight gain	6.81%

Heavy Physical workload during pregnancy	3.75%	Vitamin D Deficiency	11.69%
Secondhand smoking	0.71%	Hypertension	5.94%
Ambient air pollution	3.14%	Pre-eclampsia	1.31%
Maternal age < 18 & Primiparity	1.22%	Subclinical Hypothyroidism	0.46%
Maternal age 18-35 and Primiparity	6.89%	Anxiety Disorder	3.54%
Birth interval of < 18 months	0.92%	Inflammatory Bowel Disease	0.00%
Birth interval of 18-<24 months	0.65%	Endometriosis	0.12%
Birth interval of over 60 months	2.79%	Adenomyosis	0.27%
Smoking	2.13%	Twin pregnancies	1.68%
		**Total**	61.65%

Cameroon

**Risk Factor**	**PAF**	**Risk Factor**	**PAF**
Malaria	0.10%	Alcohol consumption during pregnancy	0.30%
HIV	1.76%	Short height	0.48%
Chlamydia	0.16%	Low pre-pregnancy BMI	1.12%
Trichomonas vaginalis	2.88%	Low gestational weight gain	7.09%
Heavy Physical workload during pregnancy	3.32%	Vitamin D Deficiency	11.69%
Secondhand smoking	0.17%	Hypertension	5.75%
Ambient air pollution	5.08%	Pre-eclampsia	1.34%
Maternal age < 18 & Primiparity	3.34%	Subclinical Hypothyroidism	0.46%
Maternal age 18-35 and Primiparity	3.13%	Anxiety Disorder	3.54%
Birth interval of < 18 months	1.82%	Inflammatory Bowel Disease	0.01%
Birth interval of 18-<24 months	1.31%	Endometriosis	0.12%
Birth interval of over 60 months	1.11%	Adenomyosis	0.27%

Smoking	0.92%	Twin pregnancies	4.29%
		**Total**	61.56%

Central African Republic

Risk Factor	PAF	Risk Factor	PAF
Malaria	1.97%	Alcohol consumption during pregnancy	0.22%
HIV	1.75%	Short height	1.58%
Chlamydia	0.15%	Low pre-pregnancy BMI	2.03%
Trichomonas vaginalis	1.18%	Low gestational weight gain	10.35%
Heavy Physical workload during pregnancy	2.89%	Vitamin D Deficiency	11.37%
Secondhand smoking	0.24%	Hypertension	6.85%
Ambient air pollution	3.96%	Pre-eclampsia	1.41%
Maternal age < 18 & Primiparity	3.77%	Subclinical Hypothyroidism	0.45%
Maternal age 18-35 and Primiparity	2.60%	Anxiety Disorder	3.45%
Birth interval of < 18 months	2.14%	Inflammatory Bowel Disease	0.01%
Birth interval of 18-<24 months	1.56%	Endometriosis	0.12%
Birth interval of over 60 months	0.72%	Adenomyosis	0.26%
Smoking	0.78%	Twin pregnancies	2.67%
		**Total**	64.46%

Chad

Risk Factor	PAF	Risk Factor	PAF
Malaria	0.97%	Alcohol consumption during pregnancy	0.17%
HIV	0.63%	Short height	0.41%
Chlamydia	0.16%	Low pre-pregnancy BMI	2.11%
Trichomonas vaginalis	2.10%	Low gestational weight gain	10.50%

Heavy Physical workload during pregnancy	3.00%	Vitamin D Deficiency	11.53%
Secondhand smoking	0.24%	Hypertension	7.55%
Ambient air pollution	3.99%	Pre-eclampsia	1.46%
Maternal age < 18 & Primiparity	3.26%	Subclinical Hypothyroidism	0.46%
Maternal age 18-35 and Primiparity	1.63%	Anxiety Disorder	3.50%
Birth interval of < 18 months	2.63%	Inflammatory Bowel Disease	0.01%
Birth interval of 18-<24 months	2.04%	Endometriosis	0.12%
Birth interval of over 60 months	0.54%	Adenomyosis	0.26%
Smoking	1.07%	Twin pregnancies	3.04%
		**Total**	63.35%

Comoros

**Risk Factor**	**PAF**	**Risk Factor**	**PAF**
Malaria	0.11%	Alcohol consumption during pregnancy	0.17%
HIV	0.00%	Short height	1.77%
Chlamydia	0.19%	Low pre-pregnancy BMI	1.48%
Trichomonas vaginalis	2.61%	Low gestational weight gain	10.73%
Heavy Physical workload during pregnancy	1.73%	Vitamin D Deficiency	11.79%
Secondhand smoking	0.29%	Hypertension	6.57%
Ambient air pollution	2.39%	Pre-eclampsia	1.40%
Maternal age < 18 & Primiparity	2.04%	Subclinical Hypothyroidism	0.47%
Maternal age 18-35 and Primiparity	3.57%	Anxiety Disorder	3.57%
Birth interval of < 18 months	2.82%	Inflammatory Bowel Disease	0.01%
Birth interval of 18-<24 months	1.71%	Endometriosis	0.12%
Birth interval of over 60 months	1.18%	Adenomyosis	0.27%

Smoking	0.69%	Twin pregnancies	3.61%
		**Total**	61.31%

Congo

Risk Factor	PAF	Risk Factor	PAF
Malaria	1.31%	Alcohol consumption during pregnancy	0.17%
HIV	1.69%	Short height	1.00%
Chlamydia	0.13%	Low pre-pregnancy BMI	1.81%
Trichomonas vaginalis	1.17%	Low gestational weight gain	10.45%
Heavy Physical workload during pregnancy	3.10%	Vitamin D Deficiency	11.47%
Secondhand smoking	0.23%	Hypertension	5.71%
Ambient air pollution	4.21%	Pre-eclampsia	1.20%
Maternal age < 18 & Primiparity	3.59%	Subclinical Hypothyroidism	0.45%
Maternal age 18-35 and Primiparity	3.38%	Anxiety Disorder	3.48%
Birth interval of < 18 months	1.07%	Inflammatory Bowel Disease	0.01%
Birth interval of 18-<24 months	0.91%	Endometriosis	0.12%
Birth interval of over 60 months	2.04%	Adenomyosis	0.26%
Smoking	0.68%	Twin pregnancies	4.04%
		**Total**	63.65%

Cote d'Ivoire

Risk Factor	PAF	Risk Factor	PAF
Malaria	1.99%	Alcohol consumption during pregnancy	0.25%
HIV	1.57%	Short height	0.71%
Chlamydia	0.21%	Low pre-pregnancy BMI	1.35%
Trichomonas vaginalis	1.62%	Low gestational weight gain	5.37%

Heavy Physical workload during pregnancy	2.36%	Vitamin D Deficiency	12.09%
Secondhand smoking	0.31%	Hypertension	6.43%
Ambient air pollution	2.87%	Pre-eclampsia	1.42%
Maternal age < 18 & Primiparity	3.36%	Subclinical Hypothyroidism	0.48%
Maternal age 18-35 and Primiparity	3.22%	Anxiety Disorder	3.66%
Birth interval of < 18 months	1.37%	Inflammatory Bowel Disease	0.01%
Birth interval of 18-<24 months	0.94%	Endometriosis	0.12%
Birth interval of over 60 months	1.77%	Adenomyosis	0.28%
Smoking	0.83%	Twin pregnancies	3.50%
		**Total**	58.07%

Democratic Republic of the Congo

**Risk Factor**	**PAF**	**Risk Factor**	**PAF**
Malaria	1.71%	Alcohol consumption during pregnancy	0.22%
HIV	0.44%	Short height	3.10%
Chlamydia	0.11%	Low pre-pregnancy BMI	1.97%
Trichomonas vaginalis	1.14%	Low gestational weight gain	11.16%
Heavy Physical workload during pregnancy	3.14%	Vitamin D Deficiency	11.02%
Secondhand smoking	0.28%	Hypertension	6.02%
Ambient air pollution	3.83%	Pre-eclampsia	1.49%
Maternal age < 18 & Primiparity	2.22%	Subclinical Hypothyroidism	0.44%
Maternal age 18-35 and Primiparity	2.74%	Anxiety Disorder	3.34%
Birth interval of < 18 months	2.24%	Inflammatory Bowel Disease	0.01%
Birth interval of 18-<24 months	1.64%	Endometriosis	0.11%
Birth interval of over 60 months	0.83%	Adenomyosis	0.25%

Smoking	4.56%	Twin pregnancies	3.19%
		**Total**	67.20%

Djibouti

**Risk Factor**	**PAF**	**Risk Factor**	**PAF**
Malaria	0.16%	Alcohol consumption during pregnancy	0.00%
HIV	0.42%	Short height	4.24%
Chlamydia	0.19%	Low pre-pregnancy BMI	1.76%
Trichomonas vaginalis	2.61%	Low gestational weight gain	5.15%
Heavy Physical workload during pregnancy	2.33%	Vitamin D Deficiency	11.59%
Secondhand smoking	0.45%	Hypertension	5.89%
Ambient air pollution	4.18%	Pre-eclampsia	0.46%
Maternal age < 18 & Primiparity	3.09%	Subclinical Hypothyroidism	0.46%
Maternal age 18-35 and Primiparity	5.48%	Anxiety Disorder	3.51%
Birth interval of < 18 months	1.86%	Inflammatory Bowel Disease	0.01%
Birth interval of 18-<24 months	1.20%	Endometriosis	0.12%
Birth interval of over 60 months	2.65%	Adenomyosis	0.26%
Smoking	1.57%	Twin pregnancies	2.50%
		**Total**	62.15%

Dominican Republic

**Risk Factor**	**PAF**	**Risk Factor**	**PAF**
Malaria	0.00%	Alcohol consumption during pregnancy	0.59%
HIV	0.40%	Short height	1.05%
Chlamydia	0.29%	Low pre-pregnancy BMI	0.82%
Trichomonas vaginalis	1.27%	Low gestational weight gain	5.35%

Heavy Physical workload during pregnancy	2.65%	Vitamin D Deficiency	12.04%
Secondhand smoking	0.19%	Hypertension	4.69%
Ambient air pollution	2.68%	Pre-eclampsia	0.69%
Maternal age < 18 & Primiparity	4.41%	Subclinical Hypothyroidism	0.48%
Maternal age 18-35 and Primiparity	5.61%	Anxiety Disorder	3.65%
Birth interval of < 18 months	1.62%	Inflammatory Bowel Disease	0.11%
Birth interval of 18-<24 months	0.84%	Endometriosis	0.09%
Birth interval of over 60 months	3.39%	Adenomyosis	0.27%
Smoking	2.97%	Twin pregnancies	2.39%
		**Total**	58.54%

Equatorial Guinea

**Risk Factor**	**PAF**	**Risk Factor**	**PAF**
Malaria	1.59%	Alcohol consumption during pregnancy	0.05%
HIV	3.39%	Short height	3.44%
Chlamydia	0.13%	Low pre-pregnancy BMI	1.36%
Trichomonas vaginalis	1.16%	Low gestational weight gain	10.29%
Heavy Physical workload during pregnancy	2.54%	Vitamin D Deficiency	11.30%
Secondhand smoking	0.17%	Hypertension	6.19%
Ambient air pollution	3.82%	Pre-eclampsia	1.25%
Maternal age < 18 & Primiparity	2.80%	Subclinical Hypothyroidism	0.45%
Maternal age 18-35 and Primiparity	2.62%	Anxiety Disorder	3.43%
Birth interval of < 18 months	1.51%	Inflammatory Bowel Disease	0.01%
Birth interval of 18-<24 months	1.60%	Endometriosis	0.12%
Birth interval of over 60 months	1.47%	Adenomyosis	0.26%

Smoking	0.67%	Twin pregnancies	3.27%
		**Total**	64.88%

Eritrea

Risk Factor	PAF	Risk Factor	PAF
Malaria	0.18%	Alcohol consumption during pregnancy	0.17%
HIV	0.34%	Short height	1.60%
Chlamydia	0.18%	Low pre-pregnancy BMI	2.65%
Trichomonas vaginalis	2.54%	Low gestational weight gain	10.47%
Heavy Physical workload during pregnancy	3.46%	Vitamin D Deficiency	11.51%
Secondhand smoking	0.21%	Hypertension	6.67%
Ambient air pollution	3.87%	Pre-eclampsia	1.32%
Maternal age < 18 & Primiparity	2.50%	Subclinical Hypothyroidism	0.45%
Maternal age 18-35 and Primiparity	3.23%	Anxiety Disorder	3.49%
Birth interval of < 18 months	2.11%	Inflammatory Bowel Disease	0.01%
Birth interval of 18-<24 months	1.52%	Endometriosis	0.12%
Birth interval of over 60 months	0.94%	Adenomyosis	0.26%
Smoking	0.34%	Twin pregnancies	3.33%
		**Total**	63.49%

Eswatini

Risk Factor	PAF	Risk Factor	PAF
Malaria	0.00%	Alcohol consumption during pregnancy	0.16%
HIV	11.19%	Short height	0.84%
Chlamydia	0.24%	Low pre-pregnancy BMI	0.00%
Trichomonas vaginalis	2.33%	Low gestational weight gain	10.04%

Heavy Physical workload during pregnancy	1.92%	Vitamin D Deficiency	11.03%
Secondhand smoking	0.20%	Hypertension	6.67%
Ambient air pollution	2.47%	Pre-eclampsia	1.17%
Maternal age < 18 & Primiparity	4.02%	Subclinical Hypothyroidism	0.44%
Maternal age 18-35 and Primiparity	4.17%	Anxiety Disorder	3.34%
Birth interval of < 18 months	0.91%	Inflammatory Bowel Disease	0.01%
Birth interval of 18-<24 months	0.91%	Endometriosis	0.11%
Birth interval of over 60 months	2.05%	Adenomyosis	0.25%
Smoking	0.70%	Twin pregnancies	2.70%
		**Total**	67.86%

Ethiopia

Risk Factor	PAF	Risk Factor	PAF
Malaria	0.14%	Alcohol consumption during pregnancy	0.19%
HIV	0.55%	Short height	1.60%
Chlamydia	0.18%	Low pre-pregnancy BMI	2.37%
Trichomonas vaginalis	2.69%	Low gestational weight gain	13.19%
Heavy Physical workload during pregnancy	3.54%	Vitamin D Deficiency	11.54%
Secondhand smoking	0.15%	Hypertension	7.14%
Ambient air pollution	3.50%	Pre-eclampsia	1.46%
Maternal age < 18 & Primiparity	1.84%	Subclinical Hypothyroidism	0.46%
Maternal age 18-35 and Primiparity	2.97%	Anxiety Disorder	2.68%
Birth interval of < 18 months	2.11%	Inflammatory Bowel Disease	0.01%
Birth interval of 18-<24 months	1.29%	Endometriosis	0.12%
Birth interval of over 60 months	1.35%	Adenomyosis	0.26%

Smoking	0.45%	Twin pregnancies	2.09%
		**Total**	63.86%

Gabon

**Risk Factor**	**PAF**	**Risk Factor**	**PAF**
Malaria	1.48%	Alcohol consumption during pregnancy	0.21%
HIV	2.01%	Short height	0.68%
Chlamydia	0.13%	Low pre-pregnancy BMI	1.22%
Trichomonas vaginalis	1.18%	Low gestational weight gain	10.49%
Heavy Physical workload during pregnancy	2.01%	Vitamin D Deficiency	11.52%
Secondhand smoking	0.30%	Hypertension	5.33%
Ambient air pollution	3.65%	Pre-eclampsia	1.22%
Maternal age < 18 & Primiparity	3.73%	Subclinical Hypothyroidism	0.46%
Maternal age 18-35 and Primiparity	3.93%	Anxiety Disorder	3.49%
Birth interval of < 18 months	1.42%	Inflammatory Bowel Disease	0.01%
Birth interval of 18-<24 months	1.03%	Endometriosis	0.12%
Birth interval of over 60 months	2.37%	Adenomyosis	0.26%
Smoking	1.23%	Twin pregnancies	3.73%
		**Total**	63.22%

Gambia

**Risk Factor**	**PAF**	**Risk Factor**	**PAF**
Malaria	0.36%	Alcohol consumption during pregnancy	0.22%
HIV	1.02%	Short height	0.14%
Chlamydia	0.14%	Low pre-pregnancy BMI	1.77%
Trichomonas vaginalis	2.94%	Low gestational weight gain	10.71%

Heavy Physical workload during pregnancy	2.44%	Vitamin D Deficiency	11.77%
Secondhand smoking	0.24%	Hypertension	6.68%
Ambient air pollution	4.43%	Pre-eclampsia	1.25%
Maternal age < 18 & Primiparity	1.99%	Subclinical Hypothyroidism	0.47%
Maternal age 18-35 and Primiparity	3.57%	Anxiety Disorder	3.57%
Birth interval of < 18 months	1.00%	Inflammatory Bowel Disease	0.01%
Birth interval of 18-<24 months	1.05%	Endometriosis	0.12%
Birth interval of over 60 months	1.20%	Adenomyosis	0.27%
Smoking	0.81%	Twin pregnancies	3.41%
		**Total**	61.56%

Ghana

**Risk Factor**	**PAF**	**Risk Factor**	**PAF**
Malaria	1.24%	Alcohol consumption during pregnancy	0.32%
HIV	1.01%	Short height	0.57%
Chlamydia	0.26%	Low pre-pregnancy BMI	1.20%
Trichomonas vaginalis	2.17%	Low gestational weight gain	9.77%
Heavy Physical workload during pregnancy	3.60%	Vitamin D Deficiency	12.09%
Secondhand smoking	0.13%	Hypertension	5.55%
Ambient air pollution	2.95%	Pre-eclampsia	1.21%
Maternal age < 18 & Primiparity	1.85%	Subclinical Hypothyroidism	0.48%
Maternal age 18-35 and Primiparity	4.18%	Anxiety Disorder	3.66%
Birth interval of < 18 months	0.81%	Inflammatory Bowel Disease	0.01%
Birth interval of 18-<24 months	0.97%	Endometriosis	0.12%
Birth interval of over 60 months	0.11%	Adenomyosis	0.28%

Smoking	0.54%	Twin pregnancies	3.72%
		**Total**	58.78%

Guatemala

Risk Factor	PAF	Risk Factor	PAF
Malaria	0.00%	Alcohol consumption during pregnancy	0.30%
HIV	0.13%	Short height	13.67%
Chlamydia	0.30%	Low pre-pregnancy BMI	0.52%
Trichomonas vaginalis	1.47%	Low gestational weight gain	5.11%
Heavy Physical workload during pregnancy	1.91%	Vitamin D Deficiency	11.51%
Secondhand smoking	0.27%	Hypertension	4.76%
Ambient air pollution	3.60%	Pre-eclampsia	0.68%
Maternal age < 18 & Primiparity	3.71%	Subclinical Hypothyroidism	0.45%
Maternal age 18-35 and Primiparity	4.55%	Anxiety Disorder	3.49%
Birth interval of < 18 months	1.11%	Inflammatory Bowel Disease	0.10%
Birth interval of 18-<24 months	1.14%	Endometriosis	0.09%
Birth interval of over 60 months	2.31%	Adenomyosis	0.26%
Smoking	1.40%	Twin pregnancies	1.39%
		**Total**	64.23%

Guinea

Risk Factor	PAF	Risk Factor	PAF
Malaria	1.58%	Alcohol consumption during pregnancy	0.17%
HIV	0.76%	Short height	1.22%
Chlamydia	0.16%	Low pre-pregnancy BMI	1.04%
Trichomonas vaginalis	2.10%	Low gestational weight gain	10.59%

Heavy Physical workload during pregnancy	2.94%	Vitamin D Deficiency	11.64%
Secondhand smoking	0.14%	Hypertension	7.14%
Ambient air pollution	2.71%	Pre-eclampsia	1.46%
Maternal age < 18 & Primiparity	4.31%	Subclinical Hypothyroidism	0.46%
Maternal age 18-35 and Primiparity	2.54%	Anxiety Disorder	3.53%
Birth interval of < 18 months	0.59%	Inflammatory Bowel Disease	0.01%
Birth interval of 18-<24 months	1.03%	Endometriosis	0.12%
Birth interval of over 60 months	1.31%	Adenomyosis	0.27%
Smoking	0.80%	Twin pregnancies	3.96%
		**Total**	62.56%

Guinea-Bissau

**Risk Factor**	**PAF**	**Risk Factor**	**PAF**
Malaria	0.64%	Alcohol consumption during pregnancy	0.20%
HIV	1.64%	Short height	4.38%
Chlamydia	0.16%	Low pre-pregnancy BMI	1.64%
Trichomonas vaginalis	2.02%	Low gestational weight gain	10.13%
Heavy Physical workload during pregnancy	2.92%	Vitamin D Deficiency	11.12%
Secondhand smoking	0.23%	Hypertension	6.69%
Ambient air pollution	3.17%	Pre-eclampsia	1.24%
Maternal age < 18 & Primiparity	4.39%	Subclinical Hypothyroidism	0.44%
Maternal age 18-35 and Primiparity	2.72%	Anxiety Disorder	3.37%
Birth interval of < 18 months	1.93%	Inflammatory Bowel Disease	0.01%
Birth interval of 18-<24 months	1.65%	Endometriosis	0.11%
Birth interval of over 60 months	1.46%	Adenomyosis	0.25%

Smoking	0.55%	Twin pregnancies	3.22%
		**Total**	66.28%

Guyana

**Risk Factor**	**PAF**	**Risk Factor**	**PAF**
Malaria	0.30%	Alcohol consumption during pregnancy	0.90%
HIV	0.62%	Short height	2.31%
Chlamydia	0.30%	Low pre-pregnancy BMI	0.85%
Trichomonas vaginalis	1.29%	Low gestational weight gain	5.42%
Heavy Physical workload during pregnancy	2.03%	Vitamin D Deficiency	12.20%
Secondhand smoking	0.34%	Hypertension	5.30%
Ambient air pollution	2.39%	Pre-eclampsia	0.72%
Maternal age < 18 & Primiparity	3.67%	Subclinical Hypothyroidism	0.48%
Maternal age 18-35 and Primiparity	5.03%	Anxiety Disorder	3.70%
Birth interval of < 18 months	1.85%	Inflammatory Bowel Disease	0.01%
Birth interval of 18-<24 months	1.41%	Endometriosis	0.10%
Birth interval of over 60 months	2.56%	Adenomyosis	0.28%
Smoking	1.19%	Twin pregnancies	1.89%
		**Total**	57.14%

Haiti

**Risk Factor**	**PAF**	**Risk Factor**	**PAF**
Malaria	0.01%	Alcohol consumption during pregnancy	0.73%
HIV	0.97%	Short height	0.64%
Chlamydia	0.29%	Low pre-pregnancy BMI	1.47%
Trichomonas vaginalis	1.29%	Low gestational weight gain	5.38%

Heavy Physical workload during pregnancy	3.11%	Vitamin D Deficiency	12.13%
Secondhand smoking	0.18%	Hypertension	5.77%
Ambient air pollution	3.09%	Pre-eclampsia	0.88%
Maternal age < 18 & Primiparity	2.32%	Subclinical Hypothyroidism	0.48%
Maternal age 18-35 and Primiparity	5.32%	Anxiety Disorder	3.68%
Birth interval of < 18 months	1.44%	Inflammatory Bowel Disease	0.11%
Birth interval of 18-<24 months	1.00%	Endometriosis	0.10%
Birth interval of over 60 months	2.64%	Adenomyosis	0.28%
Smoking	1.87%	Twin pregnancies	2.66%
		**Total**	57.82%

Honduras

Risk Factor	PAF	Risk Factor	PAF
Malaria	0.00%	Alcohol consumption during pregnancy	0.51%
HIV	0.13%	Short height	5.32%
Chlamydia	0.28%	Low pre-pregnancy BMI	0.68%
Trichomonas vaginalis	1.51%	Low gestational weight gain	5.28%
Heavy Physical workload during pregnancy	2.45%	Vitamin D Deficiency	11.89%
Secondhand smoking	0.35%	Hypertension	4.88%
Ambient air pollution	3.84%	Pre-eclampsia	0.69%
Maternal age < 18 & Primiparity	4.54%	Subclinical Hypothyroidism	0.47%
Maternal age 18-35 and Primiparity	5.03%	Anxiety Disorder	3.60%
Birth interval of < 18 months	1.05%	Inflammatory Bowel Disease	0.11%
Birth interval of 18-<24 months	0.84%	Endometriosis	0.09%
Birth interval of over 60 months	3.30%	Adenomyosis	0.27%

Smoking	1.05%	Twin pregnancies	1.69%
		**Total**	59.87%

India

**Risk Factor**	**PAF**	**Risk Factor**	**PAF**
Malaria	0.03%	Alcohol consumption during pregnancy	0.05%
HIV	0.06%	Short height	6.64%
Chlamydia	0.08%	Low pre-pregnancy BMI	3.27%
Trichomonas vaginalis	0.91%	Low gestational weight gain	6.61%
Heavy Physical workload during pregnancy	1.27%	Vitamin D Deficiency	15.12%
Secondhand smoking	0.35%	Hypertension	5.60%
Ambient air pollution	4.93%	Pre-eclampsia	0.91%
Maternal age < 18 & Primiparity	1.27%	Subclinical Hypothyroidism	0.45%
Maternal age 18-35 and Primiparity	6.69%	Anxiety Disorder	3.49%
Birth interval of < 18 months	1.87%	Inflammatory Bowel Disease	0.00%
Birth interval of 18-<24 months	1.25%	Endometriosis	0.15%
Birth interval of over 60 months	1.29%	Adenomyosis	0.26%
Smoking	1.54%	Twin pregnancies	1.46%
		**Total**	65.54%

Indonesia

**Risk Factor**	**PAF**	**Risk Factor**	**PAF**
Malaria	0.03%	Alcohol consumption during pregnancy	0.04%
HIV	0.07%	Short height	2.71%
Chlamydia	0.17%	Low pre-pregnancy BMI	1.88%
Trichomonas vaginalis	0.82%	Low gestational weight gain	6.98%

Heavy Physical workload during pregnancy	2.46%	Vitamin D Deficiency	11.98%
Secondhand smoking	0.99%	Hypertension	5.56%
Ambient air pollution	2.26%	Pre-eclampsia	0.82%
Maternal age < 18 & Primiparity	1.14%	Subclinical Hypothyroidism	0.47%
Maternal age 18-35 and Primiparity	7.03%	Anxiety Disorder	3.63%
Birth interval of < 18 months	0.79%	Inflammatory Bowel Disease	0.00%
Birth interval of 18-<24 months	0.50%	Endometriosis	0.13%
Birth interval of over 60 months	5.13%	Adenomyosis	0.27%
Smoking	2.19%	Twin pregnancies	1.43%
		**Total**	59.48%

Iraq

**Risk Factor**	**PAF**	**Risk Factor**	**PAF**
Malaria	0.00%	Alcohol consumption during pregnancy	0.00%
HIV	0.02%	Short height	4.68%
Chlamydia	0.20%	Low pre-pregnancy BMI	0.75%
Trichomonas vaginalis	1.13%	Low gestational weight gain	6.93%
Heavy Physical workload during pregnancy	0.92%	Vitamin D Deficiency	11.89%
Secondhand smoking	0.50%	Hypertension	5.81%
Ambient air pollution	4.46%	Pre-eclampsia	0.40%
Maternal age < 18 & Primiparity	4.70%	Subclinical Hypothyroidism	0.47%
Maternal age 18-35 and Primiparity	2.91%	Anxiety Disorder	3.60%
Birth interval of < 18 months	2.07%	Inflammatory Bowel Disease	0.04%
Birth interval of 18-<24 months	1.76%	Endometriosis	0.15%
Birth interval of over 60 months	2.04%	Adenomyosis	0.27%

Smoking	1.73%	Twin pregnancies	2.57%
		**Total**	60.00%

Jamaica

**Risk Factor**	**PAF**	**Risk Factor**	**PAF**
Malaria	0.00%	Alcohol consumption during pregnancy	0.45%
HIV	0.43%	Short height	4.32%
Chlamydia	0.29%	Low pre-pregnancy BMI	0.69%
Trichomonas vaginalis	1.25%	Low gestational weight gain	5.25%
Heavy Physical workload during pregnancy	2.73%	Vitamin D Deficiency	11.83%
Secondhand smoking	0.27%	Hypertension	4.63%
Ambient air pollution	2.42%	Pre-eclampsia	0.70%
Maternal age < 18 & Primiparity	3.15%	Subclinical Hypothyroidism	0.47%
Maternal age 18-35 and Primiparity	5.59%	Anxiety Disorder	3.59%
Birth interval of < 18 months	1.90%	Inflammatory Bowel Disease	0.11%
Birth interval of 18-<24 months	1.22%	Endometriosis	0.09%
Birth interval of over 60 months	3.39%	Adenomyosis	0.27%
Smoking	3.51%	Twin pregnancies	1.68%
		**Total**	60.23%

Kenya

**Risk Factor**	**PAF**	**Risk Factor**	**PAF**
Malaria	0.43%	Alcohol consumption during pregnancy	0.19%
HIV	2.50%	Short height	0.55%
Chlamydia	0.19%	Low pre-pregnancy BMI	1.57%
Trichomonas vaginalis	3.13%	Low gestational weight gain	10.71%

Heavy Physical workload during pregnancy	2.95%	Vitamin D Deficiency	11.76%
Secondhand smoking	0.31%	Hypertension	6.24%
Ambient air pollution	2.33%	Pre-eclampsia	1.38%
Maternal age < 18 & Primiparity	2.41%	Subclinical Hypothyroidism	0.47%
Maternal age 18-35 and Primiparity	4.25%	Anxiety Disorder	3.57%
Birth interval of < 18 months	1.38%	Inflammatory Bowel Disease	0.01%
Birth interval of 18-<24 months	1.10%	Endometriosis	0.12%
Birth interval of over 60 months	0.04%	Adenomyosis	0.27%
Smoking	0.58%	Twin pregnancies	3.10%
		**Total**	61.52%

Kyrgyzstan

**Risk Factor**	**PAF**	**Risk Factor**	**PAF**
Malaria	0.00%	Alcohol consumption during pregnancy	1.15%
HIV	0.04%	Short height	0.43%
Chlamydia	0.34%	Low pre-pregnancy BMI	0.84%
Trichomonas vaginalis	1.46%	Low gestational weight gain	7.11%
Heavy Physical workload during pregnancy	2.39%	Vitamin D Deficiency	12.21%
Secondhand smoking	0.71%	Hypertension	6.25%
Ambient air pollution	2.43%	Pre-eclampsia	1.11%
Maternal age < 18 & Primiparity	0.36%	Subclinical Hypothyroidism	0.48%
Maternal age 18-35 and Primiparity	6.62%	Anxiety Disorder	3.70%
Birth interval of < 18 months	1.87%	Inflammatory Bowel Disease	0.04%
Birth interval of 18-<24 months	1.47%	Endometriosis	0.20%
Birth interval of over 60 months	2.57%	Adenomyosis	0.28%

Smoking	1.66%	Twin pregnancies	1.81%
		**Total**	57.54%

Laos

Risk Factor	PAF	Risk Factor	PAF
Malaria	0.01%	Alcohol consumption during pregnancy	0.89%
HIV	0.08%	Short height	3.72%
Chlamydia	0.19%	Low pre-pregnancy BMI	1.93%
Trichomonas vaginalis	0.75%	Low gestational weight gain	6.64%
Heavy Physical workload during pregnancy	3.48%	Vitamin D Deficiency	11.40%
Secondhand smoking	0.94%	Hypertension	5.67%
Ambient air pollution	3.31%	Pre-eclampsia	1.35%
Maternal age < 18 & Primiparity	2.37%	Subclinical Hypothyroidism	0.45%
Maternal age 18-35 and Primiparity	4.58%	Anxiety Disorder	3.45%
Birth interval of < 18 months	1.63%	Inflammatory Bowel Disease	0.00%
Birth interval of 18-<24 months	1.52%	Endometriosis	0.12%
Birth interval of over 60 months	2.64%	Adenomyosis	0.26%
Smoking	5.06%	Twin pregnancies	1.30%
		**Total**	63.74%

Lesotho

Risk Factor	PAF	Risk Factor	PAF
Malaria	0.00%	Alcohol consumption during pregnancy	0.21%
HIV	9.11%	Short height	0.89%
Chlamydia	0.24%	Low pre-pregnancy BMI	1.04%
Trichomonas vaginalis	2.31%	Low gestational weight gain	9.95%

Heavy Physical workload during pregnancy	2.61%	Vitamin D Deficiency	10.92%
Secondhand smoking	0.73%	Hypertension	6.59%
Ambient air pollution	2.56%	Pre-eclampsia	1.16%
Maternal age < 18 & Primiparity	2.93%	Subclinical Hypothyroidism	0.43%
Maternal age 18-35 and Primiparity	5.79%	Anxiety Disorder	3.31%
Birth interval of < 18 months	0.62%	Inflammatory Bowel Disease	0.01%
Birth interval of 18-<24 months	0.51%	Endometriosis	0.11%
Birth interval of over 60 months	2.90%	Adenomyosis	0.25%
Smoking	0.48%	Twin pregnancies	2.62%
		**Total**	68.28%

Liberia

Risk Factor	PAF	Risk Factor	PAF
Malaria	2.07%	Alcohol consumption during pregnancy	0.24%
HIV	0.77%	Short height	1.50%
Chlamydia	0.16%	Low pre-pregnancy BMI	1.46%
Trichomonas vaginalis	2.11%	Low gestational weight gain	10.75%
Heavy Physical workload during pregnancy	2.57%	Vitamin D Deficiency	11.81%
Secondhand smoking	0.22%	Hypertension	6.60%
Ambient air pollution	1.22%	Pre-eclampsia	1.25%
Maternal age < 18 & Primiparity	3.97%	Subclinical Hypothyroidism	0.47%
Maternal age 18-35 and Primiparity	3.27%	Anxiety Disorder	3.58%
Birth interval of < 18 months	1.07%	Inflammatory Bowel Disease	0.01%
Birth interval of 18-<24 months	1.05%	Endometriosis	0.12%
Birth interval of over 60 months	0.20%	Adenomyosis	0.27%

Smoking	0.52%	Twin pregnancies	3.93%
		**Total**	61.20%

Madagascar

Risk Factor	PAF	Risk Factor	PAF
Malaria	0.47%	Alcohol consumption during pregnancy	0.18%
HIV	0.09%	Short height	4.59%
Chlamydia	0.18%	Low pre-pregnancy BMI	2.29%
Trichomonas vaginalis	2.55%	Low gestational weight gain	10.53%
Heavy Physical workload during pregnancy	3.83%	Vitamin D Deficiency	11.57%
Secondhand smoking	0.39%	Hypertension	6.44%
Ambient air pollution	2.52%	Pre-eclampsia	1.38%
Maternal age < 18 & Primiparity	2.76%	Subclinical Hypothyroidism	0.46%
Maternal age 18-35 and Primiparity	3.44%	Anxiety Disorder	3.51%
Birth interval of < 18 months	1.33%	Inflammatory Bowel Disease	0.01%
Birth interval of 18-<24 months	1.19%	Endometriosis	0.12%
Birth interval of over 60 months	0.06%	Adenomyosis	0.26%
Smoking	0.85%	Twin pregnancies	2.05%
		**Total**	63.06%

Malawi

Risk Factor	PAF	Risk Factor	PAF
Malaria	1.11%	Alcohol consumption during pregnancy	0.18%
HIV	4.22%	Short height	1.80%
Chlamydia	0.15%	Low pre-pregnancy BMI	1.51%
Trichomonas vaginalis	1.50%	Low gestational weight gain	13.91%

Heavy Physical workload during pregnancy	3.22%	Vitamin D Deficiency	11.17%
Secondhand smoking	0.30%	Hypertension	6.50%
Ambient air pollution	2.80%	Pre-eclampsia	1.14%
Maternal age < 18 & Primiparity	3.28%	Subclinical Hypothyroidism	0.44%
Maternal age 18-35 and Primiparity	3.57%	Anxiety Disorder	3.39%
Birth interval of < 18 months	0.88%	Inflammatory Bowel Disease	0.01%
Birth interval of 18-<24 months	0.66%	Endometriosis	0.11%
Birth interval of over 60 months	0.02%	Adenomyosis	0.25%
Smoking	0.77%	Twin pregnancies	3.88%
		**Total**	66.79%

Mali

**Risk Factor**	**PAF**	**Risk Factor**	**PAF**
Malaria	2.00%	Alcohol consumption during pregnancy	0.17%
HIV	0.67%	Short height	0.27%
Chlamydia	0.18%	Low pre-pregnancy BMI	1.76%
Trichomonas vaginalis	2.26%	Low gestational weight gain	10.54%
Heavy Physical workload during pregnancy	2.83%	Vitamin D Deficiency	11.57%
Secondhand smoking	0.22%	Hypertension	7.54%
Ambient air pollution	3.93%	Pre-eclampsia	1.41%
Maternal age < 18 & Primiparity	3.18%	Subclinical Hypothyroidism	0.46%
Maternal age 18-35 and Primiparity	2.22%	Anxiety Disorder	3.51%
Birth interval of < 18 months	1.92%	Inflammatory Bowel Disease	0.01%
Birth interval of 18-<24 months	1.36%	Endometriosis	0.12%
Birth interval of over 60 months	0.97%	Adenomyosis	0.26%

Smoking	0.40%	Twin pregnancies	3.26%
		**Total**	63.04%

Mauritania

**Risk Factor**	**PAF**	**Risk Factor**	**PAF**
Malaria	0.24%	Alcohol consumption during pregnancy	0.16%
HIV	0.09%	Short height	0.68%
Chlamydia	0.09%	Low pre-pregnancy BMI	1.54%
Trichomonas vaginalis	2.10%	Low gestational weight gain	10.60%
Heavy Physical workload during pregnancy	1.48%	Vitamin D Deficiency	11.64%
Secondhand smoking	0.31%	Hypertension	7.14%
Ambient air pollution	5.41%	Pre-eclampsia	1.31%
Maternal age < 18 & Primiparity	2.53%	Subclinical Hypothyroidism	0.46%
Maternal age 18-35 and Primiparity	3.27%	Anxiety Disorder	3.53%
Birth interval of < 18 months	2.13%	Inflammatory Bowel Disease	0.01%
Birth interval of 18-<24 months	1.54%	Endometriosis	0.12%
Birth interval of over 60 months	1.41%	Adenomyosis	0.27%
Smoking	1.36%	Twin pregnancies	3.17%
		**Total**	62.59%

Morocco

**Risk Factor**	**PAF**	**Risk Factor**	**PAF**
Malaria	0.00%	Alcohol consumption during pregnancy	0.00%
HIV	0.00%	Short height	0.75%
Chlamydia	0.15%	Low pre-pregnancy BMI	0.80%
Trichomonas vaginalis	0.67%	Low gestational weight gain	5.68%

Heavy Physical workload during pregnancy	1.32%	Vitamin D Deficiency	12.79%
Secondhand smoking	0.36%	Hypertension	6.57%
Ambient air pollution	3.13%	Pre-eclampsia	0.50%
Maternal age < 18 & Primiparity	1.26%	Subclinical Hypothyroidism	0.51%
Maternal age 18-35 and Primiparity	5.21%	Anxiety Disorder	3.88%
Birth interval of < 18 months	1.63%	Inflammatory Bowel Disease	0.01%
Birth interval of 18-<24 months	1.23%	Endometriosis	0.16%
Birth interval of over 60 months	2.87%	Adenomyosis	0.29%
Smoking	0.57%	Twin pregnancies	2.35%
		**Total**	52.70%

Mozambique

**Risk Factor**	**PAF**	**Risk Factor**	**PAF**
Malaria	0.00%	Alcohol consumption during pregnancy	0.19%
HIV	5.39%	Short height	2.65%
Chlamydia	0.22%	Low pre-pregnancy BMI	1.64%
Trichomonas vaginalis	3.09%	Low gestational weight gain	10.11%
Heavy Physical workload during pregnancy	3.63%	Vitamin D Deficiency	11.11%
Secondhand smoking	0.34%	Hypertension	6.48%
Ambient air pollution	2.36%	Pre-eclampsia	1.30%
Maternal age < 18 & Primiparity	3.71%	Subclinical Hypothyroidism	0.44%
Maternal age 18-35 and Primiparity	2.54%	Anxiety Disorder	3.37%
Birth interval of < 18 months	1.09%	Inflammatory Bowel Disease	0.01%
Birth interval of 18-<24 months	1.32%	Endometriosis	0.11%
Birth interval of over 60 months	0.05%	Adenomyosis	0.25%

Smoking	1.66%	Twin pregnancies	3.45%
		**Total**	66.49%

Myanmar

Risk Factor	PAF	Risk Factor	PAF
Malaria	0.01%	Alcohol consumption during pregnancy	0.05%
HIV	0.17%	Short height	4.15%
Chlamydia	0.19%	Low pre-pregnancy BMI	2.04%
Trichomonas vaginalis	0.75%	Low gestational weight gain	6.80%
Heavy Physical workload during pregnancy	2.42%	Vitamin D Deficiency	11.68%
Secondhand smoking	0.54%	Hypertension	5.66%
Ambient air pollution	4.63%	Pre-eclampsia	0.90%
Maternal age < 18 & Primiparity	1.00%	Subclinical Hypothyroidism	0.46%
Maternal age 18-35 and Primiparity	6.16%	Anxiety Disorder	3.54%
Birth interval of < 18 months	0.90%	Inflammatory Bowel Disease	0.00%
Birth interval of 18-<24 months	0.72%	Endometriosis	0.12%
Birth interval of over 60 months	3.51%	Adenomyosis	0.27%
Smoking	3.56%	Twin pregnancies	1.56%
		**Total**	61.80%

Namibia

Risk Factor	PAF	Risk Factor	PAF
Malaria	0.13%	Alcohol consumption during pregnancy	0.32%
HIV	5.71%	Short height	0.33%
Chlamydia	0.25%	Low pre-pregnancy BMI	1.43%
Trichomonas vaginalis	2.35%	Low gestational weight gain	10.07%

Heavy Physical workload during pregnancy	2.61%	Vitamin D Deficiency	11.07%
Secondhand smoking	0.36%	Hypertension	6.20%
Ambient air pollution	2.48%	Pre-eclampsia	1.22%
Maternal age < 18 & Primiparity	2.45%	Subclinical Hypothyroidism	0.44%
Maternal age 18-35 and Primiparity	5.25%	Anxiety Disorder	3.35%
Birth interval of < 18 months	0.84%	Inflammatory Bowel Disease	0.01%
Birth interval of 18-<24 months	0.73%	Endometriosis	0.11%
Birth interval of over 60 months	2.97%	Adenomyosis	0.25%
Smoking	3.53%	Twin pregnancies	2.37%
		**Total**	66.82%

Nepal

**Risk Factor**	**PAF**	**Risk Factor**	**PAF**
Malaria	0.00%	Alcohol consumption during pregnancy	0.05%
HIV	0.00%	Short height	6.19%
Chlamydia	0.12%	Low pre-pregnancy BMI	2.34%
Trichomonas vaginalis	0.76%	Low gestational weight gain	6.37%
Heavy Physical workload during pregnancy	3.58%	Vitamin D Deficiency	10.93%
Secondhand smoking	0.53%	Hypertension	6.34%
Ambient air pollution	4.96%	Pre-eclampsia	0.75%
Maternal age < 18 & Primiparity	3.08%	Subclinical Hypothyroidism	0.43%
Maternal age 18-35 and Primiparity	5.87%	Anxiety Disorder	3.31%
Birth interval of < 18 months	1.43%	Inflammatory Bowel Disease	0.00%
Birth interval of 18-<24 months	0.92%	Endometriosis	0.14%
Birth interval of over 60 months	1.82%	Adenomyosis	0.25%

Smoking	6.21%	Twin pregnancies	1.32%
		**Total**	67.70%

Nicaragua

Risk Factor	PAF	Risk Factor	PAF
Malaria	0.02%	Alcohol consumption during pregnancy	0.43%
HIV	0.04%	Short height	3.50%
Chlamydia	0.18%	Low pre-pregnancy BMI	0.62%
Trichomonas vaginalis	1.52%	Low gestational weight gain	5.31%
Heavy Physical workload during pregnancy	2.44%	Vitamin D Deficiency	11.96%
Secondhand smoking	0.27%	Hypertension	4.84%
Ambient air pollution	3.12%	Pre-eclampsia	0.70%
Maternal age < 18 & Primiparity	4.98%	Subclinical Hypothyroidism	0.47%
Maternal age 18-35 and Primiparity	4.12%	Anxiety Disorder	3.63%
Birth interval of < 18 months	2.32%	Inflammatory Bowel Disease	0.11%
Birth interval of 18-<24 months	1.35%	Endometriosis	0.09%
Birth interval of over 60 months	2.10%	Adenomyosis	0.27%
Smoking	3.06%	Twin pregnancies	1.68%
		**Total**	59.14%

Niger

Risk Factor	PAF	Risk Factor	PAF
Malaria	2.07%	Alcohol consumption during pregnancy	0.17%
HIV	0.13%	Short height	0.54%
Chlamydia	0.14%	Low pre-pregnancy BMI	2.01%
Trichomonas vaginalis	2.07%	Low gestational weight gain	12.27%

Heavy Physical workload during pregnancy	3.09%	Vitamin D Deficiency	11.45%
Secondhand smoking	0.16%	Hypertension	7.89%
Ambient air pollution	4.70%	Pre-eclampsia	1.44%
Maternal age < 18 & Primiparity	2.62%	Subclinical Hypothyroidism	0.45%
Maternal age 18-35 and Primiparity	1.74%	Anxiety Disorder	3.47%
Birth interval of < 18 months	1.71%	Inflammatory Bowel Disease	0.01%
Birth interval of 18-<24 months	1.72%	Endometriosis	0.12%
Birth interval of over 60 months	0.54%	Adenomyosis	0.26%
Smoking	0.40%	Twin pregnancies	3.31%
		**Total**	64.48%

Nigeria

**Risk Factor**	**PAF**	**Risk Factor**	**PAF**
Malaria	1.65%	Alcohol consumption during pregnancy	0.19%
HIV	0.68%	Short height	0.96%
Chlamydia	0.32%	Low pre-pregnancy BMI	1.52%
Trichomonas vaginalis	1.91%	Low gestational weight gain	11.67%
Heavy Physical workload during pregnancy	2.39%	Vitamin D Deficiency	11.71%
Secondhand smoking	0.12%	Hypertension	5.85%
Ambient air pollution	3.82%	Pre-eclampsia	1.35%
Maternal age < 18 & Primiparity	2.51%	Subclinical Hypothyroidism	0.46%
Maternal age 18-35 and Primiparity	2.89%	Anxiety Disorder	3.14%
Birth interval of < 18 months	1.60%	Inflammatory Bowel Disease	0.01%
Birth interval of 18-<24 months	1.70%	Endometriosis	0.12%
Birth interval of over 60 months	0.87%	Adenomyosis	0.27%

Smoking	0.75%	Twin pregnancies	3.61%
		**Total**	62.04%

North Korea

Risk Factor	PAF	Risk Factor	PAF
Malaria	0.00%	Alcohol consumption during pregnancy	0.04%
HIV	0.06%	Short height	4.28%
Chlamydia	0.20%	Low pre-pregnancy BMI	1.53%
Trichomonas vaginalis	0.87%	Low gestational weight gain	6.82%
Heavy Physical workload during pregnancy	3.47%	Vitamin D Deficiency	11.72%
Secondhand smoking	0.76%	Hypertension	5.68%
Ambient air pollution	3.44%	Pre-eclampsia	0.60%
Maternal age < 18 & Primiparity	3.12%	Subclinical Hypothyroidism	0.46%
Maternal age 18-35 and Primiparity	5.54%	Anxiety Disorder	3.55%
Birth interval of < 18 months	1.88%	Inflammatory Bowel Disease	0.01%
Birth interval of 18-<24 months	1.21%	Endometriosis	0.34%
Birth interval of over 60 months	3.43%	Adenomyosis	0.27%
Smoking	0.52%	Twin pregnancies	1.57%
		**Total**	61.39%

Pakistan

Risk Factor	PAF	Risk Factor	PAF
Malaria	0.02%	Alcohol consumption during pregnancy	0.00%
HIV	0.00%	Short height	3.29%
Chlamydia	0.20%	Low pre-pregnancy BMI	2.33%
Trichomonas vaginalis	0.70%	Low gestational weight gain	6.80%

Heavy Physical workload during pregnancy	1.20%	Vitamin D Deficiency	14.71%
Secondhand smoking	0.35%	Hypertension	6.77%
Ambient air pollution	5.02%	Pre-eclampsia	0.42%
Maternal age < 18 & Primiparity	1.01%	Subclinical Hypothyroidism	0.46%
Maternal age 18-35 and Primiparity	4.19%	Anxiety Disorder	4.03%
Birth interval of < 18 months	3.74%	Inflammatory Bowel Disease	0.00%
Birth interval of 18-<24 months	1.88%	Endometriosis	0.15%
Birth interval of over 60 months	0.90%	Adenomyosis	0.27%
Smoking	2.40%	Twin pregnancies	1.85%
		**Total**	62.68%

Panama

**Risk Factor**	**PAF**	**Risk Factor**	**PAF**
Malaria	0.00%	Alcohol consumption during pregnancy	1.24%
HIV	0.00%	Short height	4.04%
Chlamydia	0.32%	Low pre-pregnancy BMI	0.49%
Trichomonas vaginalis	2.32%	Low gestational weight gain	4.90%
Heavy Physical workload during pregnancy	2.36%	Vitamin D Deficiency	11.05%
Secondhand smoking	0.26%	Hypertension	3.92%
Ambient air pollution	1.89%	Pre-eclampsia	0.50%
Maternal age < 18 & Primiparity	2.94%	Subclinical Hypothyroidism	0.44%
Maternal age 18-35 and Primiparity	5.22%	Anxiety Disorder	3.35%
Birth interval of < 18 months	1.78%	Inflammatory Bowel Disease	0.10%
Birth interval of 18-<24 months	1.14%	Endometriosis	0.09%
Birth interval of over 60 months	2.72%	Adenomyosis	0.25%

Smoking	2.53%	Twin pregnancies	13.78%
		**Total**	67.61%

Papua New Guinea

Risk Factor	PAF	Risk Factor	PAF
Malaria	1.03%	Alcohol consumption during pregnancy	0.65%
HIV	0.41%	Short height	6.92%
Chlamydia	0.40%	Low pre-pregnancy BMI	0.67%
Trichomonas vaginalis	2.29%	Low gestational weight gain	4.98%
Heavy Physical workload during pregnancy	3.09%	Vitamin D Deficiency	11.22%
Secondhand smoking	0.75%	Hypertension	5.76%
Ambient air pollution	1.73%	Pre-eclampsia	1.40%
Maternal age < 18 & Primiparity	0.54%	Subclinical Hypothyroidism	0.44%
Maternal age 18-35 and Primiparity	3.96%	Anxiety Disorder	3.40%
Birth interval of < 18 months	3.13%	Inflammatory Bowel Disease	0.08%
Birth interval of 18-<24 months	1.99%	Endometriosis	0.18%
Birth interval of over 60 months	1.40%	Adenomyosis	0.26%
Smoking	7.39%	Twin pregnancies	1.28%
		**Total**	65.35%

Paraguay

Risk Factor	PAF	Risk Factor	PAF
Malaria	0.00%	Alcohol consumption during pregnancy	0.86%
HIV	0.13%	Short height	4.35%
Chlamydia	0.31%	Low pre-pregnancy BMI	0.57%
Trichomonas vaginalis	1.49%	Low gestational weight gain	5.28%

Heavy Physical workload during pregnancy	2.72%	Vitamin D Deficiency	11.90%
Secondhand smoking	0.27%	Hypertension	5.13%
Ambient air pollution	2.21%	Pre-eclampsia	0.68%
Maternal age < 18 & Primiparity	2.05%	Subclinical Hypothyroidism	0.47%
Maternal age 18-35 and Primiparity	4.07%	Anxiety Disorder	3.61%
Birth interval of < 18 months	3.58%	Inflammatory Bowel Disease	0.01%
Birth interval of 18-<24 months	2.07%	Endometriosis	0.10%
Birth interval of over 60 months	1.29%	Adenomyosis	0.27%
Smoking	4.26%	Twin pregnancies	1.92%
		**Total**	59.60%

Philippines

**Risk Factor**	**PAF**	**Risk Factor**	**PAF**
Malaria	0.00%	Alcohol consumption during pregnancy	1.19%
HIV	0.00%	Short height	0.58%
Chlamydia	0.19%	Low pre-pregnancy BMI	0.61%
Trichomonas vaginalis	1.13%	Low gestational weight gain	5.49%
Heavy Physical workload during pregnancy	2.37%	Vitamin D Deficiency	12.37%
Secondhand smoking	0.71%	Hypertension	6.18%
Ambient air pollution	2.60%	Pre-eclampsia	1.23%
Maternal age < 18 & Primiparity	0.70%	Subclinical Hypothyroidism	0.49%
Maternal age 18-35 and Primiparity	6.53%	Anxiety Disorder	3.75%
Birth interval of < 18 months	1.24%	Inflammatory Bowel Disease	0.04%
Birth interval of 18-<24 months	0.80%	Endometriosis	0.17%
Birth interval of over 60 months	3.72%	Adenomyosis	0.28%

Smoking	1.38%	Twin pregnancies	2.38%
		**Total**	56.16%

Rwanda

**Risk Factor**	**PAF**	**Risk Factor**	**PAF**
Malaria	2.55%	Alcohol consumption during pregnancy	0.42%
HIV	1.39%	Short height	1.89%
Chlamydia	0.16%	Low pre-pregnancy BMI	1.29%
Trichomonas vaginalis	2.53%	Low gestational weight gain	10.35%
Heavy Physical workload during pregnancy	3.86%	Vitamin D Deficiency	11.37%
Secondhand smoking	0.25%	Hypertension	6.27%
Ambient air pollution	4.14%	Pre-eclampsia	1.52%
Maternal age < 18 & Primiparity	0.84%	Subclinical Hypothyroidism	0.45%
Maternal age 18-35 and Primiparity	5.29%	Anxiety Disorder	3.44%
Birth interval of < 18 months	1.01%	Inflammatory Bowel Disease	0.01%
Birth interval of 18-<24 months	0.83%	Endometriosis	0.11%
Birth interval of over 60 months	0.13%	Adenomyosis	0.26%
Smoking	2.07%	Twin pregnancies	2.09%
		**Total**	64.55%

Senegal

**Risk Factor**	**PAF**	**Risk Factor**	**PAF**
Malaria	0.32%	Alcohol consumption during pregnancy	0.17%
HIV	0.22%	Short height	0.14%
Chlamydia	0.13%	Low pre-pregnancy BMI	1.89%
Trichomonas vaginalis	2.13%	Low gestational weight gain	10.75%

Heavy Physical workload during pregnancy	2.19%	Vitamin D Deficiency	11.81%
Secondhand smoking	0.18%	Hypertension	7.04%
Ambient air pollution	3.82%	Pre-eclampsia	1.25%
Maternal age < 18 & Primiparity	2.06%	Subclinical Hypothyroidism	0.47%
Maternal age 18-35 and Primiparity	5.59%	Anxiety Disorder	3.58%
Birth interval of < 18 months	1.06%	Inflammatory Bowel Disease	0.01%
Birth interval of 18-<24 months	1.11%	Endometriosis	0.12%
Birth interval of over 60 months	1.28%	Adenomyosis	0.27%
Smoking	0.87%	Twin pregnancies	2.91%
		**Total**	61.36%

Sierra Leone

**Risk Factor**	**PAF**	**Risk Factor**	**PAF**
Malaria	1.69%	Alcohol consumption during pregnancy	0.34%
HIV	0.74%	Short height	1.38%
Chlamydia	0.16%	Low pre-pregnancy BMI	1.58%
Trichomonas vaginalis	2.01%	Low gestational weight gain	10.29%
Heavy Physical workload during pregnancy	2.60%	Vitamin D Deficiency	11.30%
Secondhand smoking	0.44%	Hypertension	6.86%
Ambient air pollution	2.28%	Pre-eclampsia	0.86%
Maternal age < 18 & Primiparity	3.25%	Subclinical Hypothyroidism	0.45%
Maternal age 18-35 and Primiparity	5.34%	Anxiety Disorder	3.43%
Birth interval of < 18 months	1.00%	Inflammatory Bowel Disease	0.01%
Birth interval of 18-<24 months	1.15%	Endometriosis	0.11%
Birth interval of over 60 months	2.10%	Adenomyosis	0.26%

Smoking	2.06%	Twin pregnancies	3.29%
		**Total**	64.98%

Solomon Islands

**Risk Factor**	**PAF**	**Risk Factor**	**PAF**
Malaria	0.79%	Alcohol consumption during pregnancy	0.64%
HIV	0.08%	Short height	7.13%
Chlamydia	0.26%	Low pre-pregnancy BMI	0.55%
Trichomonas vaginalis	1.62%	Low gestational weight gain	5.13%
Heavy Physical workload during pregnancy	2.90%	Vitamin D Deficiency	11.56%
Secondhand smoking	0.58%	Hypertension	5.47%
Ambient air pollution	0.85%	Pre-eclampsia	1.35%
Maternal age < 18 & Primiparity	0.56%	Subclinical Hypothyroidism	0.46%
Maternal age 18-35 and Primiparity	5.46%	Anxiety Disorder	3.50%
Birth interval of < 18 months	3.22%	Inflammatory Bowel Disease	0.08%
Birth interval of 18-<24 months	2.05%	Endometriosis	0.19%
Birth interval of over 60 months	1.52%	Adenomyosis	0.26%
Smoking	5.13%	Twin pregnancies	1.32%
		**Total**	62.69%

Somalia

**Risk Factor**	**PAF**	**Risk Factor**	**PAF**
Malaria	0.23%	Alcohol consumption during pregnancy	0.00%
HIV	0.04%	Short height	1.85%
Chlamydia	0.16%	Low pre-pregnancy BMI	2.08%
Trichomonas vaginalis	2.68%	Low gestational weight gain	10.89%

Heavy Physical workload during pregnancy	0.92%	Vitamin D Deficiency	11.96%
Secondhand smoking	0.28%	Hypertension	7.51%
Ambient air pollution	2.48%	Pre-eclampsia	0.51%
Maternal age < 18 & Primiparity	2.13%	Subclinical Hypothyroidism	0.47%
Maternal age 18-35 and Primiparity	5.66%	Anxiety Disorder	3.63%
Birth interval of < 18 months	0.80%	Inflammatory Bowel Disease	0.01%
Birth interval of 18-<24 months	1.21%	Endometriosis	0.12%
Birth interval of over 60 months	0.84%	Adenomyosis	0.27%
Smoking	0.94%	Twin pregnancies	2.58%
		**Total**	60.26%

South Africa

**Risk Factor**	**PAF**	**Risk Factor**	**PAF**
Malaria	0.00%	Alcohol consumption during pregnancy	0.30%
HIV	7.81%	Short height	0.97%
Chlamydia	0.36%	Low pre-pregnancy BMI	0.68%
Trichomonas vaginalis	3.34%	Low gestational weight gain	4.31%
Heavy Physical workload during pregnancy	2.16%	Vitamin D Deficiency	11.14%
Secondhand smoking	0.43%	Hypertension	5.79%
Ambient air pollution	3.16%	Pre-eclampsia	1.18%
Maternal age < 18 & Primiparity	2.82%	Subclinical Hypothyroidism	0.44%
Maternal age 18-35 and Primiparity	5.27%	Anxiety Disorder	3.38%
Birth interval of < 18 months	1.02%	Inflammatory Bowel Disease	0.01%
Birth interval of 18-<24 months	0.84%	Endometriosis	0.12%
Birth interval of over 60 months	3.89%	Adenomyosis	0.25%

Smoking	3.89%	Twin pregnancies	2.30%
		**Total**	65.86%

South Sudan

Risk Factor	PAF	Risk Factor	PAF
Malaria	1.46%	Alcohol consumption during pregnancy	0.17%
HIV	1.18%	Short height	4.43%
Chlamydia	0.19%	Low pre-pregnancy BMI	0.00%
Trichomonas vaginalis	2.59%	Low gestational weight gain	5.17%
Heavy Physical workload during pregnancy	3.29%	Vitamin D Deficiency	11.64%
Secondhand smoking	0.28%	Hypertension	6.38%
Ambient air pollution	3.39%	Pre-eclampsia	0.47%
Maternal age < 18 & Primiparity	2.07%	Subclinical Hypothyroidism	0.46%
Maternal age 18-35 and Primiparity	5.50%	Anxiety Disorder	3.53%
Birth interval of < 18 months	1.95%	Inflammatory Bowel Disease	0.01%
Birth interval of 18-<24 months	1.57%	Endometriosis	0.12%
Birth interval of over 60 months	1.42%	Adenomyosis	0.27%
Smoking	0.97%	Twin pregnancies	3.37%
		**Total**	61.85%

Sudan

Risk Factor	PAF	Risk Factor	PAF
Malaria	0.30%	Alcohol consumption during pregnancy	0.00%
HIV	0.09%	Short height	4.81%
Chlamydia	0.17%	Low pre-pregnancy BMI	1.46%
Trichomonas vaginalis	1.05%	Low gestational weight gain	10.59%

Heavy Physical workload during pregnancy	1.14%	Vitamin D Deficiency	11.64%
Secondhand smoking	0.03%	Hypertension	6.77%
Ambient air pollution	4.26%	Pre-eclampsia	0.42%
Maternal age < 18 & Primiparity	1.87%	Subclinical Hypothyroidism	0.46%
Maternal age 18-35 and Primiparity	5.50%	Anxiety Disorder	3.53%
Birth interval of < 18 months	2.89%	Inflammatory Bowel Disease	0.01%
Birth interval of 18-<24 months	1.69%	Endometriosis	0.14%
Birth interval of over 60 months	0.66%	Adenomyosis	0.27%
Smoking	0.23%	Twin pregnancies	2.83%
		**Total**	62.78%

Suriname

**Risk Factor**	**PAF**	**Risk Factor**	**PAF**
Malaria	0.00%	Alcohol consumption during pregnancy	0.49%
HIV	0.52%	Short height	4.32%
Chlamydia	0.29%	Low pre-pregnancy BMI	0.72%
Trichomonas vaginalis	1.26%	Low gestational weight gain	5.24%
Heavy Physical workload during pregnancy	2.01%	Vitamin D Deficiency	11.82%
Secondhand smoking	0.57%	Hypertension	4.78%
Ambient air pollution	2.37%	Pre-eclampsia	0.76%
Maternal age < 18 & Primiparity	3.15%	Subclinical Hypothyroidism	0.47%
Maternal age 18-35 and Primiparity	5.59%	Anxiety Disorder	3.58%
Birth interval of < 18 months	1.90%	Inflammatory Bowel Disease	0.01%
Birth interval of 18-<24 months	1.22%	Endometriosis	0.09%
Birth interval of over 60 months	2.95%	Adenomyosis	0.27%

Smoking	4.13%	Twin pregnancies	1.83%
		**Total**	60.33%

Tajikistan

**Risk Factor**	**PAF**	**Risk Factor**	**PAF**
Malaria	0.00%	Alcohol consumption during pregnancy	0.94%
HIV	0.04%	Short height	0.79%
Chlamydia	0.34%	Low pre-pregnancy BMI	1.02%
Trichomonas vaginalis	1.48%	Low gestational weight gain	7.11%
Heavy Physical workload during pregnancy	2.25%	Vitamin D Deficiency	12.20%
Secondhand smoking	0.43%	Hypertension	6.25%
Ambient air pollution	4.43%	Pre-eclampsia	1.29%
Maternal age < 18 & Primiparity	0.39%	Subclinical Hypothyroidism	0.48%
Maternal age 18-35 and Primiparity	5.77%	Anxiety Disorder	3.70%
Birth interval of < 18 months	2.82%	Inflammatory Bowel Disease	0.14%
Birth interval of 18-<24 months	1.67%	Endometriosis	0.20%
Birth interval of over 60 months	1.44%	Adenomyosis	0.28%
Smoking	0.24%	Twin pregnancies	1.97%
		**Total**	57.64%

Tanzania

**Risk Factor**	**PAF**	**Risk Factor**	**PAF**
Malaria	0.74%	Alcohol consumption during pregnancy	0.38%
HIV	2.67%	Short height	1.96%
Chlamydia	0.23%	Low pre-pregnancy BMI	1.70%
Trichomonas vaginalis	2.40%	Low gestational weight gain	11.08%

Heavy Physical workload during pregnancy	3.84%	Vitamin D Deficiency	0.54%
Secondhand smoking	0.35%	Hypertension	6.67%
Ambient air pollution	2.98%	Pre-eclampsia	1.31%
Maternal age < 18 & Primiparity	2.83%	Subclinical Hypothyroidism	0.48%
Maternal age 18-35 and Primiparity	5.75%	Anxiety Disorder	3.69%
Birth interval of < 18 months	1.20%	Inflammatory Bowel Disease	0.01%
Birth interval of 18-<24 months	1.36%	Endometriosis	0.12%
Birth interval of over 60 months	0.12%	Adenomyosis	0.28%
Smoking	0.84%	Twin pregnancies	3.73%
		**Total**	57.25%

Timor-Leste

Risk Factor	PAF	Risk Factor	PAF
Malaria	0.00%	Alcohol consumption during pregnancy	0.05%
HIV	0.08%	Short height	6.32%
Chlamydia	0.18%	Low pre-pregnancy BMI	2.58%
Trichomonas vaginalis	0.73%	Low gestational weight gain	6.68%
Heavy Physical workload during pregnancy	1.18%	Vitamin D Deficiency	11.47%
Secondhand smoking	0.81%	Hypertension	6.37%
Ambient air pollution	2.26%	Pre-eclampsia	0.82%
Maternal age < 18 & Primiparity	0.91%	Subclinical Hypothyroidism	0.45%
Maternal age 18-35 and Primiparity	5.42%	Anxiety Disorder	3.48%
Birth interval of < 18 months	2.51%	Inflammatory Bowel Disease	0.00%
Birth interval of 18-<24 months	1.58%	Endometriosis	0.12%
Birth interval of over 60 months	1.34%	Adenomyosis	0.26%

Smoking	6.37%	Twin pregnancies	1.63%
		**Total**	63.62%

Togo

**Risk Factor**	**PAF**	**Risk Factor**	**PAF**
Malaria	1.46%	Alcohol consumption during pregnancy	0.20%
HIV	1.22%	Short height	0.55%
Chlamydia	0.16%	Low pre-pregnancy BMI	1.50%
Trichomonas vaginalis	1.75%	Low gestational weight gain	10.61%
Heavy Physical workload during pregnancy	3.52%	Vitamin D Deficiency	11.65%
Secondhand smoking	0.19%	Hypertension	6.70%
Ambient air pollution	3.32%	Pre-eclampsia	1.54%
Maternal age < 18 & Primiparity	1.59%	Subclinical Hypothyroidism	0.46%
Maternal age 18-35 and Primiparity	5.51%	Anxiety Disorder	3.53%
Birth interval of < 18 months	0.99%	Inflammatory Bowel Disease	0.01%
Birth interval of 18-<24 months	0.90%	Endometriosis	0.12%
Birth interval of over 60 months	0.18%	Adenomyosis	0.27%
Smoking	0.63%	Twin pregnancies	4.00%
		**Total**	62.53%

Turkmenistan

**Risk Factor**	**PAF**	**Risk Factor**	**PAF**
Malaria	0.00%	Alcohol consumption during pregnancy	0.68%
HIV	0.04%	Short height	0.50%
Chlamydia	0.35%	Low pre-pregnancy BMI	0.79%
Trichomonas vaginalis	1.48%	Low gestational weight gain	7.11%

Heavy Physical workload during pregnancy	2.63%	Vitamin D Deficiency	12.21%
Secondhand smoking	0.29%	Hypertension	5.90%
Ambient air pollution	3.45%	Pre-eclampsia	1.12%
Maternal age < 18 & Primiparity	3.25%	Subclinical Hypothyroidism	0.48%
Maternal age 18-35 and Primiparity	5.77%	Anxiety Disorder	3.70%
Birth interval of < 18 months	1.96%	Inflammatory Bowel Disease	0.14%
Birth interval of 18-<24 months	1.26%	Endometriosis	0.20%
Birth interval of over 60 months	1.32%	Adenomyosis	0.28%
Smoking	0.54%	Twin pregnancies	1.98%
		**Total**	57.46%

Uganda

**Risk Factor**	**PAF**	**Risk Factor**	**PAF**
Malaria	1.51%	Alcohol consumption during pregnancy	0.47%
HIV	2.85%	Short height	0.92%
Chlamydia	0.19%	Low pre-pregnancy BMI	1.53%
Trichomonas vaginalis	1.80%	Low gestational weight gain	11.98%
Heavy Physical workload during pregnancy	3.00%	Vitamin D Deficiency	11.25%
Secondhand smoking	0.19%	Hypertension	6.17%
Ambient air pollution	4.65%	Pre-eclampsia	1.34%
Maternal age < 18 & Primiparity	2.53%	Subclinical Hypothyroidism	0.44%
Maternal age 18-35 and Primiparity	3.22%	Anxiety Disorder	3.42%
Birth interval of < 18 months	1.92%	Inflammatory Bowel Disease	0.01%
Birth interval of 18-<24 months	1.48%	Endometriosis	0.11%
Birth interval of over 60 months	0.12%	Adenomyosis	0.26%

Smoking	1.42%	Twin pregnancies	2.84%
		**Total**	65.63%

Uzbekistan

Risk Factor	PAF	Risk Factor	PAF
Malaria	0.00%	Alcohol consumption during pregnancy	1.01%
HIV	0.04%	Short height	0.78%
Chlamydia	0.34%	Low pre-pregnancy BMI	0.83%
Trichomonas vaginalis	1.45%	Low gestational weight gain	7.06%
Heavy Physical workload during pregnancy	2.64%	Vitamin D Deficiency	12.12%
Secondhand smoking	0.30%	Hypertension	5.92%
Ambient air pollution	3.89%	Pre-eclampsia	1.21%
Maternal age < 18 & Primiparity	0.67%	Subclinical Hypothyroidism	0.48%
Maternal age 18-35 and Primiparity	5.94%	Anxiety Disorder	3.67%
Birth interval of < 18 months	2.36%	Inflammatory Bowel Disease	0.13%
Birth interval of 18-<24 months	1.67%	Endometriosis	0.20%
Birth interval of over 60 months	1.26%	Adenomyosis	0.28%
Smoking	1.93%	Twin pregnancies	2.00%
		**Total**	58.18%

Venezuela

Risk Factor	PAF	Risk Factor	PAF
Malaria	0.11%	Alcohol consumption during pregnancy	0.48%
HIV	0.17%	Short height	4.29%
Chlamydia	0.28%	Low pre-pregnancy BMI	0.34%
Trichomonas vaginalis	1.47%	Low gestational weight gain	5.21%

Heavy Physical workload during pregnancy	2.39%	Vitamin D Deficiency	11.75%
Secondhand smoking	0.35%	Hypertension	3.81%
Ambient air pollution	2.93%	Pre-eclampsia	0.97%
Maternal age < 18 & Primiparity	3.13%	Subclinical Hypothyroidism	0.46%
Maternal age 18-35 and Primiparity	5.55%	Anxiety Disorder	3.56%
Birth interval of < 18 months	1.89%	Inflammatory Bowel Disease	0.01%
Birth interval of 18-<24 months	1.21%	Endometriosis	0.09%
Birth interval of over 60 months	3.07%	Adenomyosis	0.27%
Smoking	5.32%	Twin pregnancies	1.82%
		**Total**	60.93%

Yemen

**Risk Factor**	**PAF**	**Risk Factor**	**PAF**
Malaria	0.20%	Alcohol consumption during pregnancy	0.00%
HIV	0.00%	Short height	4.73%
Chlamydia	0.13%	Low pre-pregnancy BMI	1.59%
Trichomonas vaginalis	0.68%	Low gestational weight gain	6.94%
Heavy Physical workload during pregnancy	0.30%	Vitamin D Deficiency	11.91%
Secondhand smoking	0.40%	Hypertension	7.26%
Ambient air pollution	4.20%	Pre-eclampsia	0.47%
Maternal age < 18 & Primiparity	1.64%	Subclinical Hypothyroidism	0.47%
Maternal age 18-35 and Primiparity	3.53%	Anxiety Disorder	3.61%
Birth interval of < 18 months	3.18%	Inflammatory Bowel Disease	0.04%
Birth interval of 18-<24 months	1.67%	Endometriosis	0.15%
Birth interval of over 60 months	1.35%	Adenomyosis	0.27%

Smoking	3.53%	Twin pregnancies	1.79%
		**Total**	60.02%

Zambia

Risk Factor	PAF	Risk Factor	PAF
Malaria	0.79%	Alcohol consumption during pregnancy	0.42%
HIV	5.27%	Short height	1.22%
Chlamydia	0.16%	Low pre-pregnancy BMI	1.45%
Trichomonas vaginalis	3.89%	Low gestational weight gain	10.09%
Heavy Physical workload during pregnancy	3.11%	Vitamin D Deficiency	11.08%
Secondhand smoking	0.30%	Hypertension	5.83%
Ambient air pollution	2.88%	Pre-eclampsia	1.24%
Maternal age < 18 & Primiparity	3.01%	Subclinical Hypothyroidism	0.44%
Maternal age 18-35 and Primiparity	2.78%	Anxiety Disorder	3.36%
Birth interval of < 18 months	0.95%	Inflammatory Bowel Disease	0.01%
Birth interval of 18-<24 months	1.08%	Endometriosis	0.11%
Birth interval of over 60 months	1.70%	Adenomyosis	0.25%
Smoking	1.66%	Twin pregnancies	3.45%
		**Total**	66.55%

Zimbabwe

Risk Factor	PAF	Risk Factor	PAF
Malaria	0.24%	Alcohol consumption during pregnancy	0.19%
HIV	5.83%	Short height	0.47%
Chlamydia	0.17%	Low pre-pregnancy BMI	1.08%
Trichomonas vaginalis	2.40%	Low gestational weight gain	10.30%

Heavy Physical workload during pregnancy	3.53%	Vitamin D Deficiency	11.31%
Secondhand smoking	0.42%	Hypertension	6.50%
Ambient air pollution	2.66%	Pre-eclampsia	0.83%
Maternal age < 18 & Primiparity	2.61%	Subclinical Hypothyroidism	0.45%
Maternal age 18-35 and Primiparity	3.91%	Anxiety Disorder	4.06%
Birth interval of < 18 months	0.86%	Inflammatory Bowel Disease	0.01%
Birth interval of 18-<24 months	0.68%	Endometriosis	0.12%
Birth interval of over 60 months	2.46%	Adenomyosis	0.26%
Smoking	0.78%	Twin pregnancies	2.96%
		**Total**	65.07%

Reference

1. Villar J, Papageorghiou AT, Pang R, et al. The likeness of fetal growth and newborn size across non-isolated populations in the INTERGROWTH-21st Project: the Fetal Growth Longitudinal Study and Newborn Cross-Sectional Study. Lancet Diabetes Endocrinol 2014;2:781-92.

2. Zhang J, Yu KF. What's the relative risk? A method of correcting the odds ratio in cohort studies of common outcomes. JAMA 1998;280:1690-1.

3. Moher D, Liberati A, Tetzlaff J, Altman DG, PRISMA Group. Preferred reporting items for systematic reviews and meta-analyses: the PRISMA statement. Int J Surg 2010;8:336-41.

4. Guyatt GH, Oxman AD, Vist GE, et al. GRADE: an emerging consensus on rating quality of evidence and strength of recommendations. BMJ 2008;336:924-6.

5. Saito M, Mansoor R, Kennon K, et al. Pregnancy outcomes and risk of placental malaria after artemisinin-based and quinine-based treatment for uncomplicated falciparum malaria in pregnancy: a WorldWide Antimalarial Resistance Network systematic review and individual patient data meta-analysis. BMC medicine 2020;18:1-138.

6. Moore KA, Simpson JA, Wiladphaingern J, et al. Influence of the number and timing of malaria episodes during pregnancy on prematurity and small-for-gestational-age in an area of low transmission. BMC medicine 2017;15:117.

7. Dreyfuss ML, Msamanga GI, Spiegelman D, et al. Determinants of low birth weight among HIV-infected pregnant women in Tanzania. The American journal of clinical nutrition 2001;74:814-26.

8. Dombrowski JG, de Souza RM, Silva NRM, et al. Malaria during pregnancy and newborn outcome in an unstable transmission area in Brazil: A population-based record linkage study. PloS one 2018;13:e0199415.

9. Cates JE, Unger HW, Briand V, et al. Malaria, malnutrition, and birthweight: A meta-analysis using individual participant data. PLoS medicine 2017;14:e1002373.

10. Augusto O, Stergachis A, Dellicour S, et al. First trimester use of artemisinin-based combination therapy and the risk of low birth weight and small for gestational age. Malaria journal 2020;19:144.

11. Wedi COO, Kirtley S, Hopewell S, Corrigan R, Kennedy SH, Hemelaar J. Perinatal outcomes associated with maternal HIV infection: a systematic review and meta-analysis. The lancet HIV 2016;3:e33-48.

12. Uthman OA, Nachega JB, Anderson J, et al. Timing of initiation of antiretroviral therapy and adverse pregnancy outcomes: a systematic review and meta-analysis. The lancet HIV 2017;4:e21-30.

13. Huang QT, Hang LL, Zhong M, Gao YF, Luo ML, Yu YH. Maternal HCV infection is associated with intrauterine fetal growth disturbance: A meta-analysis of observational studies. Medicine (Baltimore) 2016;95:e4777.

14. Mann JR, McDermott S, Gregg A, Gill TJ. Maternal Genitourinary Infection and Small for Gestational Age. American Journal of Perinatology 2009;26:667-72.

15. Olson-Chen C, Balaram K, Hackney DN. Chlamydia trachomatis and Adverse Pregnancy Outcomes: Meta-analysis of Patients With and Without Infection. Matern Child Health J 2018;22:812-21.

16. Cohen I. Improved pregnancy outcome following successful treatment of chlamydial infection. JAMA : the journal of the American Medical Association 1990;263:3160-3.

17. Kazemier BM, Koningstein FN, Schneeberger C, et al. Maternal and neonatal consequences of treated and untreated asymptomatic bacteriuria in pregnancy: a prospective cohort study

with an embedded randomised controlled trial. The Lancet infectious diseases 2015;15:1324-33.

18. Smaill FM, Vazquez JC. Antibiotics for asymptomatic bacteriuria in pregnancy. Cochrane Database Syst Rev 2019;2019:10.1002/14651858.CD000490.pub4.

19. Lee AC, Mullany LC, Quaiyum M, et al. Effect of population-based antenatal screening and treatment of genitourinary tract infections on birth outcomes in Sylhet, Bangladesh (MIST): a cluster-randomised clinical trial. The Lancet global health 2019;7:e148-59.

20. Wimmer G, Pihlstrom BL. A critical assessment of adverse pregnancy outcome and periodontal disease. Journal of clinical periodontology 2008;35:380-97.

21. Iheozor-Ejiofor Z, Middleton P, Esposito M, Glenny AM. Treating periodontal disease for preventing adverse birth outcomes in pregnant women. Cochrane Database Syst Rev 2017;6:CD005297.

22. Iheozor-Ejiofor Z, Middleton P, Esposito M, Glenny AM. Treating periodontal disease for preventing adverse birth outcomes in pregnant women. Cochrane Database Syst Rev 2017;6:CD005297.

23. Bi WG, Emami E, Luo Z, Santamaria C, Wei SQ. Effect of periodontal treatment in pregnancy on perinatal outcomes: a systematic review and meta-analysis. The journal of maternal-fetal & neonatal medicine 2019;:1-10.

24. Yatich NJ, Jolly PE, Funkhouser E, et al. The Effect of Malaria and Intestinal Helminth Coinfection on Birth Outcomes in Kumasi, Ghana. American Journal of Tropical Medicine and Hygiene 2010;82:28-34.

25. Thayer WM, Clermont A, Walker N. Effects of deworming on child and maternal health: a literature review and meta-analysis. BMC public health 2017;17:830.

26. Silver B, Guy R, Kaldor J, Jamil M, Rumbold A. Trichomonas vaginalis as a Cause of Perinatal Morbidity: A Systematic Review and Meta-Analysis. Sexually transmitted diseases 2014;41:369-76.

27. Vedmedovska N, Rezeberga D, Donder GGG. Is abnormal vaginal microflora a risk factor for intrauterine fetal growth restriction? Asian Pacific Journal of Reproduction 2015;4:313-6.

28. April Sheppard. Cochrane Library. 2015;.

29. Baqui AH, Lee ACC, Koffi AK, et al. Prevalence of and risk factors for abnormal vaginal flora and its association with adverse pregnancy outcomes in a rural district in north-east Bangladesh. Acta obstetricia et gynecologica Scandinavica 2019;98:309-19.

30. Dawood FS, Kittikraisak W, Patel A, et al. Incidence of influenza during pregnancy and association with pregnancy and perinatal outcomes in three middle-income countries: a multisite prospective longitudinal cohort study. Lancet Infect Dis 2021;21:97-106.

31. Nunes MC, Aqil AR, Omer SB, Madhi SA. The Effects of Influenza Vaccination during Pregnancy on Birth Outcomes: A Systematic Review and Meta-Analysis. Am J Perinatol 2016;33:1104-14.

32. Giles ML, Krishnaswamy S, Macartney K, Cheng A. The safety of inactivated influenza vaccines in pregnancy for birth outcomes: a systematic review. Hum Vaccin Immunother 2019;15:687-99.

33. Prata-Barbosa A, Martins MM, Guastavino AB, Cunha, A J L A D. Effects of Zika infection on growth. J Pediatr (Rio J) 2019;95 Suppl 1:30-41.

34. Brasil P, Pereira JP, Moreira ME, et al. Zika Virus Infection in Pregnant Women in Rio de Janeiro. N Engl J Med 2016;375:2321-34.

35. Watson-Jones D, Changalucha J, Gumodoka B, et al. Syphilis in pregnancy in Tanzania. I. Impact of maternal syphilis on outcome of pregnancy. J Infect Dis 2002;186:940-7.

36. Miller E, Cradock-Watson JE, Pollock TM. Consequences of confirmed maternal rubella at successive stages of pregnancy. Lancet 1982;2:781-4.

37. Urrutia JJ, Mata LJ, Trent F, Cruz JR, Villatoro E, Alexander RE. Infection and low birth weight in a developing country. A study in an Indian village of Guatemala. Am J Dis Child 1975;129:558-61.

38. Ueda K, Hisanaga S, Nishida Y, Shepard TH. Low-birth-weight and congenital rubella syndrome: effect of gestational age at time of maternal rubella infection. Clin Pediatr (Phila) 1981;20:730-3.

39. Bar-Oz B, Levichek Z, Moretti ME, Mah C, Andreou S, Koren G. Pregnancy outcome following rubella vaccination: a prospective controlled study. Am J Med Genet A 2004;130A:52-4.

40. Ogbuanu IU, Zeko S, Chu SY, et al. Maternal, fetal, and neonatal outcomes associated with measles during pregnancy: Namibia, 2009-2010. Clin Infect Dis 2014;58:1086-92.

41. Freeman K, Oakley L, Pollak A, et al. Association between congenital toxoplasmosis and preterm birth, low birthweight and small for gestational age birth. BJOG 2005;112:31-7.

42. Gibson CS, Goldwater PN, MacLennan AH, et al. Fetal exposure to herpesviruses may be associated with pregnancy-induced hypertensive disorders and preterm birth in a Caucasian population. BJOG 2008;115:492-500.

43. Palmer KT, Bonzini M, Harris EC, Linaker C, Bonde JP. Work activities and risk of prematurity, low birthweight and pre-eclampsia: an updated review with meta-analysis. Occupational and environmental medicine (London, England) 2013;70:213-222.

44. Cai C, Vandermeer B, Khurana R, et al. The impact of occupational activities during pregnancy on pregnancy outcomes: a systematic review and metaanalysis. American journal of obstetrics and gynecology 2019;222:224-38.

45. Donovan B, Spracklen C, Schweizer M, Ryckman K, Saftlas A. Intimate partner violence during pregnancy and the risk for adverse infant outcomes: a systematic review and meta-analysis. BJOG : an international journal of obstetrics and gynaecology 2016;123:1289-99.

46. Hill A, Pallitto C, McCleary-Sills J, Garcia-Moreno C. A systematic review and meta-analysis of intimate partner violence during pregnancy and selected birth outcomes. International journal of gynecology and obstetrics 2016;133:269-76.

47. Buffa G, Dahan S, Sinclair I, et al. Prenatal stress and child development: A scoping review of research in low- and middle-income countries. PloS one 2018;13:e0207235.

48. Salmasi G, Grady R, Jones J, McDonald SD. Environmental tobacco smoke exposure and perinatal outcomes: a systematic review and meta-analyses. Acta obstetricia et gynecologica Scandinavica 2010;89:423-41.

49. Leonardi-Bee J, Smyth A, Britton J, Coleman T. Environmental tobacco smoke and fetal health: systematic review and meta-analysis. Arch Dis Child Fetal Neonatal Ed 2008;93:351.

50. Philips EM, Santos S, Trasande L, et al. Changes in parental smoking during pregnancy and risks of adverse birth outcomes and childhood overweight in Europe and North America: An individual participant data meta-analysis of 229,000 singleton births. PLoS medicine 2020;17:e1003182.

51. Been JV, Mackay DF, Millett C, et al. Smoke-free legislation and paediatric hospitalisations for acute respiratory tract infections: national quasi-experimental study with unexpected findings and important methodological implications. Tob Control 2018;27:e160-6.

52. Amegah AK, Quansah R, Jaakkola JJK. Household Air Pollution from Solid Fuel Use and Risk of Adverse Pregnancy Outcomes: A Systematic Review and Meta-Analysis of the Empirical Evidence. PloS one 2014;9:e113920.

53. Tielsch JM, Katz J, Thulasiraj RD, et al. Exposure to indoor biomass fuel and tobacco smoke and risk of adverse reproductive outcomes, mortality, respiratory morbidity and growth among newborn infants in south India. International journal of epidemiology 2009;38:1351-63.

54. Hussein H, Shamsipour M, Yunesian M, Hasanvand MS, Fotouhi A. Association of adverse birth outcomes with exposure to fuel type use: A prospective cohort study in the northern region of Ghana. Heliyon 2020;6:e04169.

55. Jiang M, Qiu J, Zhou M, et al. Exposure to cooking fuels and birth weight in Lanzhou, China: a birth cohort study. BMC public health 2015;15:712.

56. Wylie BJ, Coull BA, Hamer DH, et al. Impact of biomass fuels on pregnancy outcomes in central East India. Environmental health 2014;13:1.

57. Katz J, Tielsch JM, Khatry SK, et al. Impact of Improved Biomass and Liquid Petroleum Gas Stoves on Birth Outcomes in Rural Nepal: Results of 2 Randomized Trials. Glob Health Sci Pract 2020;8:372-82.

58. Zhu X, Liu Y, Chen Y, Yao C, Che Z, Cao J. Maternal exposure to fine particulate matter (PM2.5) and pregnancy outcomes: a meta-analysis. Environ Sci Pollut Res 2014;22:3383-96.

59. Wang Q, Benmarhnia T, Li C, et al. Seasonal analyses of the association between prenatal ambient air pollution exposure and birth weight for gestational age in Guangzhou, China. The Science of the total environment 2019;649:526-34.

60. Akaraci S, Feng X, Suesse T, Jalaludin B, Astell-Burt T. A Systematic Review and Meta-Analysis of Associations between Green and Blue Spaces and Birth Outcomes. International journal of environmental research and public health 2020;17:2949.

61. Mamluk L, Edwards HB, Savović J, et al. Low alcohol consumption and pregnancy and childhood outcomes: time to change guidelines indicating apparently 'safe' levels of alcohol during pregnancy? A systematic review and meta-analyses. BMJ open 2017;7:e015410.

62. Patra J, Bakker R, Irving H, Jaddoe VWV, Malini S, Rehm J. Dose-response relationship between alcohol consumption before and during pregnancy and the risks of low birth weight, preterm birth and small-size-for-gestational age (SGA) – A systematic review and meta-analyses. BJOG : an international journal of obstetrics and gynaecology 2011;118:1411-1421.

63. Zhan Y, Xiao Y, Guan T, Zhang S, Jiang Y. Relationship between gestational acrylamide exposure and offspring's growth: a systematic review and meta-analysis of cohort studies. Public health nutrition 2020;23:1791-9.

64. Kozuki N, Lee AC, Silveira MF, et al. The associations of parity and maternal age with small-for-gestational-age, preterm, and neonatal and infant mortality: a meta-analysis. BMC public health 2013;13:S2.

65. Kozuki N, Lee AC, Silveira MF, et al. The associations of birth intervals with small-for-gestational-age, preterm, and neonatal and infant mortality: a meta-analysis. BMC public health 2013;13:S3.

66. Ciobanu A, Rouvali A, Syngelaki A, Akolekar R, Nicolaides KH. Prediction of small for gestational age neonates: screening by maternal factors, fetal biometry, and biomarkers at 35–37 weeks' gestation. American journal of obstetrics and gynecology 2019;220:486.e1,486.e11.

67. Taylor LK, Lee YYC, Lim K, Simpson JM, Roberts CL, Morris J. Potential prevention of small for gestational age in Australia: a population-based linkage study. BMC pregnancy and childbirth 2013;13:210.

68. Saccone G, Perriera L, Berghella V. Prior uterine evacuation of pregnancy as independent risk factor for preterm birth: a systematic review and metaanalysis. Am J Obstet Gynecol 2016;214:572-91.

69. Shah P, Zao J. Induced termination of pregnancy and low birthweight and preterm birth: a systematic review and meta-analyses. BJOG : an international journal of obstetrics and gynaecology 2009;116:1425-42.

70. Kozuki N, Katz J, Lee AC, et al. Short Maternal Stature Increases Risk of Small-for-Gestational-Age and Preterm Births in Low- and Middle-Income Countries: Individual Participant Data Meta-Analysis and Population Attributable Fraction. The Journal of nutrition 2015;145:2542-50.

71. Carducci B, Keats EC, Bhutta ZA. Zinc supplementation for improving pregnancy and infant outcome. Cochrane Database Syst Rev 2021;3:CD000230.

72. Ota E, Mori R, Middleton P, et al. Zinc supplementation for improving pregnancy and infant outcome. Cochrane library 2015;2015:CD000230.

73. Young MF, Oaks BM, Tandon S, Martorell R, Dewey KG, Wendt AS. Maternal hemoglobin concentrations across pregnancy and maternal and child health: a systematic review and meta-analysis. Annals of the New York Academy of Sciences 2019;1450:47-68.

74. Rahman MM, Abe SK, Rahman MS, et al. Maternal anemia and risk of adverse birth and health outcomes in low- and middle-income countries: systematic review and meta-analysis. The American journal of clinical nutrition 2016;103:495-504.

75. Haider BA, Olofin I, Wang M, et al. Anaemia, prenatal iron use, and risk of adverse pregnancy outcomes: systematic review and meta-analysis. BMJ 2013;346:f3443.

76. Pena-Rosas JP, De-Regil LM, Garcia-Casal MN, Dowswell T. Daily oral iron supplementation during pregnancy. Cochrane Database Syst Rev 2015;(7):CD004736. doi:CD004736.

77. Goto E. Dose-response association between maternal body mass index and small for gestational age: a meta-analysis. J Matern Fetal Neonatal Med 2017;30:213-8.

78. Liu P, Xu L, Wang Y, et al. Association between perinatal outcomes and maternal pre-pregnancy body mass index. Obes Rev 2016;17:1091-102.

79. Han Z, Mulla S, Beyene J, Liao G, McDonald SD. Maternal underweight and the risk of preterm birth and low birth weight: a systematic review and meta-analyses. International journal of epidemiology 2011;40:65-101.

80. Kramer MS, Kakuma R. Energy and protein intake in pregnancy. Cochrane Database Syst Rev 2003;(4):CD000032. doi:CD000032.

81. Imdad A, Bhutta ZA. Effect of balanced protein energy supplementation during pregnancy on birth outcomes. BMC Public Health 2011;11 Suppl 3:S17-.

82. Yu Z, Han S, Zhu J, Sun X, Ji C, Guo X. Pre-pregnancy body mass index in relation to infant birth weight and offspring overweight/obesity: a systematic review and meta-analysis. PLoS One 2013;8:e61627.

83. Goldstein RF, Abell SK, Ranasinha S, et al. Association of Gestational Weight Gain With Maternal and Infant Outcomes: A Systematic Review and Meta-analysis. JAMA : the journal of the American Medical Association 2017;317:2207-25.

84. Thorne-Lyman AL, Fawzi WW. Vitamin A and Carotenoids During Pregnancy and Maternal, Neonatal and Infant Health Outcomes: a Systematic Review and Meta-Analysis. Paediatric and perinatal epidemiology 2012;26:36-54.

85. Hofmeyr GJ, Lawrie TA, Atallah AN, Torloni MR. Calcium supplementation during pregnancy for preventing hypertensive disorders and related problems. Cochrane Database Syst Rev 2018;10:CD001059.

86. Hofmeyr GJ, Lawrie TA, Atallah AN, Torloni MR. Calcium supplementation during pregnancy for preventing hypertensive disorders and related problems. Cochrane Database Syst Rev 2018;10:CD001059.

87. Imdad A, Jabeen A, Bhutta ZA. Role of calcium supplementation during pregnancy in reducing risk of developing gestational hypertensive disorders: a meta-analysis of studies from developing countries. BMC public health 2011;11 Suppl 3:S18.

88. Hu Z, Tang L, Xu H. Maternal Vitamin D Deficiency and the Risk of Small for Gestational Age: A Meta-analysis. Iranian journal of public health 2018;47:1785-95.

89. Maugeri A, Barchitta M, Blanco I, Agodi A. Effects of Vitamin D Supplementation During Pregnancy on Birth Size: A Systematic Review and Meta-Analysis of Randomized Controlled Trials. Nutrients 2019;11:442.

90. Bialy L, Fenton T, Shulhan-Kilroy J, Johnson DW, McNeil DA, Hartling L. Vitamin D supplementation to improve pregnancy and perinatal outcomes: an overview of 42 systematic reviews. BMJ Open 2020;10:e032626.

91. Roth DE, Morris SK, Zlotkin S, et al. Vitamin D Supplementation in Pregnancy and Lactation and Infant Growth. N Engl J Med 2018;379:535-46.

92. Cohen JM, Beddaoui M, Kramer MS, Platt RW, Basso O, Kahn SR. Maternal Antioxidant Levels in Pregnancy and Risk of Preeclampsia and Small for Gestational Age Birth: A Systematic Review and Meta-Analysis. PloS one 2015;10:e0135192.

93. Rumbold A, Ota E, Hori H, Miyazaki C, Crowther CA. Vitamin E supplementation in pregnancy. Cochrane Database Syst Rev 2015;(9):CD004069. doi:CD004069.

94. Rumbold A, Ota E, Nagata C, Shahrook S, Crowther CA. Vitamin C supplementation in pregnancy. Cochrane Database Syst Rev 2015;(9):CD004072. doi:CD004072.

95. Snart CJP, Threapleton DE, Keeble C, et al. Maternal iodine status, intrauterine growth, birth outcomes and congenital anomalies in a UK birth cohort. BMC medicine 2020;18:1-132.

96. Snart C, Keeble C, Taylor E, et al. Maternal Iodine Status and Associations with Birth Outcomes in Three Major Cities in the United Kingdom. Nutrients 2019;11:441.

97. Purdue-Smithe AC, Männistö T, Bell GA, et al. The Joint Role of Thyroid Function and Iodine Status on Risk of Preterm Birth and Small for Gestational Age: A Population-Based Nested Case-Control Study of Finnish Women. Nutrients 2019;11:2573.

98. Zimmermann MB, Andersson M. Update on iodine status worldwide. Current opinion in endocrinology, diabetes, and obesity 2012;19:382-7.

99. Ota E, Ganchimeg T, Morisaki N, et al. Risk Factors and Adverse Perinatal Outcomes among Term and Preterm Infants Born Small-for-Gestational-Age: Secondary Analyses of the WHO Multi-Country Survey on Maternal and Newborn Health. PloS one 2014;9:e105155.

100. Palatnik A, Grobman WA, Miller ES. 169: Is a history of preeclampsia associated with an increased risk of small for gestational age in a future pregnancy? American Journal of Obstetrics and Gynecology 2016;214:S107-8.

101. RØNNAUG A. ØDEGÅRD, LARS J. VATTEN, STEIN TORE NILSEN, KJELL Å. SALVESEN, RIGMOR AUSTGULEN. Preeclampsia and Fetal Growth. 2000;.

102. Duley L, Henderson-Smart DJ, Meher S, King JF. Antiplatelet agents for preventing pre-eclampsia and its complications. Cochrane Database Syst Rev 2007;(2):CD004659. doi:CD004659.

103. MORRIS RK, OLIVER EA, MALIN G, KHAN KS, MEADS C. Effectiveness of interventions for the prevention of small-for-gestational age fetuses and perinatal mortality: a review of systematic reviews. Acta obstetricia et gynecologica Scandinavica 2013;92:143-51.

104. Hartling L, Dryden DM, Guthrie A, Muise M, Vandermeer B, Donovan L. Benefits and Harms of Treating Gestational Diabetes Mellitus: A Systematic Review and Meta-analysis for the U.S.

Preventive Services Task Force and the National Institutes of Health Office of Medical Applications of Research. Annals of internal medicine 2013;159:123-9.

105. Magee LA, Duley L. Oral beta-blockers for mild to moderate hypertension during pregnancy. Cochrane Database Syst Rev 2003;(3):CD002863. doi:CD002863.

106. von Dadelszen P, Ornstein MP, Bull SB, Logan AG, Koren G, Magee LA. Fall in mean arterial pressure and fetal growth restriction in pregnancy hypertension: a meta-analysis. Lancet 2000;355:87-92.

107. Bellos I, Pergialiotis V, Papapanagiotou A, Loutradis D, Daskalakis G. Comparative efficacy and safety of oral antihypertensive agents in pregnant women with chronic hypertension: a network metaanalysis. American journal of obstetrics and gynecology 2020;223:525-37.

108. Yu HF, Chen HS, Rao DP, Gong J. Association between polycystic ovary syndrome and the risk of pregnancy complications: A PRISMA-compliant systematic review and meta-analysis. Medicine (Baltimore) 2016;95:e4863.

109. Derakhshan A, Peeters RP, Taylor PN, et al. Association of maternal thyroid function with birthweight: a systematic review and individual-participant data meta-analysis. The lancet. Diabetes & endocrinology 2020;8:501-10.

110. Tong Z, Xiaowen Z, Baomin C, et al. The Effect of Subclinical Maternal Thyroid Dysfunction and Autoimmunity on Intrauterine Growth Restriction: A Systematic Review and Meta-Analysis. Medicine (Baltimore) 2016;95:e3677.

111. Grigoriadis S, Graves L, Peer M, et al. Maternal Anxiety During Pregnancy and the Association With Adverse Perinatal Outcomes: Systematic Review and Meta-Analysis. J Clin Psychiatry 2018;79:10.4088/JCP.17r12011.

112. Grigoriadis S, Graves L, Peer M, et al. Pregnancy and Delivery Outcomes Following Benzodiazepine Exposure: A Systematic Review and Meta-analysis. Can J Psychiatry 2020;:706743720904860.

113. Zhao X, Liu Q, Cao S, et al. A meta-analysis of selective serotonin reuptake inhibitors (SSRIs) use during prenatal depression and risk of low birth weight and small for gestational age. Journal of affective disorders 2018;241:563-70.

114. O'Toole A, Nwanne O, Tomlinson T. Inflammatory Bowel Disease Increases Risk of Adverse Pregnancy Outcomes: A Meta-Analysis. Dig Dis Sci 2015;60:2750-61.

115. Aukes AM, Yurtsever FN, Boutin A, Visser MC, Groot CJM. Associations Between Migraine and Adverse Pregnancy Outcomes: Systematic Review and Meta-analysis. 2019;.

116. Skajaa N, Szepligeti SK, Xue F, et al. Pregnancy, Birth, Neonatal, and Postnatal Neurological Outcomes After Pregnancy With Migraine. Headache 2019;59:869-79.

117. Bruun MR, Arendt LH, Forman A, Ramlau-Hansen CH. Endometriosis and adenomyosis are associated with increased risk of preterm delivery and a small-for-gestational-age child: a systematic review and meta-analysis. Acta obstetricia et gynecologica Scandinavica 2018;97:1073-90.

118. Yang J, Savitz DA. The effect of vaginal bleeding during pregnancy on preterm and small-for-gestational-age births: US National Maternal and Infant Health Survey, 1988. Paediatr Perinat Epidemiol 2001;15:34-9.

119. SIPILÄ P, HARTIKAINEN-SORRI A-, OJA H, WENDT L. Perinatal outcome of pregnancies complicated by vaginal bleeding. BJOG : an international journal of obstetrics and gynaecology 1992;99:959-63.

120. Strobino B, Pantel-Silverman J. GESTATIONAL VAGINAL BLEEDING AND PREGNANCY OUTCOME. American journal of epidemiology 1989;129:806-15.

121. Balayla J, Desilets J, Shrem G. Placenta previa and the risk of intrauterine growth restriction (IUGR): a systematic review and meta-analysis. Journal of Perinatal Medicine 2019;47:577-84.

122. Vahanian SA, Lavery JA, Ananth CV, Vintzileos A. Placental implantation abnormalities and risk of preterm delivery: a systematic review and metaanalysis. American journal of obstetrics and gynecology 2015;213:S78-90.

123. Wang A, Liu C, Wang Y, et al. Pregnancy Outcomes After Human Papillomavirus Vaccination in Periconceptional Period or During Pregnancy: A Systematic Review and Meta-analysis. Human vaccines & immunotherapeutics 2019;16:1-9.

124. Volpe G, Ioannou C, Cavallaro A, Vannuccini S, Ruiz-Martinez S, Impey L. The influence of fetal sex on the antenatal diagnosis of small for gestational age. The journal of maternal-fetal & neonatal medicine 2018;32:1832-7.

125. Teixeira MPC, Queiroga TPR, Mesquita MdA. Frequency and risk factors for the birth of small-for-gestational-age newborns in a public maternity hospital. Einstein (São Paulo, Brazil) 2016;14:317-23.

126. Muhihi A, Sudfeld CR, Smith ER, et al. Risk factors for small-for-gestational-age and preterm births among 19,269 Tanzanian newborns. BMC pregnancy and childbirth 2016;16:110.

127. Vogel JP, Torloni MR, Seuc A, et al. Maternal and Perinatal Outcomes of Twin Pregnancy in 23 Low- and Middle-Income Countries. PloS one 2013;8:e70549.

128. Sun L, Chen X, Wen SW, Fung KFK, Yang Q, Walker MC. Perinatal Outcomes of Normal Cotwins in Twin Pregnancies with One Structurally Anomalous Fetus: A Population-Based Retrospective Study. American Journal of Perinatology 2009;26:51.

129. Sun L, Jiang LX, Chen HZ. Obstetric outcome of vanishing twins syndrome: a systematic review and meta-analysis. Arch Gynecol Obstet 2017;295:559-67.

130. Pinborg A, Lidegaard O, Freiesleben N, Andersen AN. Vanishing twins: a predictor of small-for-gestational age in IVF singletons. Hum Reprod 2007;22:2707-14.

131. Shebl O, Ebner T, Sommergruber M, Sir A, Tews G. Birth weight is lower for survivors of the vanishing twin syndrome: a case-control study. Fertil Steril 2008;90:310-4.

132. Anonymous WORLD MALARIA REPORT. 2019;.

133. Anonymous World Population Prospects 2019.

134. AIDSinfo UNAIDS. Anonymous . (, at http://aidsinfo.unaids.org).

135. Global Health Data Exchange

. Anonymous . (, at http://ghdx.healthdata.org).

136. Anonymous World Development Indicators, 2020.

137. Ortiz-Ospina E, Tzvetkova S. Working women: Key facts and trends in female labor force participation, 2017.

138. GBD 2015 Tobacco Collaborators. Smoking prevalence and attributable disease burden in 195 countries and territories, 1990-2015: a systematic analysis from the Global Burden of Disease Study 2015. Lancet 2017;389:1885-906.

139. Ritchie H, Roser M. Indoor Air Pollution. 2013;.

140. Anonymous World Urbanization Prospects 2018.

141. GBD 2015 Risk Factors Collaborators. Global, regional, and national comparative risk assessment of 79 behavioural, environmental and occupational, and metabolic risks or clusters of risks, 1990-2015: a systematic analysis for the Global Burden of Disease Study 2015. Lancet 2016;388:1659-724.

142. STATcompiler. Anonymous . (Accessed Dec 23, 2020, at https://www.statcompiler.com/en/).

143. Popova S, Lange S, Probst C, Gmel G, Rehm J. Estimation of national, regional, and global prevalence of alcohol use during pregnancy and fetal alcohol syndrome: a systematic review and meta-analysis. Lancet Glob Health 2017;5:e290-9.

144. Anonymous Global status report on alcohol and health 2018, 2018.

145. Stevens GA, Finucane MM, De-Regil LM, et al. Global, regional, and national trends in haemoglobin concentration and prevalence of total and severe anaemia in children and pregnant and non-pregnant women for 1995-2011: a systematic analysis of population-representative data. Lancet Glob Health 2013;1:16.

146. Finucane MM, Stevens GA, Cowan MJ, et al. National, regional, and global trends in body-mass index since 1980: systematic analysis of health examination surveys and epidemiological studies with 960 country-years and 9.1 million participants. Lancet 2011;377:557-67.

147. Goldstein RF, Abell SK, Ranasinha S, et al. Gestational weight gain across continents and ethnicity: systematic review and meta-analysis of maternal and infant outcomes in more than one million women. BMC Med 2018;16:153-1.

148. Asefa F, Cummins A, Dessie Y, Hayen A, Foureur M. Gestational weight gain and its effect on birth outcomes in sub-Saharan Africa: Systematic review and meta-analysis. PLoS One 2020;15:e0231889.

149. Palacios C, Gonzalez L. Is vitamin D deficiency a major global public health problem? J Steroid Biochem Mol Biol 2014;144 Pt A:138-45.

150. Saraf R, Morton SM, Camargo CA, Grant CC. Global summary of maternal and newborn vitamin D status - a systematic review. Matern Child Nutr 2016;12:647-68.

151. Anonymous World Health Data Platform : WHO.

152. Abalos E, Cuesta C, Grosso AL, Chou D, Say L. Global and regional estimates of preeclampsia and eclampsia: a systematic review. Eur J Obstet Gynecol Reprod Biol 2013;170:1-7.

153. Kim YA, Park YJ. Prevalence and risk factors of subclinical thyroid disease. Endocrinol Metab (Seoul) 2014;29:20-9.

154. Teng W, Shan Z, Patil-Sisodia K, Cooper DS. Hypothyroidism in pregnancy. Lancet Diabetes Endocrinol 2013;1:228-37.

155. Fisher J, Cabral de Mello M, Patel V, et al. Prevalence and determinants of common perinatal mental disorders in women in low- and lower-middle-income countries: a systematic review. Bull World Health Organ 2012;90:139G-49G.

156. Ng SC, Shi HY, Hamidi N, et al. Worldwide incidence and prevalence of inflammatory bowel disease in the 21st century: a systematic review of population-based studies. Lancet 2018;390:2769-78.

157. Vercellini P, Consonni D, Dridi D, Bracco B, Frattaruolo MP, Somigliana E. Uterine adenomyosis and in vitro fertilization outcome: a systematic review and meta-analysis. Hum Reprod 2014;29:964-77.

158. Smits J, Monden C. Twinning across the Developing World. PLoS One 2011;6:e25239.
